# Experimental randomness amplification

**DOI:** 10.1038/s41586-026-10521-8

**Published:** 2026-05-27

**Authors:** Anatoly Kulikov, Simon Storz, Josua D. Schär, Martin Sandfuchs, Ramona Wolf, Florence Berterottière, Christoph Hellings, Andreas Wallraff, Renato Renner

**Affiliations:** 1 Department of Physics, ETH Zurich, Zurich, Switzerland; 2 Quantum Center, ETH Zurich, Zurich, Switzerland; 3 Naturwissenschaftlich-Technische Fakultät, Universität Siegen, Siegen, Germany; 4 Universität Innsbruck, Institut für Theoretische Physik, Innsbruck, Austria

**Keywords:** Quantum information, Computer science, Qubits, Information theory and computation

## Abstract

Realistic quantum information processing devices are inherently imperfect, leading to computational errors that require quantum error correction. Likewise, random bits generated by such devices are flawed and must be enhanced to be usable for applications such as generating cryptographic keys. This enhancement of randomness quality is achieved through a protocol known as randomness amplification^1^. Here we report on an experiment that implements such a protocol. Randomness amplification is device-independent, making no assumptions about the internal workings of the quantum devices. It requires executing a loophole-free Bell test^2–4^ within a specific parameter regime that involves both a high Bell violation and a high repetition rate. The experimental demonstration is made possible by a combination of theoretical advances, which allow for protocols with an experimentally realistic parameter regime, and experimental progress that achieves this regime with superconducting circuits. Crucially, randomness amplification has been proven to be impossible by purely classical means^5^. This experiment therefore demonstrates a definitive quantum advantage—leveraging quantum technology to accomplish a task unattainable by classical information processing.

## Abstract (web summary)

An experimental realization of device-independent randomness amplification is demonstrated using superconducting qubits, in which a source of weak, correlated randomness is converted into virtually perfect random bits, certified by a Bell test.

## Main

Although many physical processes appear random to us, it turns out to be challenging to produce high-quality randomness—bits for which it is guaranteed that no one can predict them^6,7^. From a theoretical viewpoint, the best possible methods today to generate random bits use quantum processes, exploiting the fact that the outcomes of certain measurements are fundamentally unpredictable^8^. However, the problem with quantum random number generators is that, for any real-world implementation with experimental imperfections, this randomness is not perfect—the probability that the next bit, 0 or 1, can be correctly predicted might deviate from the ideal value of 1/2. The amount of this deviation is called the bias. Post-processing techniques can mitigate this bias, but only if it remains constant over time^7^. However, this is usually not the case, as the bits generated by an imperfect source are generally correlated. In this case, the bias of a bit depends on the previous bits, and thus fluctuates. This is not merely a theoretical problem: correlations have been found in experimental tests of commercially available random number generators^9^. Fundamentally, this problem cannot be solved using any classical post-processing methods, as a seminal result by Santha and Vazirani^5^ shows. In contrast, there exist quantum methods that can turn an initial imperfect random string into a perfectly random bitstring, that is, one without any bias. This task is called randomness amplification. In this work, we present an experimental demonstration of a randomness amplification protocol.

To understand the requirements for perfect randomness, it is helpful to consider a practical use case: the generation of keys in today’s cryptographic systems. Investigations into openly accessible public RSA^10^ keys have revealed that a non-negligible proportion of the corresponding private keys, which are supposed to remain secret even if the public keys are known, could be reconstructed^11,12^. This problem could be traced back to the randomness from which the keys were generated. Some of this randomness was predictable owing to imperfections in the process generating it. As a consequence, parts of the private keys, which were computed from this randomness, were also predictable, which made it possible to reconstruct these keys.

Unpredictability is therefore a crucial requirement for randomness to be useful in practical applications. Yet, unpredictability is not an inherent property of a bitstring itself, but rather of the process that generates it. As such, it cannot be verified through statistical tests. For example, consider a book filled with bits generated by a high-quality random number generator^13^. While these bits may show strong statistical properties (such as not containing any patterns), they are unsuitable for use in cryptography, for example, the generation of private keys, as anyone with access to the book could easily break the encryption.

In theory, there do exist unpredictable processes: a simple example is a photon hitting a 50:50 beam splitter, followed by a measurement to determine which path the photon has taken. According to quantum theory, it is impossible to predict the output bit with probability more than 1/2 (ref. ^8^). We call such a bit perfectly random. However, as mentioned earlier, any practical implementation will inevitably deviate from this ideal. We can thus not guarantee that the output bit of the measurement is perfectly random. The goal of randomness amplification is to address this issue by transforming imperfect random bits into perfect ones.

To achieve this goal, randomness amplification protocols make use of a Bell test. Bell tests consist of measurements performed on entangled systems. Their purpose is to prove that, under natural locality assumptions, there cannot exist any variables that determine the outcomes of these measurements^14–19^. The reasoning is then as follows: if it were possible to predict the outcomes, then this prediction would constitute such a variable—one that is, however, ruled out by the Bell test. Yet, the unpredictable randomness of the measurement outcomes does not come for free, as a Bell test also consumes randomness. Indeed, an important requirement is that the measurements to be performed are chosen at random from a set of possible measurements.

**Fig. 1:**
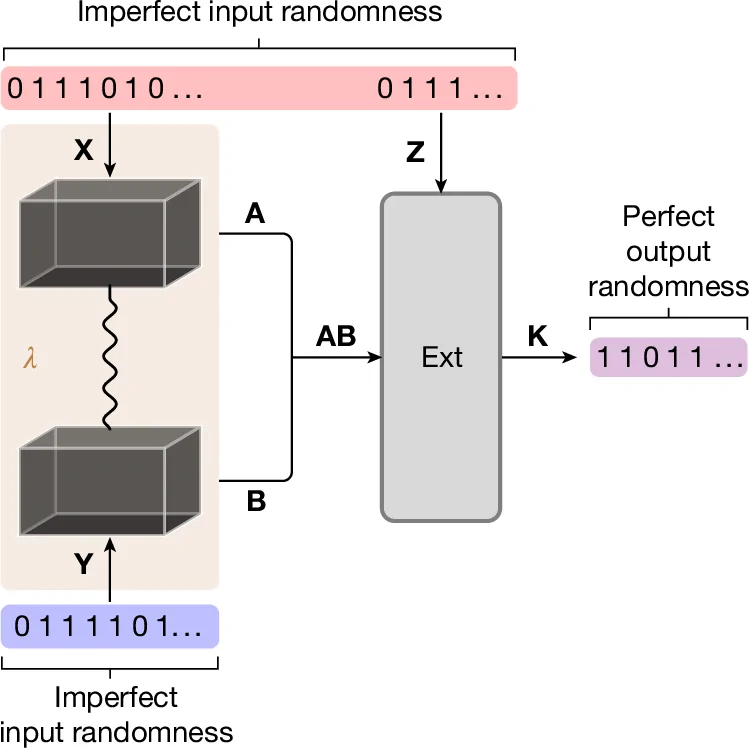
Schematic diagram of the randomness amplification protocol. Two imperfect SV sources^5^ are used to provide input randomness (strings **X** and **Y**) to a two-node untrusted black-box device *λ*, which is used to implement a Bell test. Each node performs a local measurement on an entangled state to produce the outputs **A** and **B**. We do not make any assumptions about the inner workings of the device *λ* and attribute all possible side information to the adversary. If the Bell test succeeds (see main text), we run a randomness extractor (Ext) with an additional input (string **Z**) from one of the SV sources to generate the amplified random string **K**.

In a randomness amplification protocol^1,20^, the initial Bell test is performed using imperfect random bits to choose the measurement settings. Crucially, the violation of an appropriately chosen Bell inequality (see Methods for details) can still be used to guarantee some unpredictability of the measurement results. Despite not being completely unpredictable, the measurement results are only weakly correlated to the source of input randomness. In a second step, one exploits this independence property using a technique from computer science known as two-source extractor^21,22^. Such an extractor can generate perfect random bits from two independent inputs. Hence, feeding the extractor with the initial imperfect randomness as well as the outcomes of the Bell test, achieves the goal. The randomness amplification protocol is schematically visualized in Fig. 1.

One may worry that, with a real-world implementation of randomness amplification, we run into the problem we aimed to avoid, namely, that the implementation may not be ideal, in the sense that it deviates from its theoretical specification. However, randomness amplification can tolerate largely uncharacterized devices, provided that they meet certain weak assumptions (see Supplementary Information section V for a detailed list). In the language of Bell tests, this requires the implementation to be loophole-free (see Methods for a discussion of possible experimental loopholes). We emphasize that, in contrast to computational randomness generation^23^, we also do not need to make any assumptions regarding the hardness of certain computational problems—the randomness we generate provides information-theoretic security. This is essential to make the randomness usable for information-theoretically secure cryptographic schemes, such as quantum key distribution.

**Fig. 2:**
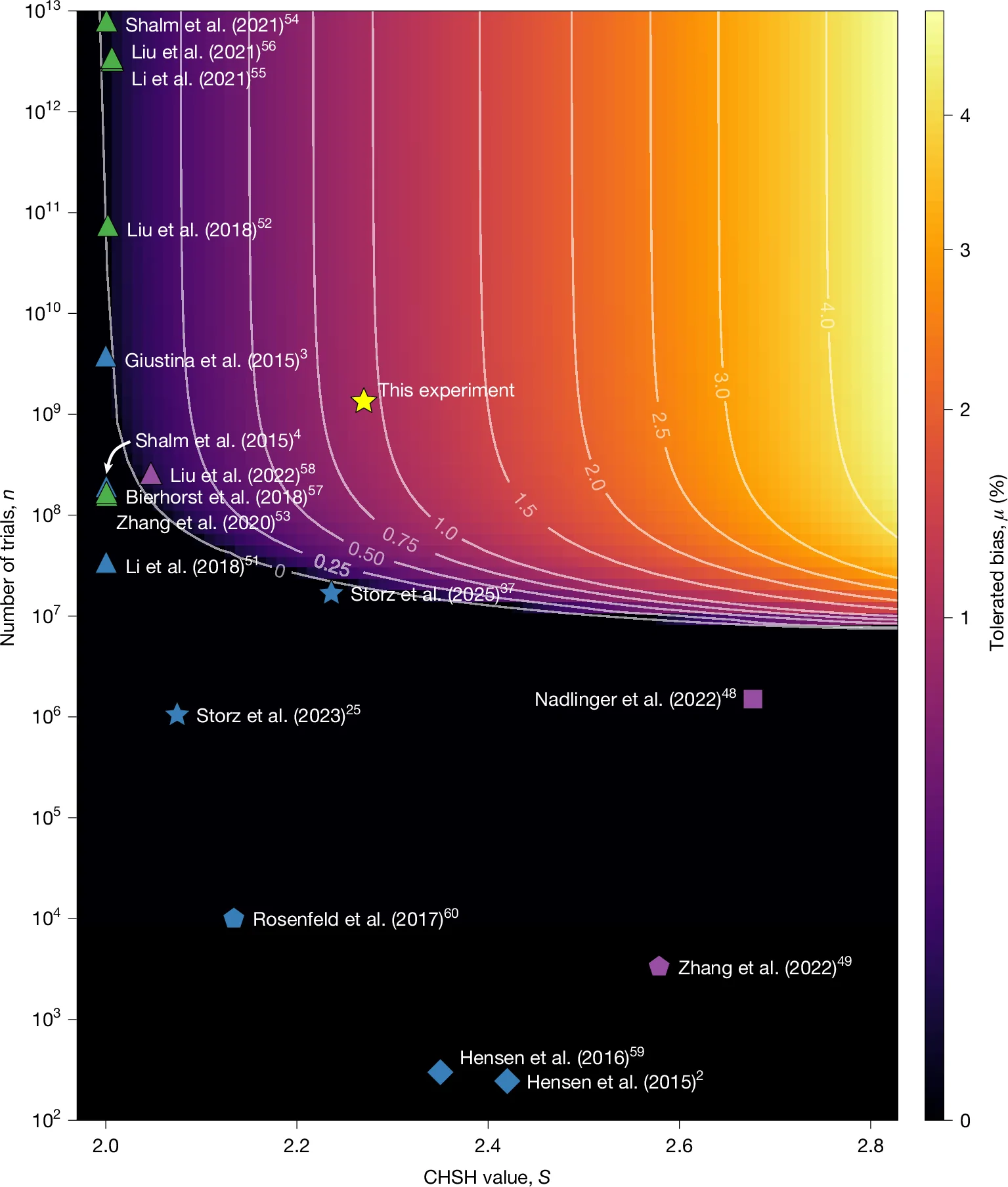
Comparative performance of a selection of Bell-test-based experiments for device-independent randomness amplification. The coloured region shows the trade-off between the CHSH value *S* and the number of trials *n* required to produce at least a million bits of uniform randomness, and a tolerated bias *μ*. We assume depolarizing noise and the use of a specific randomness extractor used in the present work (Methods). It is noted that this plot is subject to future theoretical advancements; for instance, improved extractors could allow to amplify sources with arbitrary bias *μ* < 50% (for details, see Supplementary Information section VIII). We further list previously published experiments. The marker shape represents the experimental platform: photons (triangles)^3,4,51–58^, nitrogen–vacancy centres (rhombi)^2,59^, neutral atoms (pentagons)^49,60^, trapped ions (square)^48^ and superconducting circuits (stars)^25,37^. The colour represents the type of experiment: green, device-independent randomness expansion; blue, loophole-free Bell test; purple, device-independent quantum key distribution; yellow, device-independent randomness amplification. The listed quantum key distribution experiments do not close the locality loophole.

The necessity of a loophole-free Bell test already poses great experimental challenges. But the demands for randomness amplification go even beyond this, requiring a substantial Bell violation together with a high repetition rate. We achieve them with an implementation on a solid-state circuit quantum electrodynamics platform^24^ using a set-up improved relative to the one recently used to demonstrate a loophole-free Bell test^25^. Improvements to the experimental hardware, including a reduction of the loss of the quantum channel used to initially entangle the superconducting qubits to below 14%, allowed us to attain a Clauser–Horne–Shimony–Holt (CHSH)^15^ value of *S*_obs_ = 2.271. Furthermore, achieving multi-day stability of room-temperature control electronics and calibration, as well as cutting down the classical communication and data processing overhead by two orders of magnitude, enabled us to collect a gigabit-range amount of data. These advances allowed us to reach a previously unattained regime in which randomness amplification is experimentally possible (Fig. 2).

## Amplifying randomness

To analyse randomness amplification, we need a quantitative measure of imperfection. Any source of random bits *B*_1_ … *B*_*n*_ can be characterized by a pair of parameters (*μ*, *ε*). Like any realistic device, the source may fail with some probability *ε*, in which case nothing is guaranteed about the randomness of *B*_1_ … *B*_*n*_. This failure probability *ε* is usually negligibly small but non-zero. The parameter *μ* quantifies the bias of each bit *B*_*i*_ of the source when it operates correctly, that is, conditioned on non-failure. To formalize this notion of bias *μ*, we adopt the Santha–Vazirani (SV) model^5^, considering an adversary attempting to predict the output bit *B*_*i*_ of the source. We say the source is a *μ*-SV source if ${P}_{\mathrm{guess}}({B}_{i}| E)\le \frac{1}{2}+\mu$ holds for all *i*. Here *E* denotes any (potentially quantum) side information that is available before the bit *B*_*i*_ is produced (this includes the prior bits *B*_1_ … *B*_*i*−1_) and *P*_guess_(*B*_*i*_∣*E*) denotes the probability of guessing *B*_*i*_, given access to *E* (for precise definitions, see Supplementary Information section VI). The case *μ* = 0 corresponds to a perfectly random (unbiased) source, while larger values of *μ* reflect increased predictability of the output.

Because the bias *μ* quantifies the predictive power of a potential adversary, it is not an experimentally measurable quantity. Nonetheless, the assumption that a source is (*μ*, *ε*)-random would be falsified by a statistical test that yields an observed bias *μ*_obs_ larger than *μ*. Therefore, if *μ*_obs_ satisfies *μ*_obs_ < *μ*, we say that the test result is compatible with the (*μ*, *ε*)-randomness assumption.

**Fig. 3:**
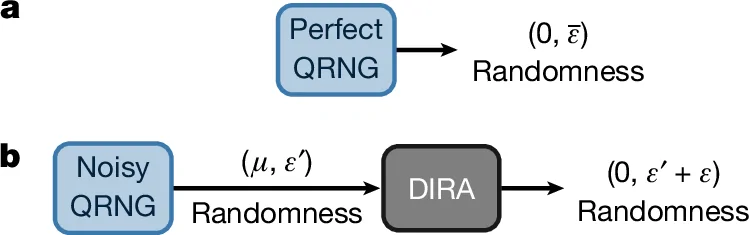
Comparison of traditional quantum random number generation with device-independent randomness amplification. **a**, For quantum random number generation (QRNG), one assumes that the devices are trusted (indicated by the blue box) and built such that they produce $(0,\bar{\varepsilon })$-randomness. **b**, By contrast, in device-independent randomness amplification (DIRA), one only requires access to a trusted but noisy QRNG producing (*μ*, *ε*)-random bits to generate the final (0,* ε* + *ε*′)-random bitstring. The DIRA implementation does not need to be trusted (indicated by the black box). Hence, the DIRA protocol allows us to produce uniform randomness using only a (*μ*, *ε*)-random source, a task that is impossible by classical means alone.

A randomness amplification protocol aims to reduce the bias while itself having a (small) failure probability. Formally, the protocol maps $(\mu ,{\varepsilon }^{{\prime} })\mapsto ({\mu }^{{\prime} },{\varepsilon }^{{\prime} }+\varepsilon )$ with *μ*′ < *μ*, where *ε* is the protocol’s own failure probability. Classically, this task is impossible: any classical processing of a *μ*-SV source can only produce an output with *μ*′ ≥ *μ* (ref. ^5^). Quantum protocols, however, can overcome this limitation. In this work, we demonstrate that, for any input source with *μ* ≤ 0.75%, our experimental set-up based on the protocol in Box 1 yields completely unbiased output randomness (*μ*′ = 0), that is, it achieves the transition $(\mu ,{\varepsilon }^{{\prime} })\mapsto (0,{\varepsilon }^{{\prime} }+\varepsilon )$, for any 0 < *ε*′ ≤ 1 (compare Fig. 3). The residual increase of the failure probability, *ε*, can in principle be made arbitrarily small at the cost of consuming more input randomness; for concreteness, we take *ε* = 10^−12^ (see ref. ^26^ and Methods for a detailed technical definition and operational interpretation). To achieve this, we use two spatially separated sources of randomness in our implementation (Fig. 1). For our analysis, we treat their concatenated outputs (in the order in which they were produced) as a single *μ*-SV source. Applying the SV criterion to this combined string permits arbitrary correlations between the two physical sources.

The core theoretical contribution of this paper lies in certifying the uniformity of the output bitstring from the observed inputs **XY** and outputs **AB**. This is made possible through the deployment of state-of-the-art theoretical methods, as elaborated in Methods and Supplementary Information section VI. The theoretical argument leading to this conclusion is based on the violation of a *μ*-dependent Bell inequality, called a measurement-dependent locality (MDL) inequality^27^ (Methods). The *μ*-MDL inequality asserts that observing the violation *S*_*μ*,obs_ > 0 (see Methods for a definition of *S*_*μ*,obs_) certifies the unpredictability of the measurement outcomes, even when the inputs are not uniformly random^27^. Although it relies on the same principle as Bell’s original argument for the unpredictability of quantum measurements^14^, the *μ*-MDL inequality does not require uniform input randomness. Otherwise, we adopt the standard assumptions of a loophole-free Bell test. The main improvement of our analysis over previous works, such as ref. ^20^, is that we allow for quantum side information about the SV source. This is achieved using the recently developed tool of two-process extractors^28^ (Methods and Supplementary Information section VI for details).

Box 1 Protocol (abstract) The protocol is defined in terms of the following parameters:

$n\in {\mathbb{N}}$: number of protocol trials

*μ*: bias of the two *μ*-SV sources

$m\in {\mathbb{N}}$: length of the final random bitstring

Ext: {0, 1}^2*n*^ × {0, 1}^*d*^ → {0, 1}^*m*^

the two-process randomness extractor.

(1) **Measurement:** For each trial *i* ∈ {1, 2, …, *n*}:

(a) Sample *x*_*i*_, *y*_*i*_ ∈ {0, 1} from the two *μ*-SV sources

(b) Feed the devices with the inputs *x*_*i*_, *y*_*i*_ and record the respective outputs *a*_*i*_, *b*_*i*_ ∈ {0, 1}.

(2) **Parameter estimation:** Use the inputs **X**, **Y** and the outputs **A**, **B** to evaluate the observed value of the *μ*-MDL inequality *S*_*μ*,obs_. If *S*_*μ*,obs_ ≤ 0, the protocol aborts and no random bits can be produced.

(3) **Randomness extraction:**

(a) Draw another bitstring **Z** ∈ {0, 1}^*d*^ from one of the sources.

(b) Apply the extractor to produce the final output **K** = Ext(**AB**, **Z**) of length *m*.

## Experimental implementation

We obtain the initial imperfect random bits from the quantum random number generators described in ref. ^29^, which generate on-demand randomness via laser phase diffusion. The quality of this source has been extensively tested (Supplementary Information section I). The results of these tests are compatible with an unpredictability bias *μ* below 0.75%.

To implement the randomness amplification protocol, we use the following experimental system: a two-node untrusted device *λ*, whose core component is realized by two dilution refrigerators connected via a modular cryogenic link spanning a distance of 30 m (ref. ^25^). We encode quantum information in two transmon qubits^30^ fabricated on separate, nominally identical quantum devices, operated in the cryostats at sites A and B at temperatures of about 15 mK. In addition to standard control and readout circuitry, each qubit is coupled to an on-chip transfer resonator and a Purcell filter^31^. For the generation of remote entanglement, we connect the transfer resonator circuits of the devices at both sites through a rectangular aluminium waveguide^32,33^. We also include in the untrusted device *λ* room-temperature electronics used to control quantum devices. The two-node device *λ* is further described in Supplementary Information section I. Single-shot readout of the state of the quantum devices generates the protocol output strings **A** and **B** (ref. ^34^).

**Fig. 4:**
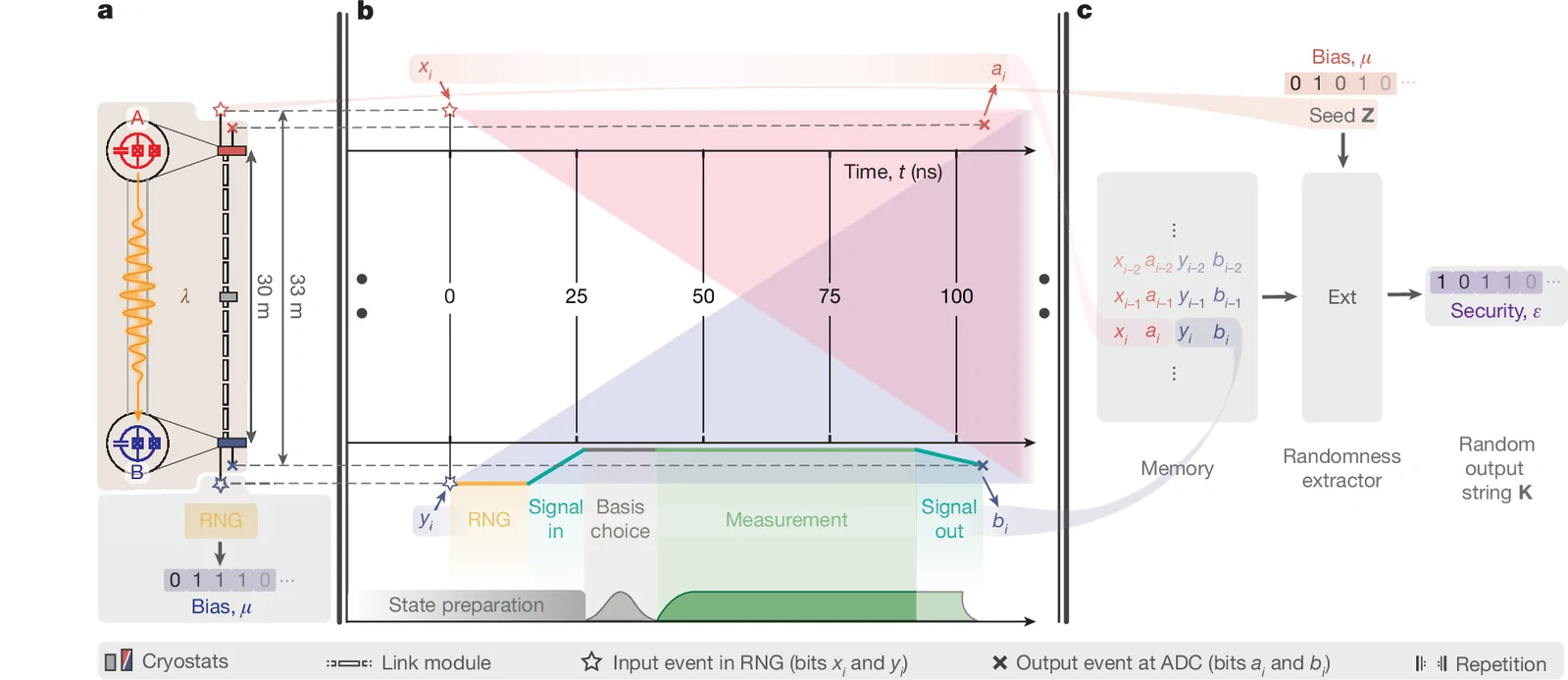
Full experimental protocol. **a**, Experimental set-up. Two dilution cryostats, separated by 30 m, contain a superconducting qubit each, forming the nodes A and B. In combination, they represent the untrusted device *λ*. Stars mark the location of the input quantum random number generators and crosses mark signal acquisition analogue-to-digital converters (ADCs). The distance between the starting event and the end of the acquisition is therefore 33 m. The inset on the bottom-left corner highlights one of the two random number generators (RNGs), producing a string of random bits with bias *μ*. **b**, Spacetime diagram of an experimental trial *i*. Each trial starts from sampling a random bit (*x*_*i*_, (red) for Alice, and *y*_*i*_, (blue) for Bob) from the quantum random number generators and ends with a discretized measurement result (*a*_*i*_, *b*_*i*_). Individual experimental segments are indicated below by different colours: the random number production time, the duration of the basis choice pulse and the measurement, and the duration of the signals entering and leaving the cryostats. **c**, Schematic representation of the post-processing routine, yielding a certified random string with security *ε* (see text for details).

Our experimental implementation is shown in Fig. 4 and follows the abstract interface in Box 1. In each trial *i*, we first perform active all-microwave reset to bring both qubits into the ground state^35^. We then prepare an entangled $| {\varPsi }^{+}\rangle$-Bell state using a deterministic remote entanglement generation scheme^31,36^, based on the transfer of a single microwave photon between the remotely connected qubits. Sampling a random input bit *x*_*i*_ (*y*_*i*_) at node A (B) from the *μ*-SV source on each site marks the beginning of the time-critical part of the randomness amplification protocol. At each node, as in refs. ^25,37^, the random input bit controls the state of a microwave switch that conditionally passes a microwave pulse to rotate the qubit by π/2 around a controlled axis, implementing the measurement basis selection. Next, we measure the state of the local qubit by sending a microwave pulse to the quantum device, interacting with the transmon qubit^34^. We then amplify the readout signal and route it out of the cryostat to digitize it at room temperature using an analogue-to-digital converter and post-process it using a field-programmable gate array. With this scheme, we achieve single-shot readout fidelity of about 98% in only 50 ns for both qubits. We consider the measurement result to be fixed once the trailing edge of the readout signal reaches the input of the analogue-to-digital converter, using similar considerations as discussed in ref. ^25^. Taking into account the data of each trial closes the fair-sampling loophole^38,39^, and not assuming that the individual trials are independent and identically distributed closes the memory loophole^40^.

**Fig. 5:**
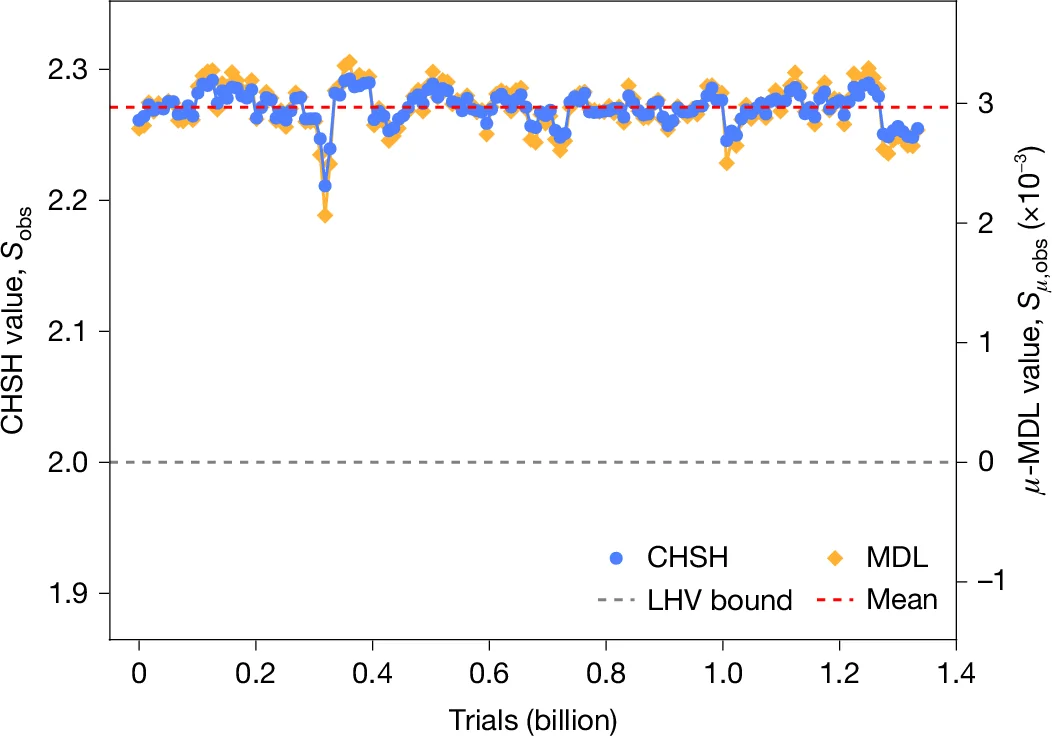
Bell violation versus number of trials. Each point corresponds to about 4 million (2^22^) experimental trials, for which the CHSH and *μ*-MDL values were evaluated. Here we use a value of *μ* = 0.75%. The *μ*-MDL value *S*_*μ*,obs_ quantifies the strength of the correlations between the two nodes of the device (Supplementary Information section VI). A positive *μ*-MDL value certifies that the device produces unpredictable bits. The *μ*-MDL inequality can be thought of as a Bell inequality that does not require the assumption of perfectly random input bits. For reference, we also plot the CHSH value *S*_obs_. It is noted that this value is not relevant for the randomness amplification protocol and is only shown for comparison with previous experiments. The local hidden variable (LHV) bound *S*_LHV_ = 2 (*S*_*μ*,LHV_ = 0) is shown in grey. The mean of the observed *μ*-MDL and CHSH values are *S*_*μ*,obs_ = 0.00296 and *S*_obs_ = 2.271, respectively.

We perform randomness amplification based on *n* = 20 × 2^26^ = 1,342,177,280 trials acquired over a total time of about 9 hours. We split the data acquisition in 20 blocks of 2^26^ (about 67 million) trials each. Trials in each block are run back-to-back with a repetition time of 20 μs, that is, a rate of 50 kHz. The evolution of the *μ*-MDL and CHSH inequality violations throughout the duration of the experiment is shown in Fig. 5. We measure an average CHSH value of *S*_obs_ = 2.271 and an average *μ*-MDL value of *S*_*μ*,obs_ = 0.00296. Both quantities show minor fluctuations around a constant mean throughout the duration of the experiment, remaining well above the classical threshold.

After acquiring a data block, we re-calibrate the readout integration weights^34^ and perform a verification of the system’s timings. Furthermore, as closing the locality loophole is of paramount importance for the device-independent nature of the protocol, we implement a real-time monitoring of the set-up synchronization during the protocol execution. Specifically, we introduce a series of oscilloscope measurements observing the arrival time of auxiliary pulses from the various devices in the set-up, hence ensuring the absence of timing jitter of control pulses, triggers and readout signals. In Supplementary Information sections II and III, we discuss the triggering and timing verification schemes and the system-wide timing stability data during the protocol execution.

## Results and discussion

As a final result of the randomness amplification protocol, we generate a random bitstring **K** consisting of *m* = 45,025,658 bits, starting from 5,368,709,120 low-quality random bits. The length of the output bitstring was chosen such that a failure probability of the protocol as low as *ε* = 10^−12^ is guaranteed (see Supplementary Information section VI for details). The randomness of the bitstring is guaranteed to be perfect provided that the bias *μ* of the input source is below 0.75%.

Although it is fundamentally impossible to verify the unpredictability of a bitstring by analysing the string itself, we may still run statistical tests on it as a consistency check. Concretely, we run the National Institute of Standards and Technology (NIST) statistical test suite^41^ and the Diehard^42^ batteries of statistical tests on the string **K** generated by the full execution of our randomness amplification scheme. We find that the result passes all the tests for which the string is sufficiently long.

The amplified randomness may be used directly as a public source of randomness, comparable to the NIST randomness beacon^43^ or the one presented in ref. ^44^. The advantage of the randomness amplification scheme compared with existing methods is that no perfect input randomness is required and the output bits come with a certificate (the data showing the Bell violation), which proves that they have full entropy, even conditioned on the low-quality input bits. It is noted that the bit rate of our scheme is comparable to that of the current NIST randomness beacon.

In the future, when multi-node networks with high-fidelity communication links are available, randomness amplification may be combined with distributed computing protocols^45,46^. This would reduce the assumptions on the classical processing, thereby guaranteeing high-quality output randomness even if a certain number of network nodes are corrupted.

High-quality randomness is necessary to guarantee the security of cryptographic schemes. For example, the claim that quantum key distribution is secure relies on the assumption that the random bits that are used in the protocol, such as for choosing the measurement bases, are unpredictable. Furthermore, cryptographic tasks such as bit commitment, encryption, secret sharing, zero-knowledge proofs and secure multi-party computation cannot be implemented securely with sources of less-than-perfect randomness^47^. Owing to the high repetition rate of our experiment, the amount of randomness produced in this work exceeds the randomness consumed by the recently published device-independent quantum key distribution implementations^48,49^. The combination of the two schemes therefore offers secure communication under minimal assumptions^50^, even if no perfect randomness is available.

## Methods

### Definition of randomness

The definition of randomness used in this work follows refs. ^1^^,61^. Here, the notion of a spacetime variable is introduced. This is a random variable with an associated coordinate that indicates the physical location of the value in relativistic spacetime. The output *X* of a random process as well as any side information is modelled by such spacetime variables. Let *X* denote the spacetime variable whose coordinate indicates the point in spacetime where the process of generating *X* is started, also called the trigger event. *X* is called *ε*-random if it is *ε*-close to uniform and uncorrelated to all other spacetime variables that are outside of the future light cone of *X*. Denoting this set by *Γ*_*X*_, this can be expressed as

1 $$\frac{1}{2}\parallel {P}_{X{\varGamma }_{X}}-{U}_{X}\times {P}_{{\varGamma }_{X}}{\parallel }_{1}\le \varepsilon ,$$

where ${P}_{X{\varGamma }_{X}}$ is the joint probability distribution over the random variables *X* and *Γ*_*X*_, *U*_*X*_ is the uniform distribution on *X*, that is, ${U}_{X}(x)=\frac{1}{| {\mathcal{X}}| }$ for all $x\in {\mathcal{X}}$ (${\mathcal{X}}$ is the alphabet of *X*), and

2 $$\frac{1}{2}\parallel {P}_{X}-{Q}_{X}{\parallel }_{1}=\frac{1}{2}\sum _{x}| {P}_{X}(x)-{Q}_{X}(x)| $$

is the trace distance between two probability distributions. In addition, the process generating *X* may fail and not produce any randomness. Therefore, the ideal output distribution is of the form

3 $${P}_{X{\varGamma }_{X}}^{\mathrm{ideal}}=\Pr [\varOmega ]{U}_{X}\times {P}_{{\varGamma }_{X}| \varOmega }+\Pr [\bar{\varOmega }]{\delta }_{X}^{\perp }\times {P}_{{\varGamma }_{X}| \bar{\varOmega }},$$

where *Ω* denotes the event of the process succeeding, $\bar{\varOmega }$ denotes the complementary event of the process failing, ${\delta }_{X}^{\perp }$ is the distribution, which deterministically produces a bitstring of length zero, and ${P}_{X{\varGamma }_{X}| \varOmega }$ (${P}_{X{\varGamma }_{X}| \bar{\varOmega }}$) denotes the distribution conditioned on *Ω* ($\bar{\varOmega }$). Hence, for *X* to be called *ε*-random, the distribution ${P}_{X{\varGamma }_{X}}$ must be close to this ideal distribution, that is, it must satisfy

4 $$\frac{1}{2}\parallel {P}_{X{\varGamma }_{X}}-{P}_{X{\varGamma }_{X}}^{\mathrm{ideal}}{\parallel }_{1}=\Pr [\varOmega ]\frac{1}{2}\parallel {P}_{X{\varGamma }_{X}| \varOmega }-{U}_{X}\times {P}_{{\varGamma }_{X}| \varOmega }{\parallel }_{1}\le \varepsilon ,$$

where we noted that the term with $\bar{\varOmega }$ disappears as ${P}_{X{\varGamma }_{X}| \bar{{\Omega }}}={P}_{X{\varGamma }_{X}| \bar{\varOmega }}^{\mathrm{ideal}}\,=$ ${\delta }_{X}^{\perp }\times {P}_{{\varGamma }_{X}| \bar{\varOmega }}$.

The independence condition in equation (4) can be lifted to a condition on quantum states

5 $$\Pr [\varOmega ]\frac{1}{2}\parallel {\rho }_{XE| \varOmega }-{\omega }_{X}\otimes {\rho }_{E| \varOmega }{\parallel }_{1}\le \varepsilon ,$$

where *E* is any quantum system outside of a future light cone of *X*, $\parallel \cdot {\parallel }_{1}=\mathrm{tr}(| \cdot | )$ denotes the trace norm, and ${\omega }_{X}=\frac{1}{| {\mathcal{X}}| }{\mathrm{id}}_{X}$ is the maximally mixed state on *X*. Importantly, the trace norm can only decrease when we apply a completely positive trace-preserving map (for example, a measurement) on the system *E*. Hence, the independence condition in equation (4) follows from the quantum version in equation (5).

The condition in equation (5) ensures that the random variable *X* is uncorrelated with anything at an earlier time, except with probability *ε* (refs. ^62,63^). Thus, in line with what was mentioned in the main text, the value of *X* is unpredictable, guaranteed by the laws of physics.

### Loopholes and assumptions in experimental implementations

As device-independent randomness amplification aims to certify the output of a device solely based on fundamental physics principles, it is critical to ensure that an experimental implementation makes no additional assumptions about the set-up. If such assumptions are present, they open so-called loopholes in the conclusions drawn from the experiments^40^, similarly to the situation with Bell tests. Specifically, the locality loophole^64^ opens when the two nodes A and B are able to exchange classical information throughout each trial of the experiment. This would allow them to adapt the measurement result on one node based on the measurement basis choice on the other node, and thereby to achieve strong correlations in the input–output-bit statistics that are however not necessarily non-local. This issue can be addressed by setting up the experiment in a spacetime configuration, where the two nodes are space-like separated throughout each trial of the protocol. This ensures that in each trial, no signal travelling at most at the speed of light, possibly carrying information about the measurement basis of one node, can reach the other node before the latter has completed its measurement. Therefore, carefully determining the spacetime configuration of the start and stop events of each trial ensures that the locality loophole is closed. Sometimes in device-independent protocols, the locality loophole is not explicitly closed but replaced with a shielding assumption—namely, that the devices do not leak any unwanted information to the outside world. Once this shielding assumption is made, it effectively provides everything that would otherwise be guaranteed by enforcing locality. This is in particular common for device-independent quantum key distribution, where it is essential that the generated key remains secret. By contrast, device-independent randomness amplification can also be meaningful in scenarios where the generated randomness does not need to remain secret (for instance, in a public lottery). In such cases, the shielding assumption is no longer necessary, and the weaker locality assumption becomes the relevant one.

Furthermore, the fair-sampling loophole^38,39^ opens if assumptions are made about the distribution of the underlying input–output-bit correlations, for instance, because one does not have access to the measurement results of all trials. This loophole is closed by including the data captured in all trials of the experiment. Third, the memory loophole^40^ is addressed by not assuming that the results of each trial are independent and identically distributed.

A Bell test closing all these loopholes simultaneously is typically referred to as loophole-free, and protocols based on Bell tests that achieve the same feat are termed device-independent, as they do not rely on assumptions about the employed devices.

### Entropy production and MDL inequality

The security of our device-independent randomness amplification protocol hinges on the property that the measurement results of Bell tests exhibit a certain degree of inherent randomness^65–68^. For most Bell inequalities, including the widely known CHSH inequality, this property only holds under the assumption that the inputs are chosen uniformly at random. This assumption is clearly not met in a device-independent randomness amplification experiment, where the goal is precisely to process imperfect randomness into better randomness. Therefore, what we require for device-independent randomness amplification is a special type of Bell inequality that can be used to certify the randomness of the devices’ outputs even when the inputs are chosen using an SV source. This is exactly what is achieved by the following Bell inequality (also called an MDL inequality)^27^:

6 $$\begin{array}{l}0\ge {S}_{\mu }\equiv {\mu }_{\min }\times {P}_{ABXY}(0000)-{\mu }_{\max }\times \\ \,\,\,\times ({P}_{ABXY}(0101)+{P}_{ABXY}(1010)+{P}_{ABXY}(0011)),\end{array}$$

where *μ*_min_ (*μ*_max_) are lower (upper) bounds on the probability of the source to produce any given output *xy*. By observing *S*_*μ*,obs_ > 0, one can then certify that there is no hidden variable that would allow the adversary to predict the outputs *A* and *B* perfectly^27^.

In our experiment, we assume that both sources are *μ*-SV sources and therefore we have *μ*_min_ = (*μ* − 0.5)^2^, *μ*_max_ = (*μ* + 0.5)^2^. To emphasize the dependence of the MDL inequality (6) on the tolerated bias *μ*, we also call it a *μ*-MDL inequality. In the special case of a perfect source (that is *μ* = 0), the above inequality corresponds to the well-known CHSH inequality (in Eberhard form^39^). It is noted that the inequality (6) becomes increasingly difficult to violate as *μ* increases. Therefore, for any quality of the experiment, there exists a maximal threshold bias *μ* of the sources that can be tolerated (compare Fig. 2).

More quantitatively, we are interested in the amount of randomness that can be extracted from the outputs **AB** of our device. It is well established that the number of extractable random bits is characterized by the conditional smooth min-entropy ${H}_{\min }^{{\varepsilon }_{{\rm{s}}}}({\bf{AB}}| {E}_{\mathrm{fin}})$ (the min-entropy corresponds to the negative logarithm of the guessing probability: ${H}_{\min }(A| B)=-{\log }_{2}{P}_{\mathrm{guess}}(A| B)$ and the smoothing parameter 0 < *ε*_s_ < *ε* is a freely chosen fraction of the overall failure probability *ε*; for a formal definition, see Supplementary Table III), where *E*_fin_ denotes any side information the adversary may hold about **AB** at the end of the protocol^28,69–71^. In our protocol, we include the outputs of the source **XY** as part of the *E*_fin_ system. This means that our protocol remains secure even if the adversary learns the outputs of the SV source as they are produced. This property is called privatization. The main task now is to find a bound on the smooth min-entropy. This is a challenging problem in general as the dimension of **AB** is very large. To overcome this difficulty, we need a method to bound ${H}_{\min }^{{\varepsilon }_{{\rm{s}}}}({\bf{AB}}| {E}_{\mathrm{fin}})$ in terms of a simpler quantity that we can evaluate. This is exactly what is achieved by the entropy accumulation theorem^72^. In rough terms, the entropy accumulation theorem states that^72,73^

7 $${H}_{\min }^{{\varepsilon }_{{\rm{s}}}}({\bf{AB}}| {E}_{\mathrm{fin}})\ge H({S}_{\mu })n-c\sqrt{n},$$

where *H*(*S*_*μ*_) is the single-round von Neumann entropy as a function of the *μ*-MDL violation *S*_*μ*_ and *c* is a constant. Hence, the entropy accumulation theorem guarantees that there is randomness in **AB** whenever *S*_*μ*,obs_ > 0.

### Randomness extraction

Even though the outputs **AB** of the Bell-type experiment possess an inherent amount of randomness (quantified by ${H}_{\min }^{{\varepsilon }_{{\rm{s}}}}$), they may be strongly correlated with each other or with side information held by an adversary. Consequently, they do not satisfy the definition of *ε*-randomness given in equation (5) which, loosely speaking, requires the outputs to be distinguishable from perfectly uniform with probability at most *ε*. To break these correlations, we apply a randomness extractor that processes two sources of imperfect randomness into a new string of nearly perfect randomness (the function ‘Ext’ in Fig. 1).

For randomness extraction to work, one requires two (or more) independent sources of randomness. More precisely, we use a recently developed kind of randomness extractor known as two-process extractors^28^. A two-process extractor allows for the extraction of randomness from two strings of random bits if the bitstrings were produced by independent processes and both processes produce a sufficient amount of entropy (for a formal introduction, see Supplementary Information section VI). In our set-up, the first bitstring is the joint output **AB** of the Bell test and the second bitstring **Z** is drawn from one of the two *μ*-SV sources (Fig. 1). The randomness of the bitstring **AB** is guaranteed by equation (7), whereas the randomness of **Z** is guaranteed by the condition that it was produced by a *μ*-SV source. As both entropies decrease with increasing bias *μ*, there is a maximal value of *μ* ≈ 4% that can be tolerated (Fig. 2). In addition, the required independence assumption is satisfied because the *μ*-SV source is isolated from the Bell test (see Supplementary Information section V for a summary of our assumptions) and hence **AB** and **Z** are produced by independent processes (although the bitstrings **AB** and **Z** can still be correlated). Hence, we can apply our two-process extractor to produce the final bitstring **K** (see Supplementary Information section VI for the full security proof).

Finally, we highlight the major difference between our security analysis and previous works on device-independent randomness amplification (DIRA), such as ref. ^20^. In ref. ^20^, the authors consider only classical side information about the *μ*-SV source, which allows them to argue that the bitstrings **AB** and **Z** are conditionally independent. Hence, they can apply the two-source extractors from ref. ^71^. However, as we consider more general adversaries holding quantum side information about the source, we no longer have (conditional) independence between **AB** and **Z**. This makes it necessary to use the more general two-process extractors.

### Set-up improvements

In this experiment, we aim to generate a megabit-range output bitstring, which requires to perform on the order of 10^9^ Bell test trials. This number exceeds by 3 orders of magnitude the statistics presented in ref. ^25^, which demonstrated a loophole-free Bell inequality violation with superconducting circuits based on 10^6^ trials. Such an increase of the amount of data required improvements to the performance and stability of the set-up. We have approached this challenge from two directions by shortening the required data acquisition time and engineering the control electronics to be stable over longer timescales. The former was achieved by increasing the repetition rate of the experiment from 12.5 kHz to 50 kHz, as enabled by improving the impedance matching of room-temperature passive microwave components previously showing non-trivial long-time behaviour limiting the repetition time. This improved the required pure experimental time to collect the reported dataset from over 31 hours to below 8 hours. Using the field-programmable-gate-array-based qubit readout and state classification, in this experiment we transferred only two bits of data per trial from the measurement device (a field-programmable gate array) to the PC controlling the experiment and processing the data. Bypassing the standard Python-based data handling and keeping the data processing to the binary format using faster compiled C++, together with the minimal amount of networked data, greatly decreased the digital processing and network communication overhead time from >200 μs per trial to <2μs per trial, putting the total digital processing and network communication overhead time from over 75 hours to below 45 minutes. These improvements have shortened the required data acquisition time to slightly less than 9 hours. We have also majorly enhanced the stability of the room-temperature microwave control electronics and the steadiness of the pulse timing and triggering, which resulted in the absence of jitter on the scale of several days, and performance stability illustrated by Fig. 5. Technical details of the modifications and our timing and triggering schemes are discussed in Supplementary Information section II.

For the presented data, we also improved the CHSH S-value from *S* = 2.07 in ref. ^25^ to *S* = 2.27. In addition to an increase stemming from the enhanced stability of control electronics and triggering, we have targeted the main factor limiting the fidelity of the Bell state, that is, microwave photon losses in the quantum channel connecting the two sites. As the 30-m superconducting aluminium waveguide connecting the nodes has losses below <1 dB km^−1^, the majority of losses are due to impedance mismatches and resistive losses of the on-chip and printed circuit board (PCB) wiring, semi-rigid coaxial cables connecting the sample mount to the waveguide, and coax-to-waveguide and coax-to-PCB adapters. We have redesigned and improved the PCB-to-waveguide cabling by decreasing the number of adapter parts, and by replacing normal-metal semi-rigid coaxial cables by superconducting ones^74^. These changes have cut the channel loss by about three to four percentage points. Furthermore, in the previous experiments, we have used a circulator in the communication line with the twofold aim: to aid with thermalization of the intra-waveguide stray radiation, and to allow measurement of the temporal mode function of microwave photons emitted from either of the nodes^31^. From a separate cryogenic transmission measurement, we estimate the photon loss due to the circulator to be about 2–4%. In this work, we removed the circulator, therefore omitting those losses. In total, we estimate to have decreased the channel loss from about 19% to about 12–14%.

## Online content

Any methods, additional references, Nature Portfolio reporting summaries, source data, extended data, supplementary information, acknowledgements, peer review information; details of author contributions and competing interests; and statements of data and code availability are available at https://doi.org/10.1038/s41586-026-10521-8.

## Supplementary information

Supplementary Information Supplementary Information, including Supplementary Figs. 1–10, Tables 1–4 and References.

Peer Review File

## Data Availability

The data that support the findings of this study have been deposited in the ETH Zurich Research Collection and are publicly available at https://doi.org/10.3929/ethz-c-000797430.

## Code availability

The code used for randomness extraction and data analysis has been deposited in the ETH Zurich Research Collection and is publicly available at https://doi.org/10.3929/ethz-c-000797430.
