# Supplementary material for: Experimental randomness amplification
Source: Nature. 2026 May 27;653(8116):1033–8. doi: 10.1038/s41586-026-10521-8 (PMC13658030; doi:10.1038/s41586-026-10521-8)
Supplement: Supplementary file 1 [file 41586_2026_10521_MOESM1_ESM.pdf]

nature portfolio
https://doi.org/10.1038/s41586-026-10521-8

Supplementary information

Experimental randomness amplification

In the format provided by the
authors and unedited

Supplementary Information
Experimental Randomness Amplification

Anatoly Kulikov,[1,2,*] Simon Storz,[1,2] Josua D. Schär,[1,2] Martin Sandfuchs,[1,2] Ramona Wolf,[1,2,3,4] Florence Berterottière,[1,2] Christoph Hellings,[1,2] Andreas Wallraff,[1,2] and Renato Renner[1,2]

[1]*Department of Physics, ETH Zurich, 8093 Zurich, Switzerland*
[2]*Quantum Center, ETH Zurich, 8093 Zurich, Switzerland*
[3]*Naturwissenschaftlich-Technische Fakultät, Universität Siegen, 57068 Siegen, Germany*
[4]*Universität Innsbruck, Institut für Theoretische Physik, Technikerstraße 21a, 6020 Innsbruck, Austria*

(Dated: April 8, 2026)

I. EXPERIMENTAL DEVICES

In this section, we describe the experimental equipment used in the device-independent randomness amplification implementation and specifically focus on what forms part of the untrusted device λ introduced in the main text, and which devices we consider to be trusted. Brief schematics of individual components of the experimental setup and the division into an untrusted and trusted parts are illustrated in Fig. S1.

First and foremost, λ contains the cryogenic system which operates two remotely connected superconducting circuit quantum devices. As discussed in detail in Ref. [1], the system is composed of two dilution refrigerators at nodes A and B, housing the quantum devices, of a central cooling unit, and of a 30-meter-long cryogenic quantum link. The quantum link contains a rectangular Aluminium waveguide cooled to temperatures below 50 mK along the full distance, serving as a quantum channel between the two nodes.

The quantum devices operated at the nodes each contain a transmon-style qubit, coupled to control lines for performing gates along X, Y and Z axes of the corresponding Bloch sphere (σ_x, σ_y and σ_z operators), and a resonator-Purcell filter circuit used for reading out the state of the qubit using a microwave tone [2]. The qubits are furthermore coupled to another resonator-Purcell filter circuit which connects them to the 30-meter-long quantum channel. This connection allows for the exchange of single microwave photons between the two nodes, and thereby for the generation of entanglement [3]. The qubits are operated at a frequency of about 7.8 GHz, and they show coherence times of about $T_1 \sim$20 μs and $T_2^\star \sim$10 μs. We refer the interested reader to Ref. [4], which uses the same quantum devices, for further information.

In addition, we consider all of the electronic setup for controlling and reading out the state of the qubits as part of the untrusted device λ (with explicit exceptions mentioned below). This includes the arbitrary waveform generators and microwave generators used to prepare qubit control pulses and the readout signals, microwave equipment such as mixers and amplifiers, and the cables connecting the room-temperature electronics to the quantum devices in the cryostats. Hence the entirety of the quantum and cryogenic hardware, as well as the majority of the control electronics form part of the untrusted two-node black-box device λ.

The trusted part of the equipment, located outside of λ, is formed by the devices necessary to verify the device-independent protocol interface (see Box I in the main text) and ensure the closure of the locality loophole. First, the random number generators (RNGs) used to prepare the input bits are considered trusted, although imperfect, which reflects the assumption of them being Santha-Vazirani (SV) sources [5]. Second, regarding the measurement process, we consider trusted the analog-to-digital converter (ADC) used to digitize the measurement signal, and the field-programmable gate array (FPGA) used to perform state discrimination and post-processing. Also outside of λ is the equipment used to verify the space-time configuration of the experiment, relevant for closing the locality loophole. This includes the oscilloscopes used for the timing verification and to record reference pulses (see Fig. S1); further details about the real-time synchronization monitoring and the verification devices are given in Supplementary Information sections II and III. Finally, the computer storing the measurement results in a classical memory and performing the randomness extraction are part of the trusted equipment.

The fast optical RNGs we employ in this work were introduced in [6] and contain eight independent quantum entropy sources (QESs) each. Each QES consists of a laser diode that is periodically driven above and below the lasing threshold at a frequency of 400 MHz. During the off period, the intracavity field decays and becomes highly susceptible to phase diffusion; when the laser turns on again, it emits a pulse with a random optical phase uniformly distributed in $[0;2\pi)$. The pulse train passes through an unbalanced Mach–Zehnder interferometer, which converts phase randomness into intensity fluctuations detected by a photodiode. The resulting signal is digitized by a comparator with a 50/50 threshold, yielding one raw bit per pulse. Within each RNG, digital logic combines the eight raw bits from the QESs using XOR operations to produce a final bit that is less predictable than any single source.

The manufacturer characterizes the predictability of the generated bit X by its *excess predictability* ε, defined such that $P(X=0|\text{priors}) \in [\frac{1}{2}(1-\varepsilon), \frac{1}{2}(1+\varepsilon)]$. Based on the validated physical model described in [6], an upper bound of $\varepsilon < 5.24 \cdot 10^{-6}$ is established. This characterization is necessarily device-dependent and relies on assumptions about the internal workings of the hardware, yet it provides a conserva-

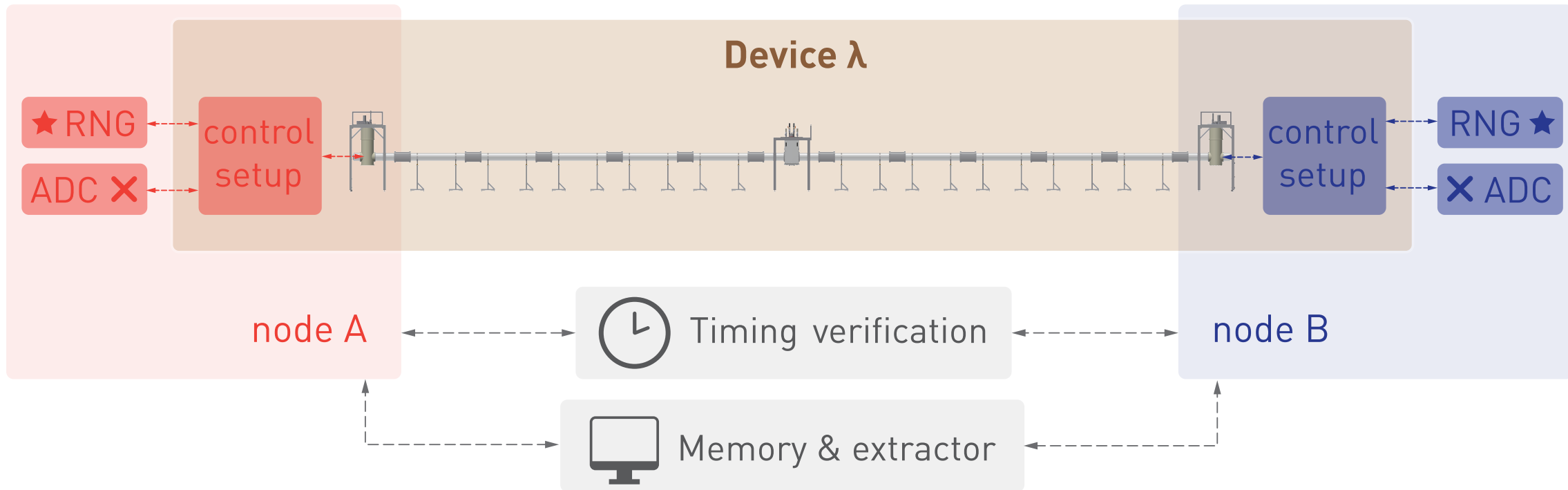

FIG. S1. Division of the experimental setup into the untrusted (Device λ) and trusted (everything outside of λ) parts. Red and blue squares highlight the locations of nodes A and B of the two-node setup, which are separated by 30 metres. The untrusted part contains the entirety of the cryogenic enclosure and quantum devices, the room-temperature (RT) control setup and wiring. The only two trusted components of the RT electronics are a randomness source and an ADC, compare with Fig. 1 in the main text. Timing verification equipment and classical memory also form part of the trusted equipment. Further details about timing verification are provided in Supplemental Information section II.

tive bound that is orders of magnitude smaller than the bias assumed in our analysis. As the manufacturer's definition of excess predictability and our definition of an SV source bias μ coincide, treating the input RNGs as Santha–Vazirani sources with $\mu \leq 0.75\%$ is a well-justified and highly conservative assumption.

II. TRIGGERING SCHEME, SETUP & STABILITY MODIFICATIONS

The requirement to maintain sub-nanosecond synchronization precision over timescales of several days poses a significant practical challenge. Furthermore, the hardware specifications and datasheets of the microwave devices used in the setup only guarantee jitter on the order of the individual devices' granularity, which for a Tektronix arbitrary waveform generator (AWG) AWG5014 comes to about 1.67 ns. Hence it has taken several iterations to achieve the reliable and stable synchronisation of the electronic devices used for the experiment, which shows effectively no jitter on week-long time windows. In the following, we discuss key aspects of our synchronisation scheme, including the distribution of a stable reference clock and trigger signals, the avoidance of jitter of electronic signals, and an automated timing verification scheme.

Reference clock and trigger – We generate the main reference clock signal at the center of the cryogenic link using a two-stage process. First, a Rubidium clock with an output frequency of 10 MHz is used as a reference input to a dedicated microwave generator (MWG). The generator then outputs a stable 1 GHz signal, which we distribute to both end nodes A and B through dedicated coaxial cables of identical length, as illustrated in Fig. S2. The symmetrical cabling scheme ensures an equal time delay and signal power at both nodes. Furthermore, the variations of electrical length of the cables due to global temperature variations in the laboratory are also identical. At the nodes, we daisy-chain the microwave generators involved in the experiment using the 1 GHz reference input/output port of the used microwave sources, where the first MWG in the chain is synchronized to the reference clock signal in the middle.

In the applications involving synchronizing a heterogeneous network of multiple digital and analog signal sources, one typically faces the problem of signal jitter, a property of digital electronics to have bi-stable input trigger regions. We mitigate this problem by clocking the local electronic devices using frequency, phase and power-optimized reference signals generated by dedicated MWGs, which are locked to the main reference clock. This also helps minimizing local phase drifts, as further discussed below.

We generate the master trigger signal using a Tektronix AWG5014 located at the center of the link and distribute it to the two nodes, similarly to the reference clock. At each node, an AWG5014 receives the main trigger signal for triggering the relevant local electronic devices.

Timing verification – To monitor the relevant timings in the experiment and verify the closure of the locality loophole during the experiment execution, we operate oscilloscopes at each node. We use them to record the arrival times of reference pulses generated by AWGs and the RNG, output at a predefined moment in time, and sent through cables of a known constant length, see Fig. S2a. The inset in Fig. S2a displays the typical pulse sequence of a trial of the experiment, including microwave pulses sent to the qubit in the cryostat and reference pulses for the oscilloscope. The local oscilloscope records the reference pulses from the individual devices repeatedly over the course of several seconds, and stores the envelope of the captured traces, i.e. the maximum and minimum voltage measured at each point in time throughout the current acquisition period (Fig. S2c). In the timing measurements presented in Supplementary Information sections III and IV, fol-

lowing a conservative approach, we use the envelopes which maximize the total protocol duration, therefore calculating an upper bound on the actual duration of each individual trial.

Phase drift measurements – Using the setup described above, we determined the phase drifts of the individual devices under the optimized clocking and triggering scheme, characterizing the duration of the absence of jitter in the system. The absolute drifts summarized in Table S1 are measured relative to the AWG5014 at the nodes A & B, we took the larger of the two measured values. The AWGs and FPGAs have small drift compared to their granularity. The largest drift belongs to the quantum random number generators (QRNGs), limiting the system to be in a jitter-free configuration for about 2.5 days. To make full use of a stable configuration for the main experiment presented in this publication, we adjust the phases of the reference signals clocking the AWGs in a way so that the trigger arrival time on all devices is in the middle of their two granularity points before starting acquiring the dataset.

Device	Granularity in ns	Relative drift in ps/hr
AWG5014 (other node)	1.66	0.1
FPGA	8	0.1
AWG70K	6.4	0.2
QRNG	2	16.9

TABLE S1. Granularity and drift properties of the electronic devices used to control or readout the qubit. The drift measurements are taken about a course of 50 hours and are a linear fit to the obtained data from the oscilloscope.

III. TIMING STABILITY

As the main experimental sequence is sufficiently extended in time (spanning more than 8 hours), in addition to full timing calibration before and timing verification after the experiment, we employ interleaved timing checks ensuring that the locality loophole is closed during the entire duration of the experiment. To achieve that, we install two oscilloscopes, one at each node of the setup, and connect them to the auxiliary outputs of the three room-temperature control devices at each node: the master device of the node (Tektronix AWG 5014), the device we use to generate the qubit control pulses (Tektronix AWG 70k), and the QRNG. Finally, we connect the fourth port of the oscilloscope via a splitter to a cable connecting the two nodes A and B in order to monitor the synchronisation between the nodes.

Before the first and after every block of data during the data acquisition (recall, each block contains 4^{13} ($\sim$ 67 million) trials) we perform the following timing verification routine: we program a reference pulse sequence (inset in Fig. S2a) to every device in the system, and acquire several thousand triggers over fifteen seconds on both oscilloscopes. We process the data by taking the envelope of received signals (a result of such data acquisition is shown in Fig. S2c), and for a certain threshold we plot the low and high envelope point. As we trigger a local oscilloscope on the signal arriving from the master AWG of the corresponding node, observing a signal arriving from another device allows monitoring relative time stability of the two devices. In addition, presence (or absence) of a time difference between the low and high envelopes of the same signal signifies presence (or absence) of trigger jitter – another effect which could prevent us from closing the locality loophole. Note, that time differences sufficiently below 1 ns are due to the pulse width and limited timing resolution of the measurement device and are not a sign of jitter: trigger or device jitter would lead to the time difference of at least the smallest granularity of the control devices, which is 1.66 ns in our case (Tektronix AWG 5014).

Fig. S3 shows the inter-node timing and its stability. The key information one obtains from the figure is the deviations between the maximum and minimum for each curve (see figure caption for details) are bounded by at most 200ps and the curves are stable in time. In a similar fashion, Fig. S4a demonstrates the relative time stability of the two AWGs on each node, and Fig. S4b shows the QRNG output timing stability. Note that, as QRNG does not output a pulse, but rather switches the state and stays in the new state, we could not measure the low envelope of the switching channel (and hence observe possible jitter in the positive time direction). An example of such measurement is also shown in Fig. S2c. In other words, if during one of the trials the QRNG switches later than it should, it would not appear on our measurement. That does not lead to opening the locality loophole however, as it only makes the length of the algorithm during that trial shorter (and decreases the state preparation quality, of course).

Evidently, all three figures show neither jitter nor instability in the timings during the experimental execution. From Fig. S3 one can remark that our two nodes are not perfectly in sync, but rather their time frames differ by 0.55 ns. This level of clocking mismatch is reasonable as our sync precision is limited by approximately half the granularity of the master AWGs at each node. We take this mismatch into account in the overall time budget of the experiment by conservatively adding it to both $A \to B$ and $B \to A$ protocol durations. Hence from the steadiness of the timing and clock matching precision we can conclude that the locality loophole was closed during the whole data acquisition run.

IV. TIMING VERIFICATION

In this section, we present our method of characterising the space-time configuration of the experiment, in order to ensure that the locality loophole is closed. As mentioned in the main text, we define the start time $t_\star$ of each trial of the randomness amplification protocol as the time at which the local random number generators start generating a random input bit (see Fig. 4 in the main text). The stop time $t_\times$ marks the time when the untrusted qubit readout signal arrives at the input

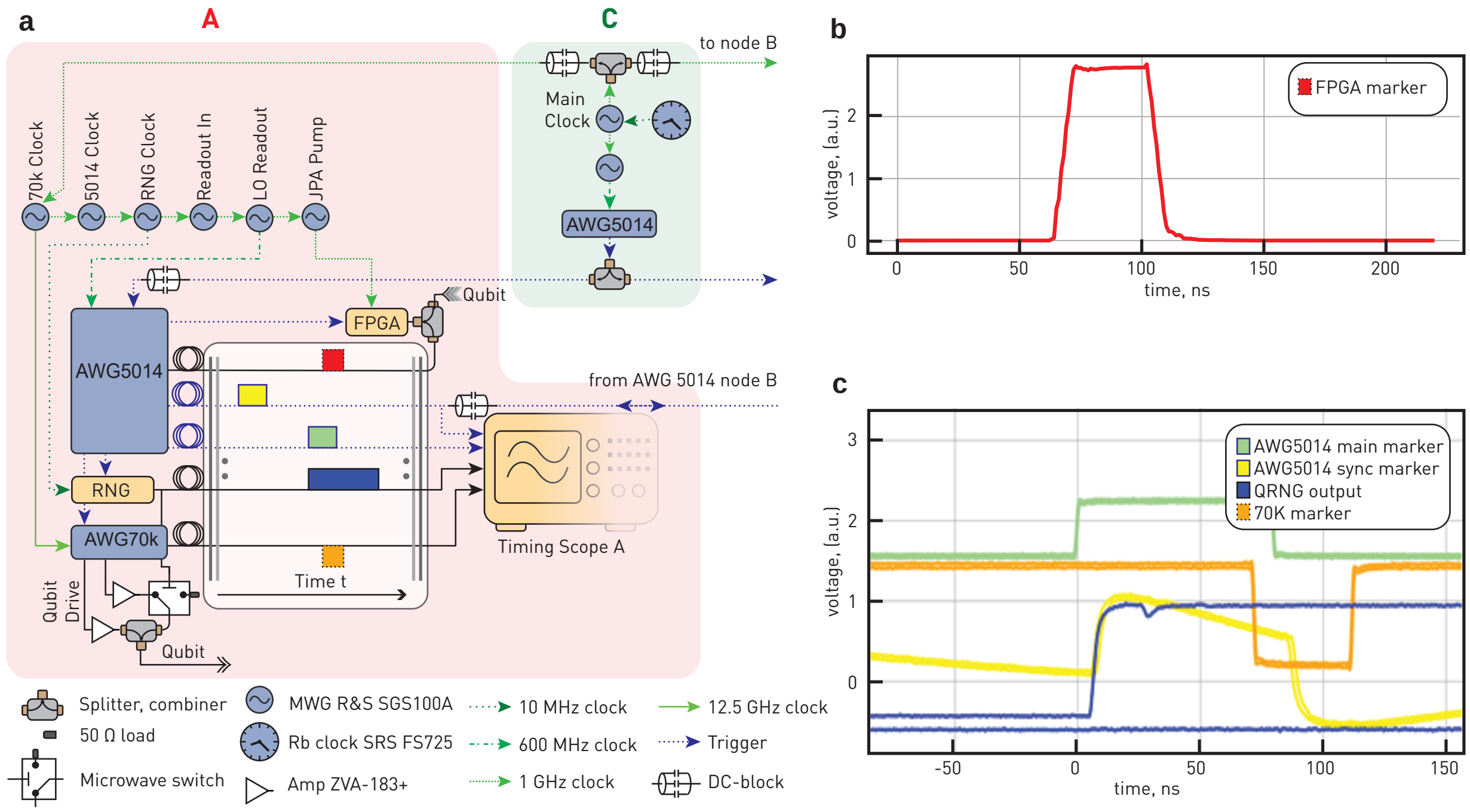

FIG. S2. **a**, Clock and trigger distribution scheme for the synchronization of the room-temperature electronics at node A and center C, the scheme is identical at node B. Inset: pulses and connections to oscilloscope and FPGA to monitor and measure the output delays of various devices. Yellow: auxiliary pulse from the master device (AWG 5014) at another node; Green: timing reference pulse. Blue: QRNG output signal; Orange: a negative image of a fixed-time synchronization pulse from the AWG 70k. **b,c**, An example of the measured waveforms on an FPGA and an oscilloscope. The signal on the FPGA corresponds to the pulse output from AWG5014 at a fixed time and is used to synchronize the FPGA to the global lab timeframe. The oscilloscope pulses correspond to the ones shown in the inset **a** with same color code. The green reference pulse defines the zero time; the yellow pulse travelling from another node is used to ascerain the nodes' synchronization. The blue and orange pulses provide the information about the RNG and AWG70k reference frame synchronization. Waveforms with dotted lines in **a** are only generated and measured between the acquisition blocks, while the others are always available

of the analog-to-digital converter (ADC), part of the trusted laboratory.

The locality loophole is closed when both of the following conditions hold: the basis choice at node A must not be able to influence the measurement at node B using influences propagating at most at the speed of light c, and vice versa. In order to ensure that, we must precisely know where and when the start and stop events happen.

In a first step, we measure the spatial distance between the locations corresponding to the start event at one node and the stop event at the other node, in a scheme discussed in Ref. [4]. We find the shorter of these two distances to be the one from the start event at node B to the stop event at node A, measuring $d = 32.928\,\text{m}$. This defines the Bell distance, and thereby the available duration during which the exchange of classical information exchange between the two nodes is forbidden, $t_d = d/c = 109.83$ ns.

In a second step, we verify that the actual duration of the protocol $t_{\text{protocol}} = t_\times - t_\star$ is shorter than the budget t_d imposed by the Bell distance. We perform the analysis for both critical durations, i.e. for $t_{\text{protocol},A\to B}$ referring to the duration between the start event at A and the stop event at B, and vice versa. In the following, for simplicity, we focus on $t_{\text{protocol},A\to B}$, and the calculation of the $t_{\text{protocol},B\to A}$ follows accordingly.

At the beginning of the experiment, it is critical to establish a common reference frame for the devices involved in the experiment. For this purpose, we install an oscilloscope at each node (part of the trusted setup), to detect the arrival time of specific reference pulses output by electronic devices in the setup (part of λ). We define the reference time $t_{0,\text{OsciA}}$ as the moment when oscilloscope A detects a reference pulse from the local master arbitrary waveform generator (AWG), sent through a cable with a well-characterized electrical length. We refer the interested reader to Ref. [4] for more details.

In a next step, we determine the protocol duration $t_{\text{protocol},A\to B}$. As we do not have direct experimental access to the start and stop events defined above, we extract $t_{\text{protocol},A\to B}$ by performing a set of measurements of the duration of the individual segments of the protocol. For this purpose, it is instructive to express the protocol duration in terms of its individual constituents,

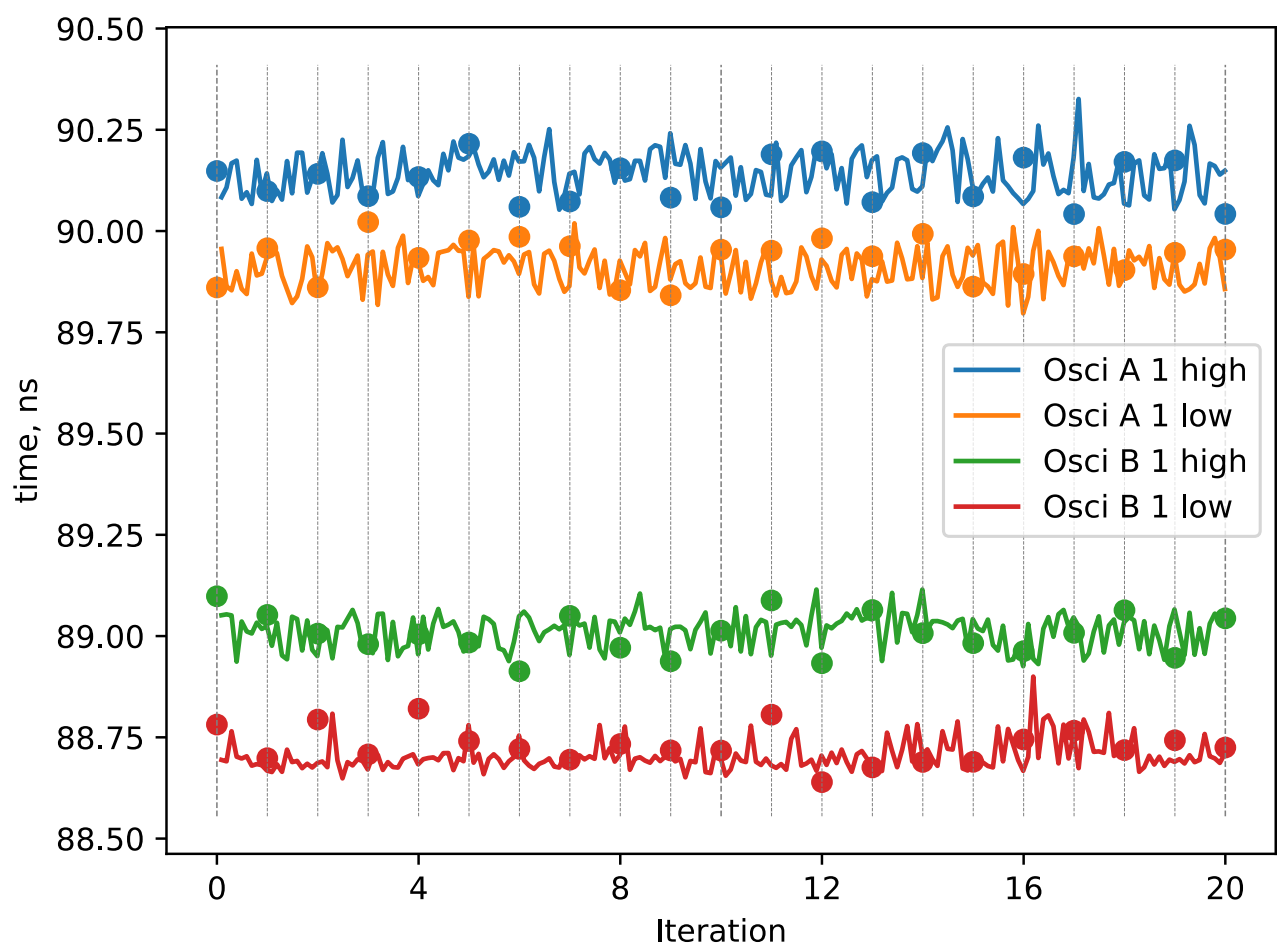

FIG. S3. **Inter-node timing stability and synchronisation.** Data for measurements of an auxiliary pulse arriving from another node to an oscilloscope installed at node A (blue and orange) and B (green and red). Each datapoint corresponds to several thousand triggers recorded in the envelope mode on an oscilloscope; Plotted are both high (late) and low (early) points of the envelope trace crossing the pre-defined threshold. Presence of jitter would manifest as the high and low points corresponding to the same device being different by the device granularity, 1.66 ns. The existing difference between the high and low envelope traces reflects the limited sampling rate of the oscilloscope (2.5 GS/s) and possible jitter on the order of 100 ps, which is within our uncertainly calculations. Full circles correspond to datapoints taken between the data blocks, and continuous lines correspond to the on-the-fly timing verification measurements.

$$t_{\text{protocol},A\to B} = t_{\times B} - t_{\star,A} \tag{1}$$
$$= t_{\text{detection,B}} + t_{\Delta B} \tag{2}$$
$$+ t_{\Delta AB} + t_{\text{latch,A}} + t_{\text{RNG,A}}. \tag{3}$$

Fig. S5 provides a graphical illustration of the individual segments of the protocol. Here $t_{\text{detection,B}}$ marks the length of the data acquisition, or the difference of the time at which the ADC at node B starts acquiring data and the time when it stops ($t_{\times B}$). We can experimentally control this duration by programming the field-programmable gate array (FPGA), which is connected to the ADC, to have zero acquisition weights after the specified time. Typically, however, the FPGA is not perfectly synchronized to the node reference frame $t_{0,\text{OsciB}}$. To account for differences between the internal reference frame of the local FPGA, $t_{0,\text{FPGAB}}$, and the node reference frame $t_{0,\text{OsciB}}$, we include their offset $t_{\Delta B} = t_{0,\text{FPGAB}} - t_{0,\text{OsciB}}$ in the expression above. This offset is measured in a separate calibration experiment, where we compare the arrival time of a microwave square pulse recorded by the local oscilloscope and by the local FPGA.

Next, $t_{\text{RNG,A}}$ denotes the time it takes for the local RNG to generate a random bit. The random number generators used in this experiment [6] produce random bits based on the principle of phase diffusion of a laser, founded on the underlying quantum mechanical process of spontaneous emission. More specifically, $t_{\text{RNG,A}}$ is the duration between the laser injection current crossing the lasing threshold and the moment the RNG outputs the random bit in the form of a differential voltage, as described in Ref. [6]. We have more direct experimental access to the latching event, and define the time between the RNG latching a random bit and the node reference time $t_{0,\text{OsciA}}$ as $t_{\text{latch,A}}$ (Fig. S5).

Quantity at A	Value (ns)	Quantity at B	Value (ns)
$t_{\text{RNG,A}}$	17.1	$t_{\text{RNG,B}}$	17.1
$t_{\text{latch,A}}$	2.0	$t_{\text{latch,B}}$	7.2
$t_{\Delta A}$	5.0	$t_{\Delta B}$	2.0
$t_{\Delta AB}$	0.55		
$t_{\text{detection,A}}$	86.0	$t_{\text{detection,B}}$	94.0
$t_{\text{protocol},A\to B}$	106.65	$t_{\text{protocol},B\to A}$	106.45

TABLE S2. Measured durations of individual segments of the protocol, as described in the text.

Finally, we take into account the imperfect synchronization of the two node reference frames, $t_{\Delta AB}$. We determine this synchronisation offset by sending a reference pulse from an AWG at node A to the oscilloscope at node B, and vice versa, through the same cables. This allows us to compare the respective arrival times of the pulses, and thereby determine $t_{\Delta AB}$.

We measure all quantities during the randomness amplification experiment via a dedicated timing test sequence. Furthermore, we verify the absence of jitter of electronic devices involved in the setup continuously during the experiment, by monitoring the arrival time of reference pulses output by the relevant devices on the local oscilloscopes.

As no jitter was detected during the main experiment, and as the corresponding timing measurements (section III) yielded consistent results (up to the measurement precision), we ascertain the locality loophole to be closed. From the measured individual protocol segment durations introduced above, see Table S2, we conclude that the total duration of the protocol was no more than $t_{\text{protocol},A\to B} = 106.7$ ns and therefore $t_d - t_{\text{protocol},A\to B} = 3.1$ ns shorter than the budget imposed by the Bell distance. The standard deviation on the budget is on the order of a few tens of picoseconds, the one on the protocol duration on the order of a few hundred picoseconds. The achieved margin between protocol duration and budget is about 50% higher than for the loophole-free Bell test presented on the same setup [4], and it is, constituting about 2.8% of the available time budget, in a similar regime as other loophole-free Bell tests [7–10]. For more detailed information on the timing analysis, we refer to the Supplementary Information of Ref. [4].

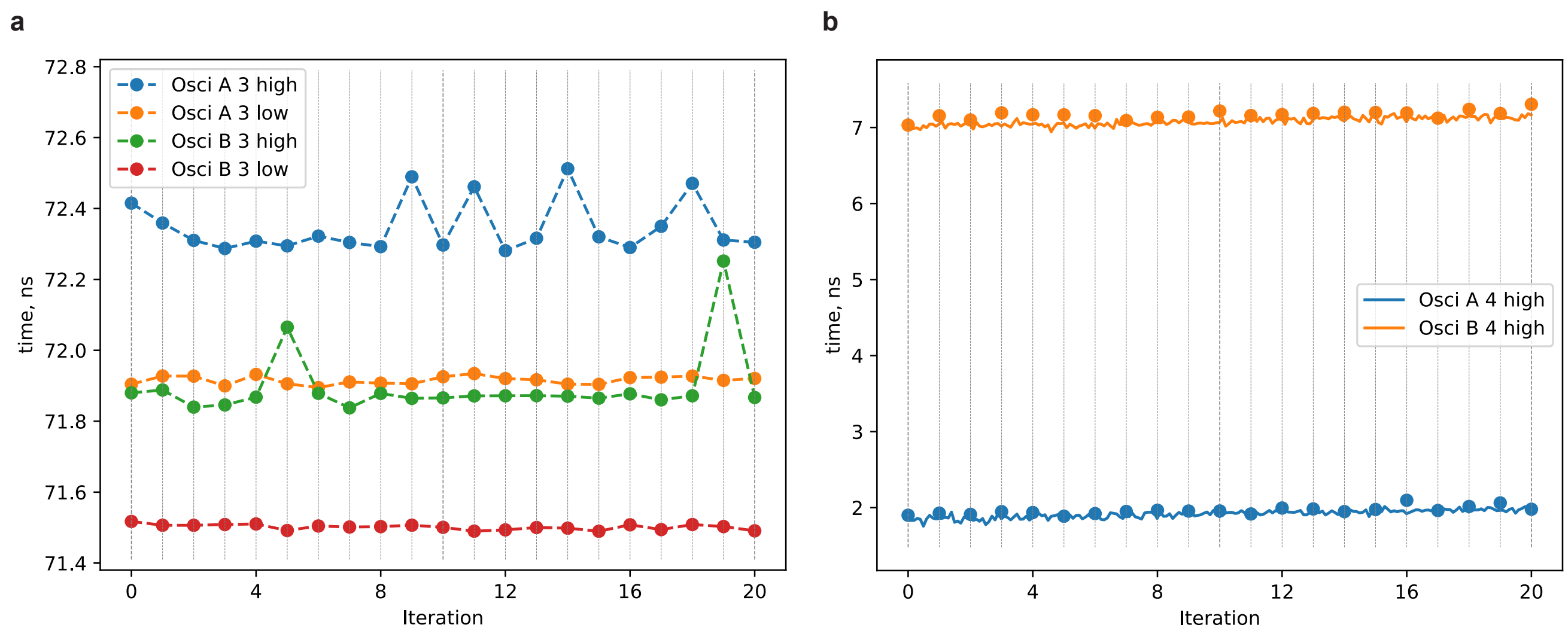

FIG. S4. **Timing at a single node a**, Data showing the arrival time of a timing calibration pulse from the AWG70k used to control qubit rotations at node A (blue and orange) and B (green and red). As in Fig. S3, each datapoint corresponds to several thousand triggers recorded in the envelope mode on an oscilloscope; Plotted are both high (late) and low (early) points of the envelope trace crossing the pre-defined threshold. These timings are only measured between the data acquisition blocks. **b**, Data showing the arrival time of a QRNG switching pulse and hence defining the starting time of each trial. Compare to Table S2. Full circles correspond to datapoints taken between the data blocks, and continuous lines correspond to the on-the-fly timing verification measurements.

V. ASSUMPTIONS

For randomness amplification to be feasible, a number of assumptions need to be satisfied. In this section, we explicitly list the assumptions, discuss their justification and conclude with a discussion on which assumption could, in principle, be weakened.

We make the following assumptions:

1. Quantum mechanics is correct and complete. In particular, the adversary can be described using quantum mechanics.
2. The classical information processing devices are trusted. In particular, this includes the ADC, the timing verification and the computer used for classical post-processing.
3. The two sources of randomness are, possibly correlated, μ-SV sources with quantum side information. For a formal definition of μ-SV sources, see Section VI.
4. The device λ does not signal to the two sources of randomness. For a formal definition of non-signaling, see Definition 5 in Section VI.

Assumption 4 is justified by separating the QRNGs from the device λ [11]. In principle, the assumption follows from relativity and could therefore be avoided if the QRNGs were placed sufficiently far from the device λ to ensure spacelike separation between the protocol rounds. However, as such a placement of the QRNGs is infeasible in our current experimental setup, we explicitly include Assumption 4. Finally, we remark that one also sometimes assumes that, in addition to the previous assumptions, the device λ is shielded from the adversary. This is required if one wants the random bitstring $\mathbf{K}$ to remain private after it was produced. However, there are numerous applications where a source of public randomness is sufficient (for instance, lotteries or randomness beacons [12]) and therefore we do not impose this assumption.

There are several directions in which the above assumptions may be weakened. For instance, some prior works require the two sources to only produce outputs with a sufficient amount of min-entropy, instead of requiring them to be μ-SV sources [13–15]. However, these protocols either require Bell tests with many parties [13, 14] or only allow for classical side-information about the source [15]. Similarly, some protocols have better noise tolerance but require a tripartite Bell test which is difficult to implement in a loophole free manner [16]. Another direction is to relax the assumption that the adversary is constrained by quantum mechanics and instead consider adversaries who are only required to follow the non-signaling principle [17–19]. In our opinion, these protocols are impractical as they either require Bell tests with many parties [18], are non-constructive [19], or have vanishing extraction rate [17, 19]. Hence, the above assumptions are, to the best of our knowledge, the minimal set of assumptions under which randomness amplification is possible using current technology.

Nevertheless, there are approaches to randomness generation based on a different set of assumptions which are neither stronger nor weaker than the ones listed at the beginning of this section. One example here are protocols for *device-*

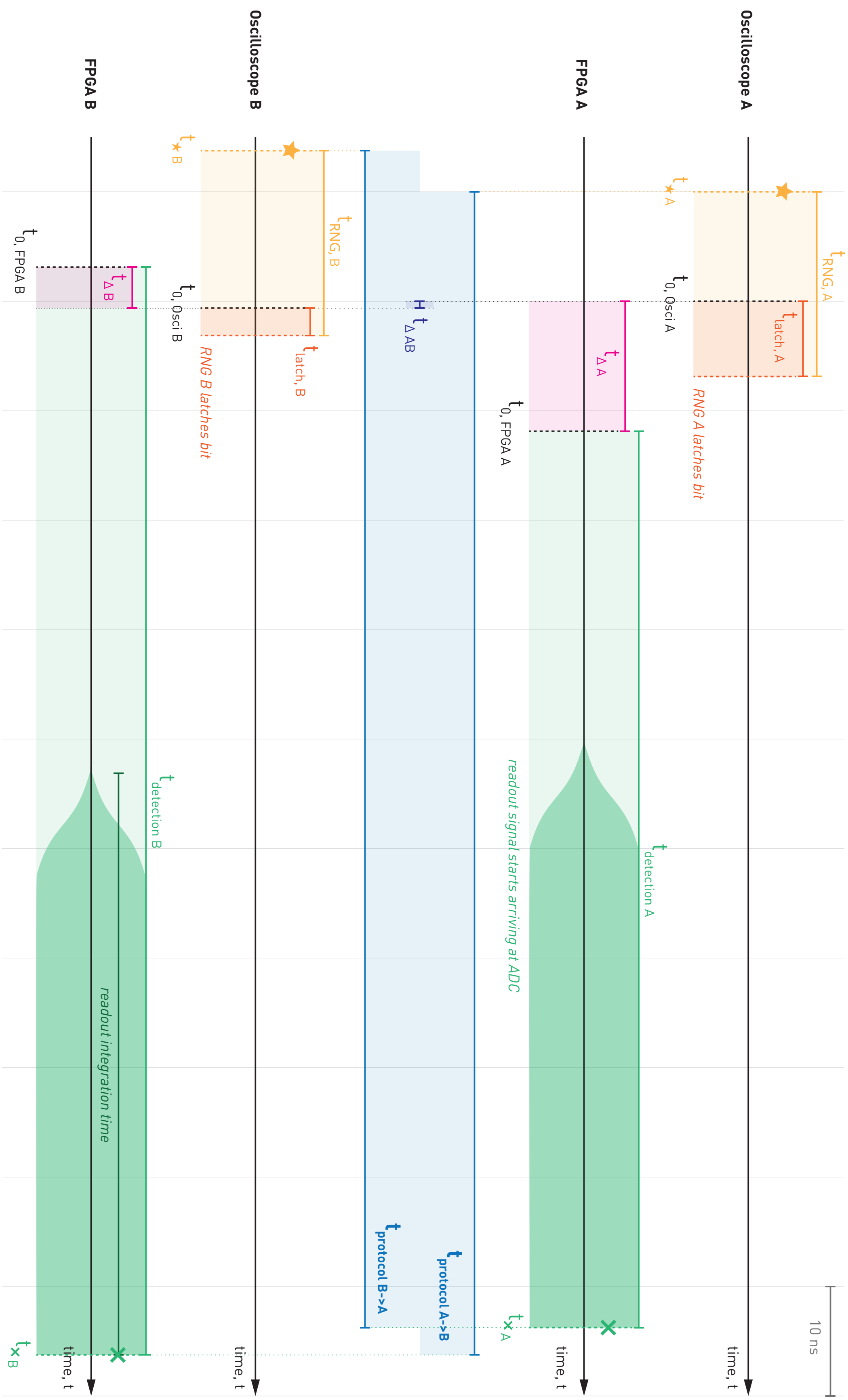

FIG. S5. Timing overview of the randomness amplification experiment. See section IV for details.

independent randomness expansion [20–23], which require a short but perfect initial randomness, sometimes called *seed randomness*. In contrast, we assume to have access to two μ-SV sources that provide a large number of imperfect random bits. Furthermore, there are protocols which can produce certified randomness using a single device [24–26]. This allows them to generate randomness from an untrusted server, whereas our protocol requires trusting the party implementing the Bell test (see Assumption 2). However, achieving this task requires assuming bounds on the adversary's computational power, which are unnecessary for our protocol.

More generally, assumptions about an adversary's capabilities are difficult to justify, as they require forecasting technological progress over the decades-long lifespan typical of cryptographic protocols. For example, one cannot rule out a future in which adversaries possess hardware capable of entangling systems with other parties' devices. This also motivates our use of a definition of SV sources that allows for quantum side information, placing the analysis on the safe side.

VI. SECURITY PROOF

In this section we present the formal security proof for the protocol in Box I. Table S3 below summarizes the notation used throughout this section.

We begin by formally introducing the notion of a μ-SV source with quantum side information, which provides the weakly random inputs to the Bell test.

SV sources with quantum side information

Definition 1 (SV source with quantum side information)**.** *An SV source with quantum side information and bias μ is a sequence of instruments $\{\mathcal{S}^i_{B_iQ_i|Q_{i-1}}\}_i$, where B_i is a single bit, such that*

$$H_{\min}(B_i|\tilde{Q}_{i-1})_{\mathcal{S}^i[\rho]} \geq -\log_2\left(\frac{1}{2}+\mu\right) \quad \forall i, \rho_{Q_{i-1}\tilde{Q}_{i-1}}. \quad (4)$$

In the following, we will also use the term "quantum SV source" for brevity.

Note that the above definition can be understood as requiring that the adversary (who has access to $\tilde{Q}_{i-1}$) cannot guess the bit B_i with probability more than $\frac{1}{2}+\mu$ [28].

Remark 1 (Characterization using non-optimized min-entropy)**.** *By [29, Proposition 19], Definition 1 is equivalent to*

$$H^{\downarrow}_{\infty}(B_i|\tilde{Q}_{i-1})_{\mathcal{S}^i[\rho]} \geq -\log_2\left(\frac{1}{2}+\mu\right) \quad \forall i, \rho_{Q_{i-1}\tilde{Q}_{i-1}}. \quad (5)$$

The following lemma shows that the entropy of a μ-SV source accumulates.

Lemma 1 (Chaining of entropy)**.** *Let $\{\mathcal{S}^i_{B_iQ_i|Q_{i-1}}\}_{i=1}^n$ be a quantum SV source with bias μ. Then, for any state $\rho_{Q_0\tilde{Q}_0}$, the state $\rho^{\text{out}}_{B^nQ_n\tilde{Q}_0} := (\mathcal{S}^n\circ\ldots\circ\mathcal{S}^1)[\rho_{Q_0\tilde{Q}_0}]$ satisfies*

$$H_{\min}(B^n|\tilde{Q}_0)_{\rho^{\text{out}}} \geq -n\log_2\left(\frac{1}{2}+\mu\right). \quad (6)$$

Proof. One can directly bound

$$H_{\min}(B^n|\tilde{Q}_0)_{\rho^{\text{out}}} \geq \sum_{i=1}^n H^{\downarrow}_{\infty}(B_i|B^{i-1}\tilde{Q}_0)_{\rho^{\text{out}}}, \quad (7)$$

where we used the chain rule from [27, Proposition 5.12] n times. Next, using Remark 1, we know that

$$H^{\downarrow}_{\infty}(B_i|B^{i-1}\tilde{Q}_0)_{\rho^{\text{out}}} \geq -\log_2\left(\frac{1}{2}+\mu\right) \quad (8)$$

holds for all i. This concludes the proof. □

For Bell tests with imperfect inputs, we require the following condition for the input randomness (a classical version of which was introduced in [30]).

Definition 2 (MDL source with quantum side information)**.** *Let $\mathcal{S}_{XYQ'|Q}$ be a channel where X and Y each are a single bit. We call $\mathcal{S}_{XYQ'|Q}$ a $(\mu_{\min},\mu_{\max})$*-MDL source with quantum side information *if for every state $\rho_{Q\tilde{Q}}$, the state $\rho^{\text{out}}_{XYQ'\tilde{Q}} := \mathcal{S}_{XYQ'|Q}[\rho_{Q\tilde{Q}}]$ satisfies*

$$\rho^{\text{out}}_{\tilde{Q}\wedge xy} \leq \mu_{\max}\rho_{\tilde{Q}} \quad \text{and} \quad \rho^{\text{out}}_{\tilde{Q}\wedge xy} \geq \mu_{\min}\rho_{\tilde{Q}} \quad \forall x,y. \quad (9)$$

In the following, we will also use the term "quantum MDL source" for brevity. The states appearing on the left hand side of (9) are the sub-normalized states conditioned on x and y, see Table S3.

In our analysis, we treat the two sources of imperfect randomness as a single μ-SV source. The following lemma shows that this can be seen as a single MDL source.

Lemma 2. *Let $\{\mathcal{S}^1_{XQ_1|Q_0},\mathcal{S}^2_{YQ_2|Q_1}\}$ be a quantum SV source with bias μ. Then $\mathcal{S}_{XYQ_2|Q_0} = \mathcal{S}^2_{YQ_2|Q_1}\circ\mathcal{S}^1_{XQ_1|Q_0}$ is a quantum $((\frac{1}{2}-\mu)^2,(\frac{1}{2}+\mu)^2)$-MDL source.*

Proof. Let $\rho_{Q_0\tilde{Q}_0}$ be an arbitrary quantum state and denote $\rho^1_{XQ_1\tilde{Q}_0} := \mathcal{S}^1_{XQ_1|Q_0}[\rho_{Q_0\tilde{Q}_0}]$. By the SV property (see Remark 1), we have that

$$\rho^1_{\tilde{Q}_0\wedge x} \leq \left(\frac{1}{2}+\mu\right)\rho_{\tilde{Q}_0} \quad \forall x\in\{0,1\}, \quad (10)$$

and by $\rho^1_{\tilde{Q}_0\wedge 0}+\rho^1_{\tilde{Q}_0\wedge 1} = \rho^1_{\tilde{Q}_0} = \rho_{\tilde{Q}_0}$ it also holds for all $x\in\{0,1\}$ that

$$\rho^1_{\tilde{Q}_0\wedge x} = \rho_{\tilde{Q}_0} - \rho^1_{\tilde{Q}\wedge(1-x)} \geq \left(\frac{1}{2}-\mu\right)\rho_{\tilde{Q}_0}. \quad (11)$$

Applying the same argument again to $\mathcal{S}^2_{YQ_2|Q_1}$ and $\rho^1_{XQ_1\tilde{Q}_0}$ gives the desired statement. □

Symbol	Definition
$\mathbb{P}_{\mathcal{C}}$	Set of probability distributions over the alphabet $\mathcal{C}$
ρ_{XA}	Classical-quantum (cq) state. That is, a state of the form $\rho_{XA} = \sum_x p(x)\,\|x\rangle\langle x\|_X \otimes \rho_{A\|x}$
$\rho_{A\wedge x}$	Sub-normalized conditional state, i.e, $\rho_{A\wedge x} = \langle x\|\rho_{XA}\|x\rangle_X = p(x)\rho_{A\|x}$
$\rho_{XA\|\Omega}$	State conditioned on $\Omega \subseteq \mathcal{X}$. Explicitly $\rho_{XA\|\Omega} = \frac{1}{\Pr[\Omega]}\sum_{x\in\Omega} p(x)\,\|x\rangle\langle x\|_X \otimes \rho_{A\|x}$, with $\Pr[\Omega] = \sum_{x\in\Omega} p(x)$
A^n	Shorthand for $A_1 \dots A_n$
$\mathcal{E}_{B\|A}$	Completely positive and trace-preserving (CPTP) map or channel $\mathcal{E}_{B\|A} \in \mathrm{Hom}\left(\mathrm{End}(\mathcal{H}_A), \mathrm{End}(\mathcal{H}_B)\right)$
$\mathcal{M}_{XS\|A}$	A quantum instrument, i.e., a quantum channel where X is a classical register
$\mathcal{I}_R$	Identity channel on the system R
POVM	A *positive operator valued measure*. That is, a set of positive operators $\{M_A(a)\}_a$ such that $\sum_a M_A(a) = \mathbb{1}_A$
$\mathcal{E}_{B\|A}[\rho_{AR}]$	Application of a channel to a state of a larger system: $(\mathcal{E}_{B\|A} \otimes \mathcal{I}_R)[\rho_{AR}]$
$f_{Y\|X}[\rho_{XR}]$	Notation for $f_{Y\|X}[\rho_{XR}] = \sum_x \|f(x)\rangle\langle f(x)\|_Y\ \langle x\|\rho_{XR}\|x\rangle_X$, i.e., the function f applied as a channel
$f_{YX\|X}[\rho_{XR}]$	The same as $f_{Y\|X}[\rho_{XR}]$ but with a copy of X appended to the output. Explicitly, $f_{YX\|X}[\rho_{XR}] = \sum_x \|f(x)\rangle\langle f(x)\|_Y \otimes \|x\rangle\langle x\|_X \otimes \langle x\|\rho_{XR}\|x\rangle_X$
$\\|M\\|_1$	The Schatten 1-norm of M given by $\\|M\\|_1 = \mathrm{tr}\left[\sqrt{MM^*}\right]$
h_2	The binary entropy. Explicitly, $h_2(p) = -p\log_2 p - (1-p)\log_2(1-p)$
$H_{\min}(A\|B)_\rho$	Conditional min-entropy. See, for instance, [27]
$H_\infty^{\downarrow}(A\|B)_\rho$	The unoptimized conditional min-entropy. Explicitly $H_\infty^{\downarrow}(A\|B)_\rho = \max\{\lambda : \rho_{AB} \le 2^{-\lambda}\mathbb{1}_A \otimes \rho_B\}$
$H_{\min}^{\varepsilon}(A\|B)_\rho$	The smooth min-entropy. Explicitly, $H_{\min}^{\varepsilon}(A\|B) := \max_{\tilde\rho \in \mathcal{B}_\rho^\varepsilon} H_{\min}(A\|B)_{\tilde\rho}$ [27]. Here $\mathcal{B}_\rho^\varepsilon$ denotes the ε-Ball around ρ in terms of purified distance.

TABLE S3. Summary of notations. We use letters E,Q,R,S,T to denote quantum systems and labels X, Y, Z, C to denote classical systems.

Two-process extractors

The goal of a device-independent randomness amplification protocol is to produce a string of uniformly random bits. To achieve this, one needs to process the imperfect random outputs **AB** of a Bell-test, together with some randomness **Z** from the sources, into a string of (almost) perfectly random bits (see also Fig. 1 in the main text). Our main technical tool for this task are the two-process extractors introduced in [31]. Here, we reproduce the main notions and results of this work. We begin by introducing the notion of two-process extractors which allow for the extraction of uniform randomness from independent and sufficiently random processes.

Definition 3 (Two-process extractor)**.** *Let $k_1, k_2, \varepsilon \ge 0$. We call a function* $\mathrm{Ext}: \{0,1\}^{n_1} \times \{0,1\}^{n_2} \to \{0,1\}^m$ *a* (k_1,k_2,ε)-weak two-process extractor *if for all pure states ρ_{AB} and all instruments $\mathcal{M}_{XS|A}$ and $\mathcal{N}_{YT|B}$ with*

$$H_{\min}(X|SB)_{\mathcal{M}[\rho]} \ge k_1 \quad \text{and} \quad H_{\min}(Y|TA)_{\mathcal{N}[\rho]} \ge k_2, \tag{12}$$

the state $\rho^{\mathrm{out}}_{XYST} := \left(\mathcal{M}_{XS|A} \otimes \mathcal{N}_{YT|B}\right)[\rho_{AB}]$ is such that

$$\frac{1}{2}\left\|\mathrm{Ext}_{Z|XY}[\rho^{\mathrm{out}}_{XYST}] - \omega_Z \otimes \rho^{\mathrm{out}}_{ST}\right\|_1 \le \varepsilon. \tag{13}$$

Similarly, we call Ext *a* (k_1,k_2,ε) two-process extractor strong in Y, *if for all instruments and states as above with*

$$H_{\min}(X|SB)_{\mathcal{M}[\rho]} \ge k_1 \quad \text{and} \quad H_{\min}(Y|A)_{\mathcal{N}[\rho]} \ge k_2, \tag{14}$$

the state $\rho^{\mathrm{out}}_{XYST} := \left(\mathcal{M}_{XS|A} \otimes \mathcal{N}_{YT|B}\right)[\rho_{AB}]$ is such that

$$\frac{1}{2}\left\|\mathrm{Ext}_{ZY|XY}[\rho^{\mathrm{out}}_{XYST}] - \omega_Z \otimes \rho^{\mathrm{out}}_{YST}\right\|_1 \le \varepsilon. \tag{15}$$

Definition 3 is stated assuming the initial state ρ_{AB} is pure. This is to ensure that the conditions in (12) and (14) are sufficient to guarantee that the processes $\mathcal{M}$ and $\mathcal{N}$ produce new entropy. However, note that Definition 3 can be readily applied to non-pure inputs ρ_{AB} by considering a purification ρ_{ABE} and then applying Definition 3 to ρ_{ABE}, $\mathcal{M}_{XS|A}$, and $\mathcal{N}_{YT|B} \otimes \mathrm{tr}_E$. The conditions in (12) then read

$$H_{\min}(X|SBE)_{\mathcal{M}[\rho]} \ge k_1 \quad \text{and} \quad H_{\min}(Y|TA)_{\mathcal{N}\otimes\mathrm{tr}_E[\rho]} \ge k_2. \tag{16}$$

The model described in Definition 3 allows for the extraction of randomness from two independent processes. For the purposes of device-independent randomness amplification, it will be more convenient to consider the scenario where only a single channel is applied to some initial state. The following lemma shows that randomness extraction in this scenario can be achieved using two-process extractors.

Lemma 3 ([31, Lemma B.1])**.** *Let ρ_{XB} be a cq state and $\mathcal{N}_{YT|B}$ be an instrument. Assume that $H_{\min}(X|B)_\rho \ge k_1$ and $H_{\min}(Y|R)_{\mathcal{N}[\sigma]} \ge k_2$ hold where σ_{BR} is a purification of ρ_B. Let* Ext *be a (k_1,k_2,ε) two-process extractor strong in Y. Then*

$\rho^{\text{out}}_{XYT} := \mathcal{N}_{YT|B}[\rho_{XB}]$ *is such that*

$$\frac{1}{2}\left\|\text{Ext}_{ZY|XY}[\rho^{\text{out}}_{XYT}] - \omega_Z \otimes \rho^{\text{out}}_{YT}\right\|_1 \le \varepsilon. \tag{17}$$

To extract our final bitstring $\mathbf{K}$, we will use the following construction.

Definition 4 (Dodis et al.'s construction [32])**.** *Let $\mathcal{K} = \{K_i\}_{i=1}^m$ be a set of $n \times n$ matrices with entries in $\{0,1\}$ such that for any $0 \ne s \in \{0,1\}^m$ it holds that*

$$\text{rank}\left(\sum_{i=1}^m s_i K_i\right) \ge n - r \tag{18}$$

for some $r \in \mathbb{N}$. The function $DEOR^{\mathcal{K}} : \{0,1\}^n \times \{0,1\}^n \to \{0,1\}^m$ is defined as

$$\text{DEOR}^{\mathcal{K}}(x,y) := (x^T K_1 y, \ldots, x^T K_m y). \tag{19}$$

In (18) and (19), addition is taken modulo 2.

Of particular interest to us will be the case when K_i is the non-cyclic right shift matrix by $i-1$ positions. It can be shown that for this construction one has $r = m$ [32]. Furthermore, there are efficient implementations of this extractor [33].

The following theorem shows that $\text{DEOR}^{\mathcal{K}}$ is a two-process extractor.

Theorem 1 ([31, Theorem 5.5])**.** $\text{DEOR}^{\mathcal{K}}$ *is a (k_1,k_2,ε) two-process extractor, strong in Y, with*

$$\varepsilon = \frac{1}{2}\sqrt{2^{2m+n+r-k_1-k_2}}. \tag{20}$$

Entropy accumulation

As stated in Definition 3 (see also Lemma 3), a two-process extractor requires a guarantee on the (smooth) min-entropy of its input. In our protocol, the inputs are $\mathbf{AB}$ and $\mathbf{Z}$ (compare Fig. **??**). By Lemma 3, the randomness of $\mathbf{Z}$ is guaranteed since it was produced by a quantum SV-source. Bounding the entropy of $\mathbf{AB}$ on the other hand is a lot more challenging. Several methods have been developed to derive these kinds of bounds. Of particular interest are the entropy accumulation theorem (EAT) [34] and the framework of quantum probability estimation (QPE) [35] (see [36] for an application of QPE to device-independent randomness expansion). In [11], the EAT was used to prove the security of DIRA against adversaries holding only classical side information about the SV sources. However, since we consider the more general quantum side information, the Markov chain condition of the EAT and the QPE frameworks are no longer satisfied. To overcome this limitation, we utilize the *generalized entropy accumulation theorem (GEAT)* [37] which requires a weaker non-signaling condition. We will first introduce the GEAT and then demonstrate how our setup can be formulated in the language of the GEAT.

We begin by formally introducing the notion of non-signaling.

Definition 5. *Let $\mathcal{M}_{CD|AB}$ be a quantum channel. We say that $\mathcal{M}$ is* non-signaling from A to C *if there exists a channel $\mathcal{R}_{C|B}$ such that*

$$\text{tr}_D \circ \mathcal{M}_{CD|AB} = \mathcal{R}_{C|B} \circ \text{tr}_A\,. \tag{21}$$

Intuitively, Definition 5 can be seen as requiring that no information flows from system A to system C. In the GEAT, such a non-signaling condition is required to impose that the channels do not leak any information from the memory of the devices to the adversary. Additionally, the GEAT includes registers C_i which can be used to collect statistics about the behavior of the channels (see Fig. S6). This is captured by the following definition.

Definition 6 (GEAT channel)**.** *We call a channel $\mathcal{M}_{C_iA_iR_iE_i|R_{i-1}E_{i-1}}$ a GEAT channel if $\mathcal{M}$ does not signal from R_{i-1} to E_i and there exists a channel $\mathcal{T}_{C_iA_iE_i|A_iE_i}$ such that*

$$\mathcal{M}_{C_iA_iR_iE_i|R_{i-1}E_{i-1}} = \mathcal{T}_{C_iA_iE_i|A_iE_i} \circ \text{tr}_{C_i} \circ \mathcal{M}_{C_iA_iR_iE_i|R_{i-1}E_{i-1}} \tag{22}$$

and $\mathcal{T}$ has the form

$$\begin{aligned}\mathcal{T}_{C_iA_iE_i|A_iE_i}[\omega_{A_iE_i}] = \sum_{a,e}(\Pi^{(a)}_{A_i} \otimes \Pi^{(e)}_{E_i})\,\omega_{A_iE_i}(\Pi^{(a)}_{A_i} \otimes \Pi^{(e)}_{E_i}) \\ \otimes |r(a,e)\rangle\langle r(a,e)|_{C_i}\,,\end{aligned} \tag{23}$$

where $r(a,e)$ is a deterministic function and $\Pi^{(a)}_{A_i}, \Pi^{(e)}_{E_i}$ are projectors.

The condition in (22) says that the testing register C_i can be reconstructed from A_i and E_i in projective way. Note that this condition is always satisfied when C_i is computed by a deterministic function from classical information contained in A_i and E_i. This will be the case for our protocol.

The GEAT bounds the smooth min-entropy $H^{\varepsilon_s}_{\min}(\mathbf{A}|E_n)$ in terms of the single-round von Neumann entropy. In addition, the GEAT allows to constrain the channels $\mathcal{M}_i$ using the observed frequency of the registers C_i. This is formalized using the notion of a min-tradeoff function.

Definition 7 (Min-tradeoff function)**.** *Let $\mathcal{C}$ be a finite set, $\mathbb{P}_{\mathcal{C}}$ be the set of probability distributions over $\mathcal{C}$, and $\{\mathcal{M}_i\}_i$ be a a set of GEAT channels. For $i \in \{1,\ldots,n\}$ and $q \in \mathbb{P}_{\mathcal{C}}$, define*

$$\begin{aligned}\Sigma_i(q) := \{\nu_{A_iC_iE_i\tilde{E}_{i-1}} = \mathcal{M}_i[\omega_{R_{i-1}E_{i-1}\tilde{E}_{i-1}}] : \\ \omega_{R_{i-1}E_{i-1}\tilde{E}_{i-1}} \in \mathcal{S}(R_{i-1}E_{i-1}\tilde{E}_{i-1}) \wedge \nu_{C_i} = q\},\end{aligned} \tag{24}$$

where we interpret ν_{C_i} as the probability distribution over $\mathcal{C}$ with probabilities $\Pr[c] = \langle c|\nu_{C_i}|c\rangle$. An affine function $f_{\min} : \mathbb{P}_{\mathcal{C}} \to \mathbb{R}$ is called a min-tradeoff function *if*

$$f(q) \le \inf_{\nu \in \Sigma_i(q)} H(A_i|E_i\tilde{E}_{i-1})_\nu \quad \forall i \in \{1,\ldots,n\}. \tag{25}$$

Definition 8 (Frequency distribution)**.** *Let $\mathbf{c} \in \mathcal{C}^n$. Define the frequency distribution $\text{freq}_{\mathbf{c}} \in \mathbb{P}_{\mathcal{C}}$ by*

$$\text{freq}_{\mathbf{c}}(c') := \frac{|\{i : c_i = c'\}|}{n} \quad \forall c' \in \mathcal{C}. \tag{26}$$

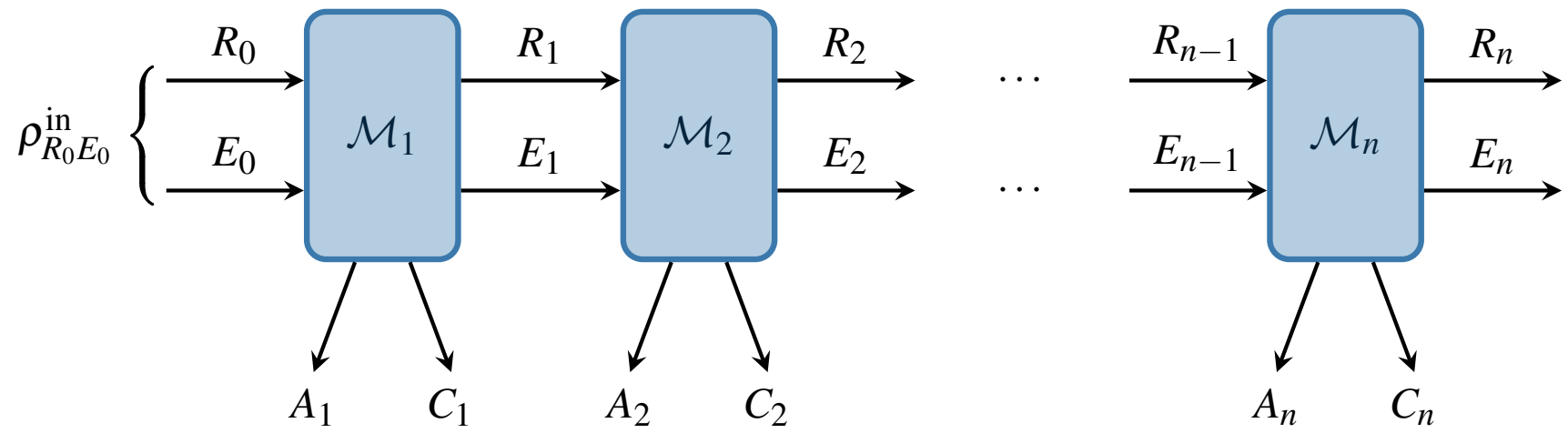

FIG. S6. Setup of the generalized entropy accumulation theorem (GEAT). A sequence of channels $\mathcal{M}_1,\ldots,\mathcal{M}_n$ are applied to some initial state $\rho^{\rm in}_{R_0E_0}$. The register R_i is the memory of the device in round i and the register E_i is the adversary's information in round i. Assuming that $\mathcal{M}_i$ does not signal from R_{i-1} to E_i (see Definition 5), the GEAT allows us to bound the entropy of $\mathbf{A} = A_1\ldots A_n$ relative to the adversary's final side information E_n. The registers C_i are used for collecting statistics about the behavior of the channels $\mathcal{M}_i$ (see Definition 6).

The bound on $H^{\varepsilon}_{\min}(\mathbf{A}|E_n)$ depends on the quantities of the chosen min-tradeoff function given in Definition 9 below.

Definition 9 (Min, Max, Var)**.** *Let $f:\mathbb{P}_{\mathcal{C}}\to\mathbb{R}$ be an affine function. Define*

$$\begin{aligned}
\mathrm{Max}(f) &:= \max_{q\in\mathbb{P}_{\mathcal{C}}} f(q),\\
\mathrm{Min}(f) &:= \min_{q\in\mathbb{P}_{\mathcal{C}}} f(q),\\
\mathrm{Min}_{\Sigma}(f) &:= \min_{q:\Sigma(q)\neq\emptyset} f(q),\\
\mathrm{Var}_{\Sigma}(f) &:= \max_{q:\Sigma(q)\neq\emptyset}\sum_{c\in\mathcal{C}} q(c)\left(f(\delta_c)-\sum_{c'\in\mathcal{C}} q(c')f(\delta_{c'})\right)^2,
\end{aligned} \tag{27}$$

where $\Sigma(q)=\cup_i\Sigma_i(q)$ and δ_c is the distribution with all the weight on c.

We now have all the preliminary definitions in order to formally state the GEAT.

Theorem 2 (Generalised entropy accumulation theorem (GEAT) [37, Theorem 4.3])**.** *Let $\mathcal{M}_1,\ldots,\mathcal{M}_n$ be a sequence of GEAT channels, $\rho^{\rm in}_{R_0E_0}$ be an arbitrary quantum state, $\Omega\subseteq\mathcal{C}^n$, $\alpha\in(1,3/2)$, and $\varepsilon_s\in(0,1)$. Define $\rho^{\rm out}_{\mathbf{AC}R_nE_n} := (\mathcal{M}_n\circ\ldots\circ\mathcal{M}_1)[\rho^{\rm in}]$. Then, for any min-tradeoff function f,*

$$\begin{aligned}
H^{\varepsilon_s}_{\min}(\mathbf{A}|E_n)_{\rho^{\rm out}_{|\Omega}} \geq & nh - n\frac{\alpha-1}{2-\alpha}\frac{\ln 2}{2}V^2 - \frac{g(\varepsilon_s)-\alpha\log_2\Pr[\Omega]}{\alpha-1}\\
& - n\left(\frac{\alpha-1}{2-\alpha}\right)^2 K_\alpha,
\end{aligned} \tag{28}$$

where

$$\begin{aligned}
h &= \min_{\mathbf{c}\in\Omega} f(\mathrm{freq}_{\mathbf{c}})\\
V &= \log_2(2d_A^2+1)+\sqrt{2+\mathrm{Var}(f)}\\
K_\alpha &= \frac{(2-\alpha)^3}{6(3-2\alpha)^3\ln 2}2^{\frac{\alpha-1}{2-\alpha}(2\log_2 d_A+\mathrm{Max}(f)-\mathrm{Min}_{\Sigma}(f))}\\
&\quad\cdot\ln^3\left(d_A^2 2^{\mathrm{Max}(f)-\mathrm{Min}_{\Sigma}(f)}+e^2\right)\\
g(\varepsilon_s) &= -\log_2(1-\sqrt{1-\varepsilon_s^2}).
\end{aligned} \tag{29}$$

We can now phrase our protocol as a sequence of GEAT channels. For this, we define the channel $\mathcal{M}_i$ to be the channel which

1. Draws two bits $X_i\in\{0,1\}$ and $Y_i\in\{0,1\}$ from each of the two quantum SV sources,
2. inputs X_i and Y_i into the device λ, and
3. records the outputs $A_i, B_i\in\{0,1\}$.

Due to the assumption that the source is isolated from the device λ (see Section V), the channels satisfy the non-signalling condition of the GEAT. Hence we can apply the GEAT to bound the entropy of $\mathbf{AB}$ in terms of single-round entropies (quantified by the min-tradeoff function). A diagrammatic illustration of the setup is given in Fig. S7.

The remaining challenge is to find an appropriate min-tradeoff function. This requires finding a bound on the single-round von Neumann entropy, as discussed in the following section.

Single-round entropy bound

In this section we derive a bound on the single-round von Neumann entropy. For ease of notation, we will suppress dependencies on the round index i. To compute the single-round entropy, we will consider the following situation (see also Fig. S7): We are given some initial state $\rho_{R_AR_BQE}$, where $R_AR_B = R$ denote the memory of Alice and Bob's devices, Q is the memory of the quantum SV source, and E is the adversary's side information. Next, we draw two bits X and Y from the two quantum SV sources, which, by Lemma 2, can be modeled as a single MDL source $\mathcal{S}_{XYQ'|Q}$. Finally, depending on the measurement settings X and Y, the devices perform measurements $M_{R_A}(a|x)$ and $N_{R_B}(b|y)$ to produce random variables A and B.

The strategy for deriving the single-round entropy bound is similar to [11]. That is, we use the observed MDL violation to find a bound on the CHSH violation which allows us to bound the von Neumann entropy. For this, we first introduce the following notation for the CHSH and MDL values.

Definition 10. *Let $\{M_{R_A}(a|x)\}$ and $\{N_{R_B}(b|y)\}$ be two conditional POVM elements. Given a quantum state $\rho_{R_AR_B}$, we*

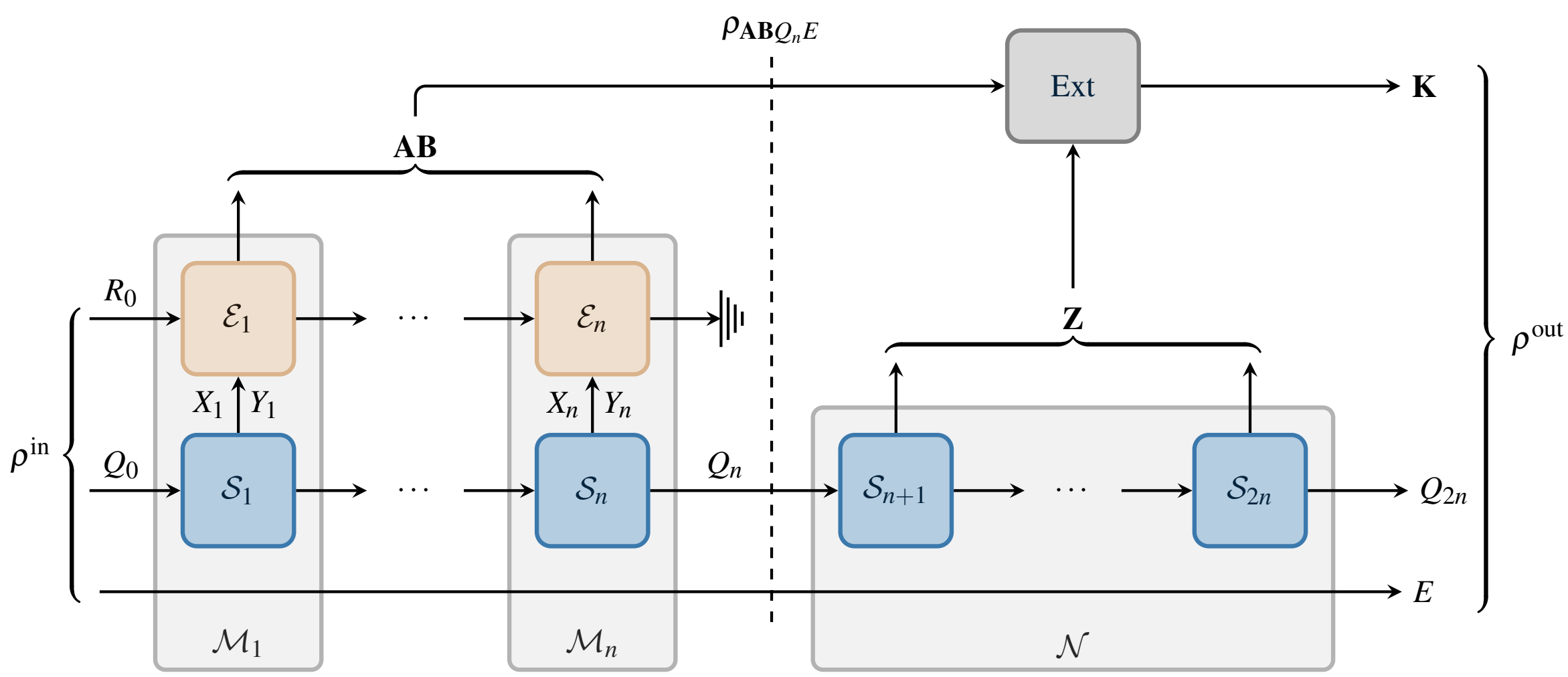

FIG. S7. Diagrammatic representation of the device-independent randomness amplification protocol in Box I. Both quantum SV sources are used n times to produce inputs $X_1 \dots X_n$ and $Y_1 \dots Y_n$ respectively. The device λ (represented by the brown boxes) then, depending on the inputs X_i and Y_i, produces output bits A_i and B_i respectively. Afterwards, one of the two SV sources is used another $2n$ times to produce the seed $\mathbf{Z}$ which is, together with $\mathbf{AB}$, fed into the extractor Ext to produce the final random bitstring $\mathbf{K}$.

define the CHSH value in Eberhard form *[38] of ρ as*

$$\begin{aligned}\alpha(\rho_{R_AR_B}) := &\operatorname{tr}[M_{R_A}(0|0)\otimes N_{R_B}(0|0)\rho_{R_AR_B}]\\ &-\operatorname{tr}[M_{R_A}(0|0)\otimes N_{R_B}(1|1)\rho_{R_AR_B}]\\ &-\operatorname{tr}[M_{R_A}(1|1)\otimes N_{R_B}(0|0)\rho_{R_AR_B}]\\ &-\operatorname{tr}[M_{R_A}(0|1)\otimes N_{R_B}(0|1)\rho_{R_AR_B}].\end{aligned} \tag{30}$$

Given a quantum state $\rho_{R_AR_BXY}$ with X and Y two classical bits, we define the MDL value *of ρ by*

$$S^{\mu_{\min},\mu_{\max}}(\rho_{R_AR_BXY}) \tag{31}$$

$$\begin{aligned}:=&\mu_{\min}\operatorname{tr}[M_{R_A}(0|0)\otimes N_{R_B}(0|0)\rho_{R_AR_B\wedge 00}]\\ &-\mu_{\max}\big(\operatorname{tr}[M_{R_A}(0|0)\otimes N_{R_B}(1|1)\rho_{R_AR_B\wedge 01}]\\ &\quad+\operatorname{tr}[M_{R_A}(1|1)\otimes N_{R_B}(0|0)\rho_{R_AR_B\wedge 10}]\\ &\quad+\operatorname{tr}[M_{R_A}(0|1)\otimes N_{R_B}(0|1)\rho_{R_AR_B\wedge 11}]\big).\end{aligned} \tag{32}$$

We can write the more commonly used CHSH value S as $S = 4\alpha + 2$ [11]. Hence, local hidden variable models satisfy $\alpha(\rho_{R_AR_B}) \le 0$ [39]. Similarly, local hidden variable models satisfy $S^{\mu_{\min},\mu_{\max}}(\rho_{R_AR_BXY}) \le 0$ for all states $\rho_{R_AR_BXY}$ produced by a $(\mu_{\min},\mu_{\max})$-MDL source [30].

The following lemma shows that if the initial state $\rho_{R_AR_BQE}$ and the measurements $M_{R_A}(a|x), N_{R_B}(b|y)$ are such that they achieve a given MDL violation S_μ, then the same state and measurements also violate the CHSH inequality.

Lemma 4. *Let $\omega_{R_AR_BQE}$ be an arbitrary state and $\mathcal{S}_{XYQ'|Q}$ be a quantum $(\mu_{\min},\mu_{\max})$-MDL source. Assume that the state $\rho_{R_AR_BXYQ'W} := \mathcal{S}_{XYQ'|Q}[\omega_{R_AR_BQE}]$ satisfies $S^{\mu_{\min},\mu_{\max}}(\rho_{R_AR_BXY}) \ge S_\mu$, then*

$$\alpha(\omega_{R_AR_B}) \ge \frac{1}{\mu_{\min}\mu_{\max}} S_\mu. \tag{33}$$

Proof. Since, by assumption, $\mathcal{S}_{XYQ'|Q}$ is a quantum $(\mu_{\min},\mu_{\max})$-MDL source, we know that

$$\rho_{R_AR_BE\wedge xy} \le \mu_{\max}\omega_{R_AR_BE} \quad \text{and} \quad \rho_{R_AR_BE\wedge xy} \ge \mu_{\min}\omega_{R_AR_BE} \tag{34}$$

hold for all x and y.We can now bound

$$\begin{aligned}S_\mu \le& S^{\mu_{\min},\mu_{\max}}(\rho_{R_AR_BXY}) &(35)\\ =&\mu_{\min}\operatorname{tr}[M_{R_A}(0|0)\otimes N_{R_B}(0|0)\rho_{R_AR_B\wedge 00}] &(36)\\ &-\mu_{\max}\big(\operatorname{tr}[M_{R_A}(0|0)\otimes N_{R_B}(1|1)\rho_{R_AR_B\wedge 01}]\\ &\quad+\operatorname{tr}[M_{R_A}(1|1)\otimes N_B(0|0)\rho_{R_AR_B\wedge 10}]\\ &\quad+\operatorname{tr}[M_{R_A}(0|1)\otimes N_{R_B}(0|1)\rho_{R_AR_B\wedge 11}]\big)\\ \le&\mu_{\min}\mu_{\max}\Big(\operatorname{tr}[M_{R_A}(0|0)\otimes N_{R_B}(0|0)\omega_{R_AR_B}] &(37)\\ &\quad-\operatorname{tr}[M_{R_A}(0|0)\otimes N_{R_B}(1|1)\omega_{R_AR_B}]\\ &\quad-\operatorname{tr}[M_{R_A}(1|1)\otimes N_{R_B}(0|0)\omega_{R_AR_B}]\\ &\quad-\operatorname{tr}[M_{R_A}(0|1)\otimes N_{R_B}(0|1)\omega_{R_AR_B}]\Big)\\ =&\mu_{\min}\mu_{\max}\alpha(\omega_{R_AR_B}), &(38)\end{aligned}$$

where we used (34) for the second inequality. □

To bound the von Neumann entropy, we will use the following result from [40].

Lemma 5 ([40]). *Let $\rho_{R_AR_BE}$ be a state and write $\alpha \equiv \alpha(\rho_{R_AR_B})$. Let $\mathcal{M}^x_{A|R_A}$ denote the channel performing the measurement $\{M_{R_A}(a|x)\}_a$. Then*

$$H(A|E)_{\mathcal{M}^x[\rho]} \ge \begin{cases}1 - h_2\left(\frac{1}{2} + \sqrt{\alpha(\alpha+1)}\right) & \text{if } \alpha \ge 0\\ 0 & \text{otherwise}\end{cases} =: h(\alpha), \tag{39}$$

holds for all $x \in \{0,1\}$. Furthermore, $h(\alpha)$ is monotonically increasing and convex in α.

The following lemma bounds the von Neumann entropy of states which violate a $\mu-$MDL inequality.

Lemma 6. *Let $\mathcal{S}_{XYQ'|Q}$ be a quantum $(\mu_{\min},\mu_{\max})$-MDL source and $\omega_{R_A R_B QE}$ be a state such that $\rho_{R_A R_B XYQ'E} := \mathcal{S}_{XYQ'|Q}[\omega_{R_A R_B QE}]$ satisfies $S^{\mu_{\min},\mu_{\max}}(\rho_{R_A R_B XY}) \geq S_\mu$. Let $\mathcal{M}_{AX|R_A X}$ denote the channel which first reads the register X and then performs the measurement $\{M_{R_A}(a|x)\}$. Then*

$$H(A|XYQ'E)_{\mathcal{M}[\rho]} \geq h(\alpha^*), \tag{40}$$

where $\alpha^* := \frac{1}{\mu_{min}\mu_{\max}} S_\mu$ and h is the function introduced in Lemma 5.

Proof. Let us write

$$\rho_{R_A R_B XYQ'E} = \sum_{x,y} p(x,y)\, |x,y\rangle\langle x,y|_{XY} \otimes \rho_{R_A R_B Q'E|xy}. \tag{41}$$

For ease of notation, introduce $\alpha_{xy} := \alpha(\rho_{R_A R_B|xy})$. Note that

$$\begin{aligned}\sum_{x,y} p(x,y)\alpha_{xy} =& \alpha\left(\sum_{x,y} p(x,y)\rho_{R_A R_B|xy}\right)\\ =&\alpha(\rho_{R_A R_B})\\ =&\alpha(\omega_{R_A R_B}),\end{aligned} \tag{42}$$

by the linearity of $\alpha(\rho)$. We have

$$\begin{aligned}H(A|XYQ'E)_{\mathcal{M}[\rho]} =& \sum_{x,y} p(x,y) H(A|Q'E)_{\mathcal{M}[\rho_{|xy}]}\\ \geq& \sum_{x,y} p(x,y) h(\alpha_{xy})\\ \geq& h\left(\sum_{x,y} p(x,y)\alpha_{xy}\right)\\ =& h(\alpha(\omega_{R_A R_B})),\end{aligned} \tag{43}$$

where for the first inequality we applied Lemma 5 and for the second inequality, we used the convexity of h. Now, by Lemma 4, we have $\alpha(\omega_{R_A R_B}) \geq \frac{1}{\mu_{\min}\mu_{\max}} S_\mu = \alpha^*$ and the lemma follows by noting that $h(\alpha)$ is monotonically increasing in α. □

Security proof

In this subsection we combine the previous results to show the final security statement.

Theorem 3. *Let ρ^{out} be the state generated at the end of the protocol using the DEOR$^{\mathcal{K}}$ extractor with non-cyclic right-shift matrices. Furthermore, let $\varepsilon, \varepsilon_{\text{EA}}, \varepsilon_s \in (0,1)$ be arbitrary, $S_\mu \in \mathbb{R}$, and the output size $m \in \mathbb{N}$ be chosen such that*

$$\begin{aligned}m \leq& \frac{n}{3}\left(\eta(\varepsilon_s, \varepsilon_{\text{EA}}, S_\mu, n) + 2\log_2 \frac{1}{\frac{1}{2}+\mu} - 2\right)\\ &- \frac{2}{3}\log_2 \frac{1}{2\varepsilon - 4\varepsilon_s},\end{aligned} \tag{44}$$

where the function η is given in (50) below. Let Ω be the event that $S_{\mu,\text{obs}} \geq S_\mu$. Then, under the assumptions outlined in section V,

$$\Pr[\Omega]\frac{1}{2}\|\rho^{\text{out}}_{\mathbf{KZ}Q_{2n}E|\Omega} - \omega_{\mathbf{K}} \otimes \rho^{\text{out}}_{\mathbf{Z}Q_{2n}E|\Omega}\|_1 \leq \max\{\varepsilon_{\text{EA}}, \varepsilon\}. \tag{45}$$

Proof. We note that if $\Pr[\Omega] < \varepsilon_{\text{EA}}$, then (45) is immediately satisfied. Hence it suffices to consider the case $\Pr[\Omega] \geq \varepsilon_{\text{EA}}$.

Let the channels $\mathcal{M}_i$ be as drawn in Fig. S7. It is clear that $\mathcal{M}_i$ does not signal from $R_{i-1} := R_{A,i-1}R_{B,i-1}$ to $Q_i E =: E_i$. Furthermore, let the testing register C_i be computed as

$$C_i = \begin{cases} \text{“}\mu_{\min}\text{”} & \text{if } (A_i,B_i,X_i,Y_i) = (0,0,0,0)\\ \text{“}\mu_{\max}\text{”} & \text{if } (A_i,B_i,X_i,Y_i) \in \{(0,1,0,1),(1,0,1,0),\\ & \qquad (0,0,1,1)\}\\ \perp & \text{otherwise.}\end{cases} \tag{46}$$

In particular, we choose the alphabet $\mathcal{C}$ as $\mathcal{C} = \{\text{“}\mu_{\min}\text{”}, \text{“}\mu_{\max}\text{”}, \perp\}$. We can, without loss of generality, assume that Q_i (and hence E_i) contains a copy of X_i and Y_i. Then, C_i can be computed from $A_i B_i$ and E_i as in (23).

The next step is to compute a min-tradeoff function for $\mathcal{M}_i$. For this, let us define the function $S_\mu : \mathbb{P}_{\mathcal{C}} \to \mathbb{R}$ as

$$S_\mu(q) := \mu_{\min} q(\text{“}\mu_{\min}\text{”}) - \mu_{\max} q(\text{“}\mu_{\max}\text{”}), \tag{47}$$

that is, S_μ calculates the MDL value given a distribution $q \in \mathbb{P}_{\mathcal{C}}$. Now, by Lemma 6, we have for all $\nu_{A_i C_i R_i E_i \tilde{E}_{i-1}} \in \Sigma_i$ that

$$\begin{aligned}H(A_i B_i|E_i\tilde{E}_{i-1})_\nu \geq& H(A_i|E_i\tilde{E}_{i-1})_\nu\\ \geq& h\left(\frac{S_\mu(\nu_{C_i})}{1/4-\mu^2}\right)\\ =:& H(S_\mu),\end{aligned} \tag{48}$$

where we used that the entropy can only decrease when discarding the classical information B_i and noted that $\mu_{\min}\mu_{\max} = \left(\frac{1}{2}-\mu\right)\left(\frac{1}{2}+\mu\right) = \frac{1}{4}-\mu^2$. Hence, it suffices to find a min-tradeoff function for $H(S_\mu)$. For any $s \in \mathbb{R}$, we define the affine function

$$f^s_{\min}(q) = g(s) + (D_s H)(S_\mu(q) - s), \tag{49}$$

where $D_s H$ denotes the derivative of H at the point $S_\mu = s$. That is, $f^s_{\min}$ is the linearization of H around the point $S_\mu = s$. Note that, by the convexity of H, $f^s_{\min}(q) \leq H(S_\mu(q))$ and hence $f^s_{\min}$ is a valid min-tradeoff function (for any s).

Let $\rho_{\mathbf{ABC}Q_n E}$ be the state after step 1 of the protocol in Box I (see also Fig. S7). We can now apply the GEAT from Theorem 2 to obtain

$$H^{\varepsilon_s}_{\min}(\mathbf{AB}|Q_n E)_{\rho_{|\Omega}} \geq n\eta(\varepsilon_s, \varepsilon_{\text{EA}}, S_\mu, n), \tag{50}$$

where $n\eta$ is the RHS of (28) evaluated for the min-tradeoff function $f^s_{\min}$, $\Pr[\Omega] = \varepsilon_{\text{EA}}$, and $h = f^s_{\min}(S_\mu)$. In particular, this means that there exists a state $\tilde{\rho}_{\mathbf{AB}Q_n E}$ which is ε_s close in purified distance to $\rho_{\mathbf{AB}Q_n E|\Omega}$ and satisfies $H_{\min}(\mathbf{AB}|Q_n E)_{\tilde{\rho}} \geq n\eta$.

The last step is to apply a two-process extractor to extract the final uniformly random bitstring $\mathbf{K}$. For this, let us denote by $\mathcal{N}_{\mathbf{Z}Q_{2n}|Q_n}$ the channel which draws another $2n$ bits $\mathbf{Z}$ from one of the two SV sources (see also Fig. S7). By Lemma 1, we have that

$$H_{\min}(\mathbf{Z}|\tilde{Q})_{\mathcal{N}[\sigma]} \geq -2n\log_2\left(\frac{1}{2}+\mu\right) =: 2nk_2, \tag{51}$$

for any $\sigma_{Q_n\tilde{Q}}$. Applying Lemma 3 and Theorem 1 to $\text{DEOR}^{\mathcal{K}}$, $\tilde{\rho}_{\mathbf{AB}Q_nE}$ and $\mathcal{N}_{\mathbf{Z}Q_{2n}|Q_n}$ gives

$$\frac{1}{2}\left\|\tilde{\rho}_{\mathbf{KZ}Q_{2n}E} - \omega_{\mathbf{K}}\otimes\tilde{\rho}_{\mathbf{Z}Q_{2n}E}\right\|_1 \leq \frac{1}{2}\sqrt{2^{3m+2n-n\eta-2nk_2}}, \tag{52}$$

which, by the triangle inequality, yields

$$\begin{aligned} &\frac{1}{2}\left\|\rho^{\text{out}}_{\mathbf{KZ}Q_{2n}E|\Omega} - \omega_{\mathbf{K}}\otimes\rho^{\text{out}}_{\mathbf{Z}Q_{2n}E|\Omega}\right\|_1 \\ \leq&\frac{1}{2}\left\|\rho^{\text{out}}_{\mathbf{KZ}Q_{2n}E|\Omega} + \tilde{\rho}_{\mathbf{KZ}Q_{2n}E}\right\|_1 \\ &+\frac{1}{2}\left\|\tilde{\rho}_{\mathbf{KZ}Q_{2n}E} - \omega_{\mathbf{K}}\otimes\tilde{\rho}_{\mathbf{Z}Q_{2n}E}\right\|_1 \\ &+\frac{1}{2}\left\|\tilde{\rho}_{\mathbf{KZ}Q_{2n}E} - \omega_{\mathbf{K}}\otimes\rho^{\text{out}}_{\mathbf{Z}Q_{2n}E|\Omega}\right\|_1 \\ \leq&2\varepsilon_s + \frac{1}{2}\sqrt{2^{3m+2n-n\eta-2nk_2}}. \end{aligned} \tag{53}$$

By the condition in (44), this is less than ε. □

Note that Theorem 3 implies that asymptotically one can produce randomness (i.e. $m > 0$) if

$$h\left(\frac{S_\mu}{1/4-\mu^2}\right) + 2\log_2\frac{1}{\frac{1}{2}+\mu} - 2 > 0. \tag{54}$$

Since the LHS of (54) decreases for increasing bias μ, there is a maximum bias which can be tolerated, see Fig. 2 in the main text. The constraint in (54) arises from the extractor constraint in Theorem 1 which requires that $k_1 + k_2 > n$. Hence, finding extractors with weaker requirements would allow us to improve the maximum bias μ which can be tolerated (see also Section VIII). While such extractors are known to exists for (conditionally) independent quantum states [41] (some of which even admit efficient implementations [33, 42]), it is currently unknown whether they remain secure as two-process extractors. We consider this an important open problem.

The previous section assumed that the key length is fixed before the protocol begins. In order to maximize the amount of randomness that can be extracted, one would like to adjust the output size depending on the observed violation of the MDL-inequality (compare Box I). This can be done using the following theorem.

Theorem 4. *Let $\varepsilon', \varepsilon_s \in (0,1)$ be arbitrary. Let $S_{\mu,\max} \in \mathbb{R}$, $M \in \mathbb{N}$ and set $\Delta S_\mu = S_{\mu,\max}/M$. Choose the output size as*

$$\begin{aligned} m'(S_{\mu,\text{obs}}) =&\frac{n}{3}\left(\eta\left(\varepsilon_s,\varepsilon',\min\left\{S_{\mu,\max},\left\lfloor\frac{S_{\mu,\text{obs}}}{\Delta S_\mu}\right\rfloor\Delta S_\mu\right\},n\right)\right. \\ &\left. + 2\log_2\frac{1}{\frac{1}{2}+\mu} - 2\right) - \frac{2}{3}\log_2\frac{1}{2\varepsilon' - 4\varepsilon_s} \\ m(S_{\mu,\text{obs}}) =&\max\{\lfloor m'(S_{\mu,\text{obs}})\rfloor, 0\}. \end{aligned} \tag{55}$$

Then, under the assumptions in Section V, the protocol is $M\varepsilon'$ secure when using the $\text{DEOR}^{\mathcal{K}}$ extractor with non-cyclic right-shift matrices.

Proof. For $i \in \{1,\dots,M-1\}$, define Ω_i as the event when the observed MDL violation satisfies $i\Delta S_\mu \leq S_{\mu,\text{obs}} < (i+1)\Delta S_\mu$, Ω_0 as the event when $S_{\mu,\text{obs}} < \Delta S_\mu$, and Ω_M as the event when $S_{\mu,\text{obs}} \geq S_{\mu,\max}$. Define $m_i = m(i\Delta S_\mu)$, then the security parameter can be bounded by

$$\sum_{i=0}^{M}\Pr[\Omega_i]\frac{1}{2}\|\rho_{\mathbf{KY}Q_{2n}E|\Omega_i} - \omega_{m_i}\otimes\rho_{\mathbf{Y}Q_{2n}E|\Omega_i}\|_1 \leq M\varepsilon', \tag{56}$$

where we used that for $i = 0$ the trace distance vanishes (because $m_0 = 0$ and hence $\mathbf{K}$ is trivial) and used Theorem 3 (with $\varepsilon_{\text{EA}} = \varepsilon'$) to bound the terms for $i = 1,\dots,M$ by ε. □

Possible improvements

While the security analysis presented here is sufficient to experimentally demonstrate randomness amplification, there are a number of ways in which our bounds might be tightened. Firstly, in bounding the randomness of the Bell test outputs $\mathbf{AB}$, we have applied the simple bound

$$H(A_iB_i|E_i\tilde{E}_{i-1}) \geq H(A_i|E_i\tilde{E}_{i-1}), \tag{57}$$

see (48). This was done because analytical bounds on $H(A_i|E_i\tilde{E}_{i-1})$ can then be derived. This may be improved by applying numerical methods that are able to bound $H(A_iB_i|E_i\tilde{E}_{i-1})$ directly [43]. Secondly, to bound the entropy of the $\mu - SV$ source, we applied the simple bound

$$H_{\min}(\mathbf{Z}|\tilde{Q}) \geq -2n\log_2\left(\frac{1}{2}+\mu\right), \tag{58}$$

see (51). Here, we would expect to be able to apply the GEAT to improve this to the stronger bound

$$H^{\varepsilon}_{\min}(\mathbf{Z}|\tilde{Q}) \geq 2nh_2\left(\frac{1}{2}+\mu\right), \tag{59}$$

where $h_2(p) := -p\log_2 p - (1-p)\log_2(1-p)$ denotes the binary entropy. Finally, it is unclear whether the MDL-inequality is optimal for the task of randomness amplification. For instance, in [16], they show that tripartite Bell inequalities (more precisely, the Mermin inequality) can give improvements to the tolerated bias μ. It is therefore conceivable that even for the bipartite setting considered here, there may be better Bell inequalities.

Choice of protocol parameters

The main security statement is given in Theorem 4. In Theorem 4 we have the freedom to pick the various parameters ($\varepsilon', \varepsilon_s, M$, etc.). We summarize our choices in Table S4 below.

VII. EXTRACTED RANDOM BITS

Fig. S8 shows the main output of this experiment: the $45,025,658$ ε-random bits (with $\varepsilon = 10^{-12}$) produced via device-independent randomness amplification. Each pixel represents a single extracted bit. The randomness extraction is implemented using the Julia programming language. To achieve a $O(n \log n)$ runtime, following [16], we implement the DEOR$^{\mathcal{K}}$ extractor with non-cyclic right-shift matrices using the number theoretic transform. The code was run on ETH Zurich's Euler cluster, taking roughly 2 hours on a single core of AMD Epyc CPU while consuming roughly 81GB of memory.

Furthermore, Fig. S9 provides a visual demonstration of randomness amplification: the same image (left) is encrypted with a one-time pad generated from the protocol's output bits (middle), and encrypted with a one-time pad generated from weak random bits with bias equal to the maximum tolerated input bias of the protocol (right). The example illustrates the importance of high-quality randomness for cryptographic security.

Symbol	Quantity	Value
μ	Maximum tolerated bias	0.75%
M	Number of different output lengths	10^3
$S_{\mu,\max}$	Upper bound on MDL violation	$\frac{\sqrt{2}-1}{2}(\frac{1}{4}-\mu^2)^2 \approx 0.01294$
ε'	Fixed-length security parameter	10^{-15}
ε_s	Smoothing parameter	10^{-16}
ε_{EA}	Threshold abort probability	10^{-15}
α	Rényi parameter, optimized numerically	$1+4.775\cdot 10^{-5}$
s	Footpoint of min-tradeoff function, optimized numerically	$2.912\cdot 10^{-3}$
n	Number of protocol rounds	$20\cdot 2^{26}$
d	Seed length of extractor	$40\cdot 2^{26}$
Ext	The two-process extractor	Dodis et al.'s extractor DEOR$^{\mathcal{K}}$ using non-cyclic right shifts. See Definition 4
m	Output length, chosen according to Theorem 4	$m = 45,025,658$

TABLE S4. Choices for parameters introduced in Box I and Theorem 4. Note that the parameters are chosen such that, according to Theorem 4, the overall security parameter is $M\varepsilon' = 10^{-12}$, as claimed in the main text.

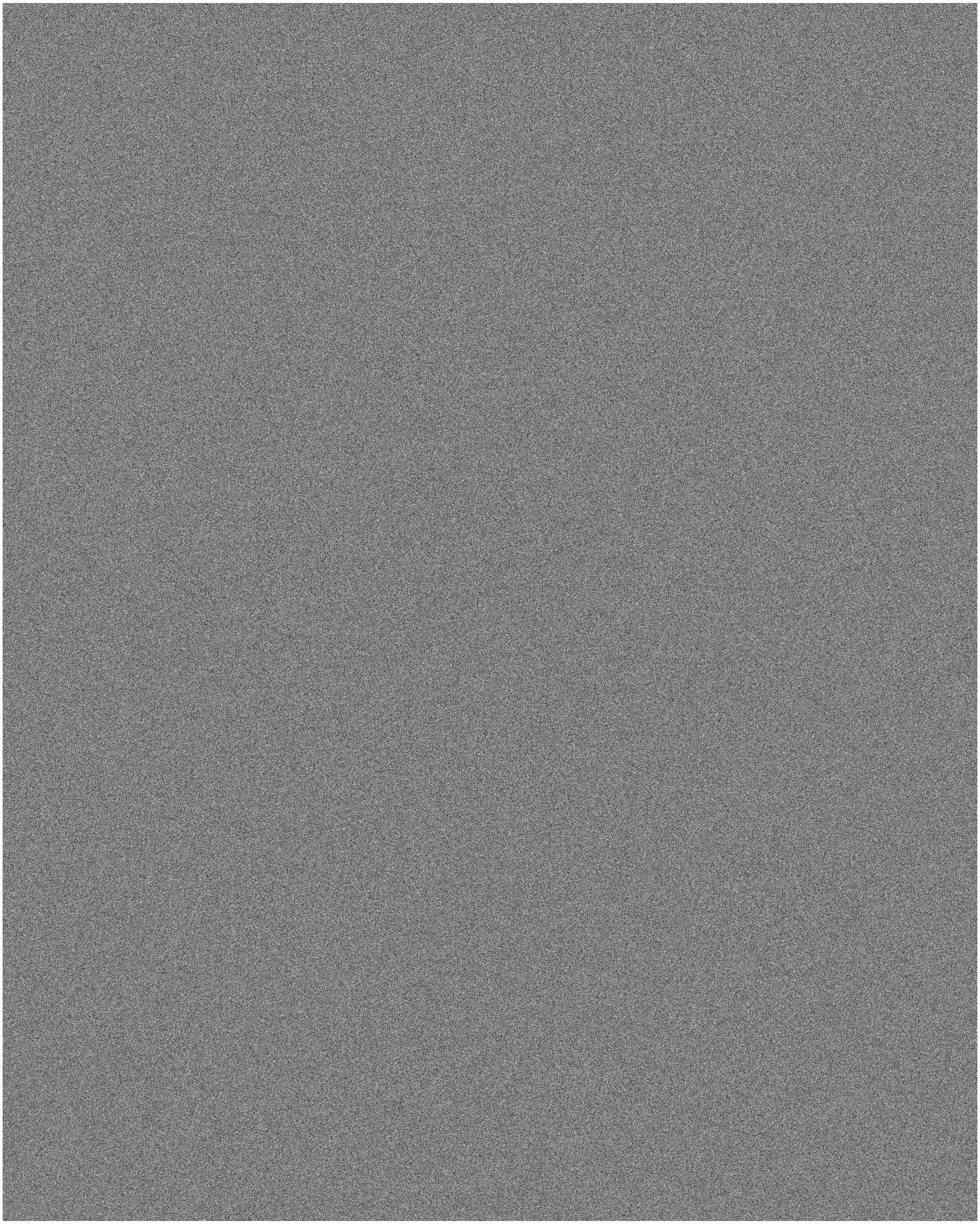

FIG. S8. The first $45,000,000$ random bits produced by device-independent randomness amplification. The bits are arranged row by row from left to right, and from top to bottom. A white pixel represents a "1", a black pixel marks a "0".

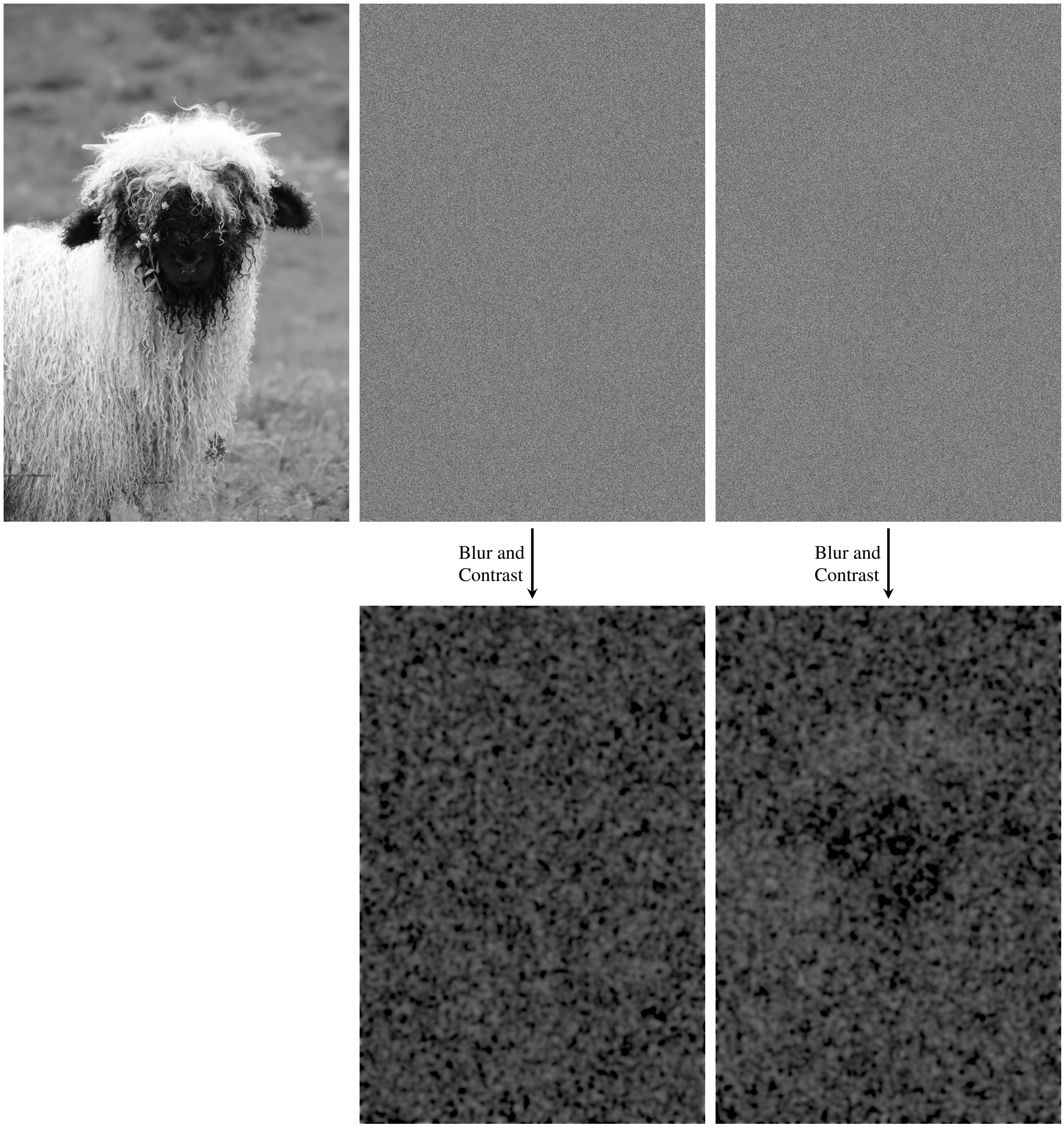

FIG. S9. Photo of a Valais Blacknose sheep (top left) and the same image encrypted with a one-time pad using our ε-random output bits (top center), and with imperfect randomness with bias $\mu = 0.75\%$ (top right). The bottom row shows the corresponding images after simple image processing (Gaussian blur and increased contrast). In contrast to encryption with our high-quality output randomness, encryption using biased randomness leaks information, revealing the presence of the sheep (note that, conversely, the invisibility of the sheep on the bottom center does, by itself, not prove the cryptographic security of our random bits). This illustrates how even a small bias can render randomness unsuitable for cryptographic applications. Although each individual bit of a μ-SV source may be close to uniform, the bits collectively need not be. In contrast, the $45,025,658$ output bits produced by our protocol have a total error of less than $\varepsilon = 10^{-12}$.

VIII. TRADEOFF BETWEEN PROTOCOL PARAMETERS

There are various protocol parameters which need to be chosen (see Box I). The most relevant parameters are the number of Bell-test trials n, the assumed bias μ, the desired security parameter ε, the expected μ-MDL value S_μ, and the number of output bits m. Out of these parameters, ε, μ, and m can be chosen freely, whereas n and S_μ are fixed by experimental constraints. Furthermore, these parameters are not independent. As shown in Theorem 3, for fixed n, μ, ε, and S_μ, there is a maximum m which can be chosen.

Since randomness is usually a resource in a larger task, the security parameter ε and the number of random bits m are fixed by the task. Furthermore, the number of trials n is limited by the repetition rate of the experiment and the time available for executing it. By Theorem 3, there is then a minimal required μ-MDL value S_μ for which the desired parameters of ε, m, and n are possible (at some fixed μ). Given that μ represents an assumption about the predictability of the SV sources, we would like to pick μ as large as possible. Note that this largest value of μ for which the choices ε, m, and n are possible depends on the precise relationship between μ and S_μ and hence on the experimentally achievable parameters.

Next, we would like to understand how the largest feasible bias μ changes under different experimental conditions. For this, we will consider a simple depolarizing noise model. More precisely, we consider states of the form

$$|\psi\rangle_{AB} = \cos\alpha\,|00\rangle_{AB} + \sin\alpha\,|11\rangle_{AB} \tag{60}$$

subject to depolarizing noise

$$\rho_{AB} := (1-q)\,|\psi\rangle\langle\psi|_{AB} + q\frac{1}{4}\mathbb{1}_{AB}, \tag{61}$$

where q denotes the depolarizing probability. Furthermore, we model the measurements as the POVMs

$$\begin{aligned} M(a|x) &= \frac{1}{2}\Big(\mathbb{1} + (-1)^a(\cos\theta_x\sigma_x + \sin\theta_x\sigma_z)\Big), \\ N(b|y) &= \frac{1}{2}\Big(\mathbb{1} + (-1)^b(\cos\phi_y\sigma_x + \sin\phi_y\sigma_z)\Big), \end{aligned} \tag{62}$$

where $x,y,a,b \in \{0,1\}$ and $\sigma_{x,z}$ are the Pauli matrices. As there are a number of experimental parameters impacting the performance of our experiment, one should not think of the above model as giving a detailed description of the states and measurements implemented in our experiment. Rather, depolarizing noise is used as a simplified model to describe the collective effect of many sources of imperfections. Nevertheless, we found the above model to describe our experiment quite well.

In (60) and (62), the parameters α, θ_x, and ϕ_y can be freely optimized in order to maximize the violation S_μ of the μ-MDL inequality (for some given μ). A special case of this is the CHSH inequality which corresponds to $\mu = 0$. For the CHSH inequality, the optimal angles are given by $\alpha = \frac{\pi}{4}, \theta_0 = 0, \theta_1 = \frac{\pi}{2}, \phi_0 = \frac{\pi}{4}$, and $\phi_1 = -\frac{\pi}{4}$ yielding a CHSH value $S = 2\sqrt{2}(1-q)$.

Theorem 3 establishes the tradeoff between the required violation S_μ of the μ-MDL inequality and the number of trials n (while taking the output size m, the bias μ, and the errors $\varepsilon, \varepsilon_s, \varepsilon_{\mathrm{EA}}$ to be fixed). Given the depolarizing noise model above, one can directly compute S_μ as a function of the depolarizing probability q (while optimizing the angles α, θ_x, ϕ_y) which in turn can be parametrized by the CHSH value S. Hence, for fixed bias μ (and fixed m, $\varepsilon, \varepsilon_s$, and $\varepsilon_{\mathrm{EA}}$), we get a relation between the CHSH value S and the required number of trials n. Computing this tradeoff for different biases μ gives the relationship between S, n, and μ which is shown in Fig. 2 of the main text. We remark that this tradeoff depends on the assumed model for experimental imperfections (i.e., depolarizing noise in our example). However, since for $\mu = 0$ the μ-MDL inequality reduces to the CHSH inequality, the contour line for $\mu = 0$ in Fig. 2 of the main text is independent of the assumed noise model. This means that whether or not an experiment is sufficient for randomness amplification with very small bias $\mu > 0$ only depends on the reported CHSH violation S and the number of trials n. However, once one crosses the line at $\mu = 0$, the noise model will impact the maximum bias μ which can be tolerated. Since all prior experiments shown in Fig. 2 of the main text are below (or very close to) the $\mu = 0$ contour line, we can conclude that they would be insufficient for randomness amplification with significant bias $\mu > 0$.

Finally, we would like to understand the maximal bias μ which could, in principle, be tolerated by our protocol. As shown in Fig. 2 in the main text, using ideal devices (i.e. $S = 2\sqrt{2}$), we can tolerate a bias μ slightly above 4%. This is entirely a consequence of the large entropy requirements of our extractor (the extractor requires that $H_{\min}^{\varepsilon_s}(\mathbf{AB}|E_n) + H_{\min}(\mathbf{Z}|\tilde{Q}) > 2n$, see Theorem 3). For the two-source extractors used in [11] (which require a stronger notion of independence which is not satisfied under our more general assumptions), it is known that randomness extraction is possible as long as $H_{\min}^{\varepsilon_s}(\mathbf{AB}|E_n)$ and $H_{\min}(\mathbf{Z}|\tilde{Q})$ have poly-logarithmic entropy [41]. Because for any $\mu < 50\%$ and $S_\mu > 0$, both $H_{\min}^{\varepsilon_s}(\mathbf{AB}|E_n)$ and $H_{\min}(\mathbf{Z}|\tilde{Q})$ contain a linear amount of entropy such extractors, if available, would allow for any bias μ for which $S_\mu > 0$ is achievable. This tradeoff is shown in Fig. S10. We remark that here it is not possible to give explicit finite size plots since the exact performance of such an extractor (if it even exists) is unknown. In any case, as can be seen in Fig. S10, the scaling between the required CHSH violation and the amount of tolerated bias μ is very unfavorable for $\mu \approx 50\%$.

IX. ADDITIONAL DATASET

Here, we discuss an additional dataset from an earlier run of the experiment in which one of the two QRNGs was not fully calibrated. The dataset consists of $n = 18 \cdot 2^{26} =$

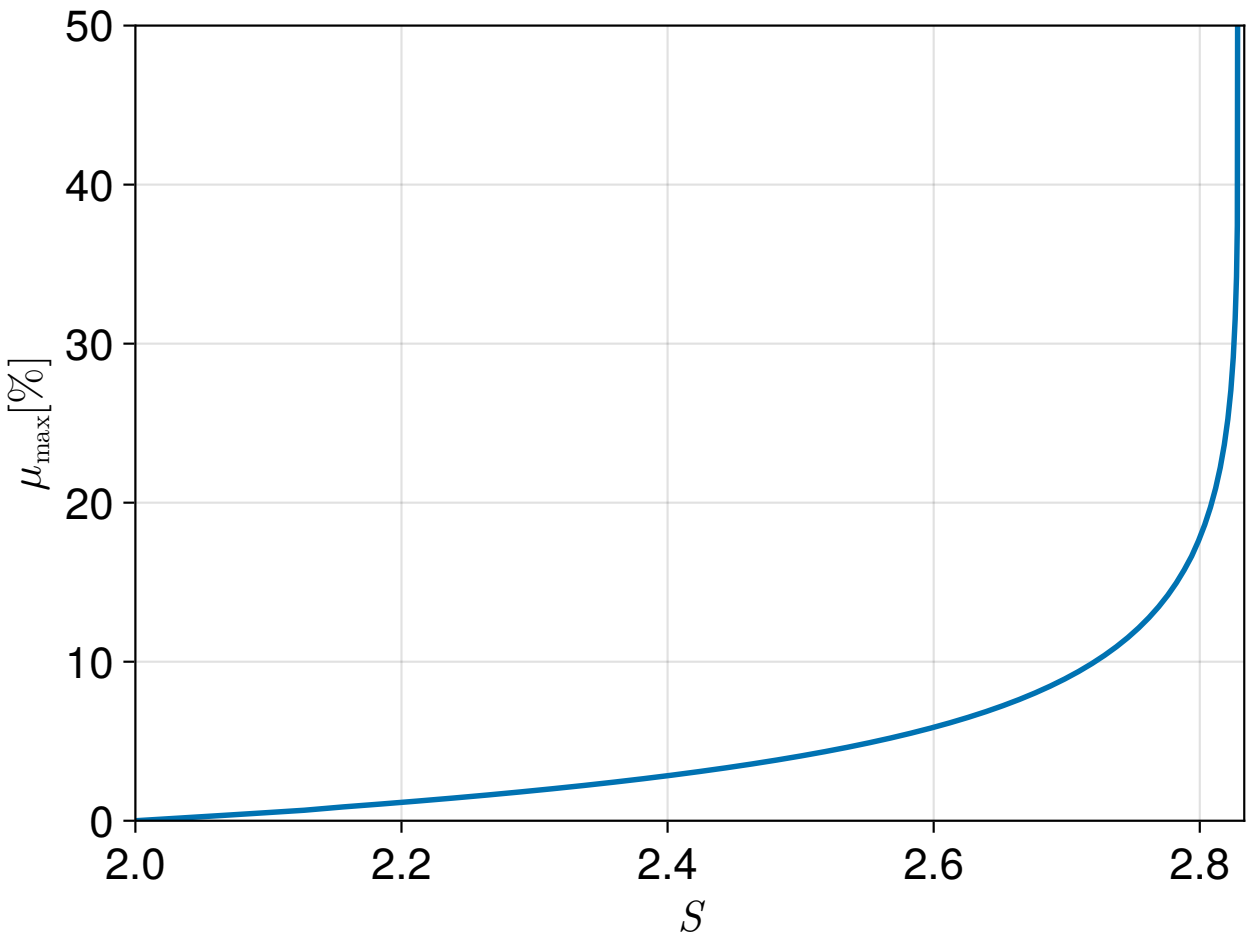

FIG. S10. The maximum bias $\mu_{\max}$ for which one can obtain $S_\mu > 0$ as a function of the CHSH value S. Assuming the existence of two-process extractors requiring sublinear entropies, $\mu_{\max}$ also corresponds to the maximum bias which can be tolerated (assuming sufficiently many trials n).

$1,207,959,552$ trials but due to a lack of calibration of Alice's QRNG, the inputs $X_1 \cdots X_n$ exhibit a statistical bias of roughly 0.2%. Assuming a, somewhat conservative, bias μ of $\mu = 0.3\%$, we obtain an average μ-MDL value of $S_{\mu,\mathrm{obs}} = 0.001187$. By Theorem 3, our device-independent randomness amplification protocol is able to extract uniform randomness despite the non-uniform input (even though $S_{\mu,\mathrm{obs}}$ is smaller than the value reported in the main text by more than a factor of two and would not allow for randomness amplification with $\mu = 0.75\%$). In total, we can extract $m = 13,701,145$ bits of uniform randomness. The extracted bitstring $\mathbf{K}$ exhibits a statistical bias of roughly 0.009%. This is within the expected statistical fluctuations for a bitstring of this length.

It is worth reemphasizing that this reduction in the statistical bias is *not* a proof that randomness amplification was successful. The goal of device-independent randomness amplification is to decouple the bitstring $\mathbf{K}$ from any information the adversary may have, which is impossible to verify experimentally. Rather, the security is derived from the observed value of $S_{\mu,\mathrm{obs}}$ through Theorem 4. Similarly, observing a statistical bias of 0.2% in the input only shows that $\mu \geq 0.2\%$ but does not provide an upper bound on μ since μ quantifies the probability of an adversary correctly guessing the inputs given the side information available to her. Conversely, the lack of statistical bias in the inputs $X_1 \cdots X_n$ and $Y_1 \cdots Y_n$ of the dataset in the main text does not show that $\mu = 0$.

* akulikov@phys.ethz.ch

[1] J. D. Schär, S. Storz, P. Magnard, P. Kurpiers, J. Lütolf, M. Gehrig, J.-C. Besse, A. Kulikov, and A. Wallraff, in preparation (2025).

[2] T. Walter, P. Kurpiers, S. Gasparinetti, P. Magnard, A. Potočnik, Y. Salathé, M. Pechal, M. Mondal, M. Oppliger, C. Eichler, and A. Wallraff, Phys. Rev. Appl. **7**, 054020 (2017).

[3] P. Kurpiers, T. Walter, P. Magnard, Y. Salathe, and A. Wallraff, EPJ Quantum Technology **4**, 8 (2017).

[4] S. Storz, J. Schär, A. Kulikov, P. Magnard, P. Kurpiers, J. Lütolf, T. Walter, A. Copetudo, K. Reuer, A. Akin, J.-C. Besse, M. Gabureac, G. J. Norris, A. Rosario, F. Martin, J. Martinez, W. Amaya, M. W. Mitchell, C. Abellan, J.-D. Bancal, N. Sangouard, B. Royer, A. Blais, and A. Wallraff, Nature **617**, 265 (2023).

[5] M. Santha and U. V. Vazirani, Journal of Computer and System Sciences **33**, 75 (1986).

[6] C. Abellán, W. Amaya, D. Mitrani, V. Pruneri, and M. W. Mitchell, Phys. Rev. Lett. **115**, 250403 (2015).

[7] B. Hensen, H. Bernien, A. E. Dreau, A. Reiserer, N. Kalb, M. S. Blok, J. Ruitenberg, R. F. L. Vermeulen, R. N. Schouten, C. Abellan, W. Amaya, V. Pruneri, M. W. Mitchell, M. Markham, D. J. Twitchen, D. Elkouss, S. Wehner, T. H. Taminiau, and R. Hanson, Nature **526**, 682 (2015).

[8] M. Giustina, M. A. M. Versteegh, S. Wengerowsky, J. Handsteiner, A. Hochrainer, K. Phelan, F. Steinlechner, J. Kofler, J.-A. Larsson, C. Abellán, W. Amaya, V. Pruneri, M. W. Mitchell, J. Beyer, T. Gerrits, A. E. Lita, L. K. Shalm, S. W. Nam, T. Scheidl, R. Ursin, B. Wittmann, and A. Zeilinger, Phys. Rev. Lett. **115**, 250401 (2015).

[9] L. K. Shalm, E. Meyer-Scott, B. G. Christensen, P. Bierhorst, M. A. Wayne, M. J. Stevens, T. Gerrits, S. Glancy, D. R. Hamel, M. S. Allman, K. J. Coakley, S. D. Dyer, C. Hodge, A. E. Lita, V. B. Verma, C. Lambrocco, E. Tortorici, A. L. Migdall, Y. Zhang, D. R. Kumor, W. H. Farr, F. Marsili, M. D. Shaw, J. A. Stern, C. Abellán, W. Amaya, V. Pruneri, T. Jennewein, M. W. Mitchell, P. G. Kwiat, J. C. Bienfang, R. P. Mirin, E. Knill, and S. W. Nam, Phys. Rev. Lett. **115**, 250402 (2015).

[10] M.-H. Li, C. Wu, Y. Zhang, W.-Z. Liu, B. Bai, Y. Liu, W. Zhang, Q. Zhao, H. Li, Z. Wang, L. You, W. J. Munro, J. Yin, J. Zhang, C.-Z. Peng, X. Ma, Q. Zhang, J. Fan, and J.-W. Pan, Phys. Rev. Lett. **121**, 080404 (2018).

[11] M. Kessler and R. Arnon-Friedman, IEEE Journal on Selected Areas in Information Theory **1**, 568 (2020).

[12] J. Kelsey, L. T. Brandão, R. Peralta, and H. Booth, *A reference for randomness beacons: Format and protocol version 2*, Tech. Rep. (National Institute of Standards and Technology, 2019).

[13] J. Bouda, M. Pawłowski, M. Pivoluska, and M. Plesch, Phys. Rev. A **90**, 032313 (2014).

[14] K.-M. Chung, Y. Shi, and X. Wu, "Physical randomness extractors: Generating random numbers with minimal assumptions," (2015), arXiv:1402.4797 [quant-ph].

[15] R. Ramanathan, "Finite device-independent extraction of a block min-entropy source against quantum adversaries," (2023), arXiv:2304.09643 [quant-ph].

[16] C. Foreman, S. Wright, A. Edgington, M. Berta, and F. J. Curchod, Quantum **7**, 969 (2023).

[17] R. Colbeck and R. Renner, Nat. Phys. **8**, 450 (2012).

[18] R. Gallego, L. Masanes, G. De La Torre, C. Dhara, L. Aolita, and A. Acín, Nature Communications **4**, 2654 (2013).

[19] F. G. S. L. Brandão, R. Ramanathan, A. Grudka, K. Horodecki, M. Horodecki, P. Horodecki, T. Szarek, and H. Wojewódka, Nature Communications **7**, 11345 (2016).

[20] R. Colbeck, *Quantum And Relativistic Protocols For Secure Multi-Party Computation*, Ph.D. thesis, University of Cambridge (2009), arXiv:0911.3814 [quant-ph].

[21] R. Colbeck and A. Kent, Journal of Physics A: Mathematical

and Theoretical **44**, 095305 (2011).
[22] S. Pironio, A. Acín, S. Massar, A. B. de la Giroday, D. N. Matsukevich, P. Maunz, S. Olmschenk, D. Hayes, L. Luo, T. A. Manning, and C. Monroe, Nature **464**, 1021 (2010).
[23] U. Vazirani and T. Vidick, in *Proceedings of the Forty-Fourth Annual ACM Symposium on Theory of Computing*, STOC '12 (Association for Computing Machinery, New York, NY, USA, 2012) p. 61–76.
[24] Z. Brakerski, P. Christiano, U. Mahadev, U. Vazirani, and T. Vidick, in *2018 IEEE 59th Annual Symposium on Foundations of Computer Science (FOCS)* (2018) pp. 320–331.
[25] S. Aaronson and S.-H. Hung, in *Proceedings of the 55th Annual ACM Symposium on Theory of Computing*, STOC 2023 (Association for Computing Machinery, New York, NY, USA, 2023) p. 933–944.
[26] M. Liu, R. Shaydulin, P. Niroula, M. DeCross, S.-H. Hung, W. Y. Kon, E. Cervero-Martín, K. Chakraborty, O. Amer, S. Aaronson, A. Acharya, Y. Alexeev, K. J. Berg, S. Chakrabarti, F. J. Curchod, J. M. Dreiling, N. Erickson, C. Foltz, M. Foss-Feig, D. Hayes, T. S. Humble, N. Kumar, J. Larson, D. Lykov, M. Mills, S. A. Moses, B. Neyenhuis, S. Eloul, P. Siegfried, J. Walker, C. Lim, and M. Pistoia, Nature **640**, 343–348 (2025).
[27] M. Tomamichel, *Quantum Information Processing with Finite Resources* (Springer International Publishing, 2016).
[28] R. Konig, R. Renner, and C. Schaffner, IEEE Transactions on Information Theory **55**, 4337 (2009).
[29] G. Gour and M. M. Wilde, Phys. Rev. Res. **3**, 023096 (2021).
[30] G. Pütz, D. Rosset, T. J. Barnea, Y.-C. Liang, and N. Gisin, Phys. Rev. Lett. **113**, 190402 (2014).
[31] M. Sandfuchs, C. Ferradini, and R. Renner, "Randomness from causally independent processes," (2025), arXiv:2510.05203 [quant-ph].
[32] Y. Dodis, A. Elbaz, R. Oliveira, and R. Raz, in *Approximation, Randomization, and Combinatorial Optimization. Algorithms and Techniques*, edited by K. Jansen, S. Khanna, J. D. P. Rolim, and D. Ron (Springer Berlin Heidelberg, Berlin, Heidelberg, 2004) pp. 334–344.
[33] C. Foreman, R. Yeung, A. Edgington, and F. J. Curchod, Quantum **9**, 1584 (2025).
[34] F. Dupuis, O. Fawzi, and R. Renner, Communications in Mathematical Physics **379**, 867 (2020).
[35] E. Knill, Y. Zhang, and H. Fu, "Quantum probability estimation for randomness with quantum side information," (2023), arXiv:1806.04553 [quant-ph].
[36] Y. Zhang, L. K. Shalm, J. C. Bienfang, M. J. Stevens, M. D. Mazurek, S. W. Nam, C. Abellán, W. Amaya, M. W. Mitchell, H. Fu, C. A. Miller, A. Mink, and E. Knill, Phys. Rev. Lett. **124**, 010505 (2020).
[37] T. Metger, O. Fawzi, D. Sutter, and R. Renner, in *2022 IEEE 63rd Annual Symposium on Foundations of Computer Science (FOCS)* (2022) pp. 844–850.
[38] P. H. Eberhard, Phys. Rev. A **47**, R747 (1993).
[39] J. F. Clauser, M. A. Horne, A. Shimony, and R. A. Holt, Phys. Rev. Lett. **23**, 880 (1969).
[40] A. Acín, N. Brunner, N. Gisin, S. Massar, S. Pironio, and V. Scarani, Phys. Rev. Lett. **98**, 230501 (2007).
[41] R. Arnon-Friedman, C. Portmann, and V. B. Scholz, in *11th Conference on the Theory of Quantum Computation, Communication and Cryptography (TQC 2016)*, Leibniz International Proceedings in Informatics (LIPIcs), Vol. 61, edited by A. Broadbent (Schloss Dagstuhl – Leibniz-Zentrum für Informatik, Dagstuhl, Germany, 2016) pp. 2:1–2:34.
[42] C. Foreman, L. Wooltorton, K. Milner, and F. J. Curchod, "An efficient construction of raz's two-source randomness extractor with improved parameters," Cryptology ePrint Archive, Paper 2025/1156 (2025).
[43] P. Brown, H. Fawzi, and O. Fawzi, Quantum **8**, 1445 (2024).
