# Supplementary material for: Experimental randomness amplification
Source: Nature. 2026 May 27;653(8116):1033–8. doi: 10.1038/s41586-026-10521-8 (PMC13658030; doi:10.1038/s41586-026-10521-8)
Supplement: Supplementary file 2 [file 41586_2026_10521_MOESM2_ESM.pdf]

nature portfolio

Peer Review File

Experimental Randomness Amplification

Corresponding Author: Dr Anatoly Kulikov

Any redactions in this file are there to maintain patient confidentiality, the confidentiality of unpublished data, or to remove third-party material.

This file contains all reviewer reports in order by version, followed by all author rebuttals in order by version.

Attachments originally included by the reviewers as part of their assessment can be found at the end of this file.

Version 0:

Reviewer comments:

Referee #1

(Remarks to the Author)
The manuscript entitled “Experimental Randomness Amplification” by Kulikov et al. presents an experimental demonstration of a randomness amplification protocol proposed in [M. Kessler and R. Arnon-Friedman, IEEE Journal on Selected Areas in Information Theory 1, 568 (2020)] (Ref. 20). Key contributions include 1) the establishment of theoretical guarantee for generating perfect randomness from imperfect random sources that are within an experimentally accessible regime and 2) the experimental realization of perfect randomness using loophole-free Bell test with improved stability, repetition rate, and data quality compared to previous work [S. Storz et al., Nature 617, 265 (2023)] (Ref. 29). The experimental demonstration of the utility of quantum nonlocality is of great interest to the quantum information community. However, I feel the manuscript requires substantial further work before being ready for publication.

My primary concern is the absence of direct experimental evidence supporting the manuscript’s main conclusion. The randomness amplification procedure demonstrated in this work requires the coordinated operation of two Santha–Vazirani (SV) sources and a two-node quantum device, followed by classical post-processing using a randomness extractor. However, the authors only provide the experimental measurements of the \mu-MDL inequality, which verifies the soundness of the data generated by the quantum device. This means that, to conclude that randomness amplification was successful, one must assume the correctness of all other experimental steps and theoretical derivations. From an experimental standpoint, this work demonstrates a violation of the \mu-MDL inequality rather than directly proving randomness amplification. Since the experiment consumes imperfect random bitstrings and outputs a perfect random bitstring, I believe the direct comparison of randomness between the input and output bitstrings is necessary to confirm that randomness amplification has indeed occurred. For example, the author claim that the SV source is tested to support an unpredictability bias \mu below 0.75%. What would be the corresponding value if similar tests were applied to the output bitstring? Additionally, the authors mention in the introduction that “correlations have been found in experimental tests of commercially available random number generators”. Are these correlations less significant in the perfect random number generator produced in this work? Including such analyses would substantially strengthen the credibility of the conclusions.

I find the manuscript somewhat lacking in quantitative detail. The authors claim that the output randomness is perfect for any adversary whose predictability \mu of the input randomness is below 0.75%, yet they do not explain how this specific threshold is determined. Relatedly, while Fig. 2 illustrates the relationship between the tolerated bias \mu, the CHSH value, and the number of trials required, the methods used to derive this relationship and perform the corresponding numerical calculations are not provided. In particular, the influence of the trial number on the results warrants a more explicit explanation. In Fig. 4, error bars should be included. It is also unclear what value of \mu was used when calculating the \mu-MDL values presented. In Results and Discussion section, the length of the input and final random bitstring is given. The authors should explicitly explain how this randomness extraction procedure is achieved. What are the values of n and d introduced in Box I? And why such a particular choice?

Minor points:

- I am not sure if it is appropriate to classify this experiment as demonstrating "quantum advantage." In some respects, the

situation of quantum randomness amplification is similar to quantum error correction - a task that also cannot be achieved through classical information processing. Yet, we do not typically label quantum error correction experiments as examples of quantum advantage. Therefore, I would recommend using the term "quantum utility" instead.
- What is the efficiency of the active all-microwave reset used in this work? Does the reset error impact the results, considering it could potentially introduce correlations in the output bitstream?
- Fig. 3 caption: The abbreviation "QRNGs" is used here without being defined upon its first mention.
- Page 3, line 58: The abbreviation "MDL" is used here without being defined upon its first mention.
- The symbol S_{\mu, obs} is frequently used in the manuscript, but its definition is provided quite late in the Methods section. To improve readability, I would recommend introducing its definition earlier in the main text.
- Page 6, line 50: “an observation of S_{\mu, obs}<0 certifies \varepsilon-perfect randomness”, please check.
- The symbol \varepsilon is first introduced in the main text as the failure probability, while in the Methods section it appears to denote a metric quantifying the closeness between two distributions. Are these two usages referring to the same concept?
- Eq. 1: What is the meaning of P_{X\Gamma_X}?
- Page 6, line 48: What is the definition of min-entropy?
- Page 7, line 35: Further explanation on the conditional min-entropy is required. Is this the same as the min-entropy mentioned previously? What is the physical meaning of this quantity? What is the meaning of the symbol \varepsilon here?
- Fig. 5: “extraxctor”.

Referee #2

(Remarks to the Author)
In this work, the authors present the first experimental demonstration of device-independent randomness amplification, building on a loophole-free Bell test platform developed by the same group a coupe of years ago. Operating at a trial rate of 50 kHz and achieving a CHSH Bell violation of 2.271 per trial, the protocol can tolerate a bias of up to 7.5e-3 in every input random bit. Over about 9 hours of data acquisition, the authors collected n=20*2^{26} Bell-test trials, from which they extracted 20,431,465 high-quality random bits using 5,368,709,120 (four times of the number of Bell-test trials) biased random bits. This marks a notable amplification of input randomness.

This achievement was made possible through several experimental improvements over their prior work [Nature 617, 265 (2023)] made this possible: (1) reduced photon loss between nodes, enhancing entanglement fidelity and Bell violation; (2) increased repetition rate; (3) shortened readout and data processing time; and (4) improved system stability, evidenced by the absence of timing jitters over multiple days of continuous operation. These technical advances, combined with recent theoretical development, enable the landmark demonstration reported in this work.

The manuscript clearly explains most aspects of the work. However, there are statements that I found unclear or in need of improvements. My comments and questions are as follows:

1) About title: Would it be more informative to include the term "device-independent" in the title? Are there also semi-device-independent protocols for randomness amplification, and are all such protocols necessarily based on Bell tests?

2) Fig. 2 in the main text shows that with a high CHSH Bell violation and a large number of trials, a bias larger than 4% can be tolerated. In the meantime, I recall that Ref. [55] (Phys. Rev. Lett. 113, 190402) showed that even very large biases (up to 0.5 in each bit) can be tolerated for a loophole-free Bell test. Can the authors comment on the source of this apparent mismatch? Besides improvement on extractors, is there anything else required? Also, I noticed that several works mentioned in the plot of the figure are not included in the reference list.

3) Line 31 on Page 4: The abbreviation "FPGA" appears for the first time in the main text. Please define it on first use.

4) Line 50 on Page 4: The sentence "Both quantities are stable ...." may be misleading, as Fig. 4 shows noticeable drifts of both quantities.

5) Regarding the first sentence in the caption of Fig. 4 "each point corresponds to ....": The plot in the figure doesn't show these points but only smooth curves, right or not?

6) Line 32 on Page 5: I would like to point out the recent work [Nature 642, 916–921 (2025)] for a public device-independent randomness beacon.

7) To achieve randomness amplification, the author employed the MDL inequality in Eq. (4) on Page 7. However, from the current presentation it is unclear whether the measurement operators were chosen
to maximize the violation of the MDL inequality given a specific adversarial bias or to directly maximize tolerable adversarial bias. It would be helpful to include a section in the Supplementary Information detailing the prepared quantum state, the measurement operators used, and the criteria or method for choosing these operators.

As a side note, I would like to point out that using the MDL inequality is not the only way for addressing adversarial biases in measurement setting choices. Building on the convex-polytope structure of the set of MDL correlations characterized in Phys. Rev. Lett. 113, 190402 (Ref [55] of this work), alternative methods are available. For example, Section 3.4 of arXiv:1806.04553 provides a general treatment, and Phys. Rev. Lett. 124, 010505 presents a specific implementation.

8) Line 35 on Page 7: Please explain the smoothness parameter \epsilon.

9) Lines 38-39 on Page 7, the sentence "This means that our protocol remains secure even if the adversary learns the outputs of the SV source.": I believe the security requires that the adversary learns the outputs of the SV sources only after the protocol implementation. If the authors agree, please clarify this.

10) Please specify the base of all logarithmic expressions used in both the main text and Supplementary Information.

11) Line 73 on Page 9: Please define AWG at first occurrence.

12) Lines 38-41 on Page 12, the sentence "we install an oscilloscope at each node (part of the trusted setup), to detect the arrival time of specific reference pulses output by electronic devices in the setup (part of \lambda)": Does the inclusion of reference pulses in \lambda imply that they are not trusted?

13) The subtitle of the left panel in Figure 8 may need a bit of clarification.

14) Line 29 and Table II on Page 13: Are there any uncertainties associated with the measured values? Are these measured values constant across trials?

15) Assumption 5 in Section V: Please elaborate on the motivation or justification for this assumption.

16) Theorem 1 in Section VI needs clarifications. The description of “step 1” is ambiguous. Please define the protocol clearly and introduce error parameters before using them. Also, please provide an explicit expression for the function \eta.

17) Definition 2 in Section VI: Please clarify what is the Markov model. Under which assumption is this model considered sufficient for security?

18) Theorem 4 in Section VI: Are the error parameters \epsilon_1, \epsilon_2, and \epsilon independent? How to choose these error parameters and the smoothness parameters in order to prove Theorem 5?

19) Theorem 5 in Section VI: I suggest the authors provide a proof sketch or more details. In addition, can the authors provide the values of each parameter used in their implementation of the randomness amplification protocol?

20) Remark after Theorem 5: Please clarify that the adversary can only learn the output of the source (XYZ) after their use in the protocol.

21) If the security proof is according to Theorem 6 in Section VI, please specify the values of all relevant parameters used in the protocol implementation. Also, please provide an expression for the parameter m_i in the proof of the theorem.

22) A comparison between Eq. (19) and Eq. (22) on Page 16 suggests that Theorems 4 and 5 employ different security definitions: Theorem 4 appears to rely on the trace distance conditioned on success, whereas Theorem 5 incorporates the product of the conditional trace distance and the associated success probability. Please clarify which security definition is used for the final result reported in this work.

23) In Section VII, please provide details regarding the implementation of the chosen two-source extractor. For example, what software was used and what type of classical hardware was employed. Also, please report the time required to complete the extraction.

Overall, I am impressed by the quality of the results and would be happy to recommend acceptance, provided that the points above are addressed or clarified.

Referee #3

(Remarks to the Author)
Summary, conclusions and significance: In the paper titled “Experimental Randomness Amplification”, the authors report on the experimental implementation of a Device-Independent Randomness Amplification protocol. Specifically, they consider randomness amplification of weakly random sources (assumed to possess a Santha-Vazirani structure with a bias of at most 0.75%) and report a loophole-free Bell test that allows to amplify this source to produce a string of fully random bits. Overall, the experimental produced a total of over 20 million random bits (random up to a small bias of 10^{-12}) from over 5 billion weakly random seed bits, using over 10^9 trial runs performed in a time of about 9 hours. The experimental implementation has impressive features over previous ones - a substantially high Bell violation (a very impressive CHSH value of about 2.271 in a fully loophole-free setup), high repetition rate to shorten the data acquisition time, and high multi-day stability of

room-temperature control electronics and calibration. In particular, fully loophole-free implementations achieving Mbit-range output bit strings have not been done before. The experiment is thus very significant and given the importance of the task of randomness amplification, will be of wide interest to a broad community of researchers in quantum information and cryptography.

Data & methodology, clarity and context: The methodology is appropriate, and all necessary data is stated to be available from the corresponding authors upon request. The paper is very well-written, the methodology and conclusions are stated clearly and lucidly.

Originality: On the other hand, it must be noted that the paper does not contain any theoretical novelty - the randomness amplification protocol and the assumptions (Section V in the Supplemental Information) are from Ref. [20] Kessler and Arnon-Friedman “Device-independent Randomness Amplification and Privatization” IEEE Journal on Selected Areas in Information Theory 1, 568 (2020), and the security proof against quantum adversaries (Section VI in the SI) is the one given in Ref. [20] using the extractors in Refs. [53, 58]. The manuscript as such mainly reports an improved loophole-free Bell test from some of the previous works such as Refs. [29, 39] for the task of DI randomness amplification. As such, the manuscript in my opinion does not pass the high bar of suitability for publication in the journal Nature.

The authors might wish to consider the following comments and suggestions for future.

1) Essentially, the main feature of the manuscript is the achievement of a high generation rate in randomness amplification in a loophole-free Bell experiment. It is unclear whether using the data from other loophole-free Bell tests not cited in the manuscript - such as “Loophole-free test of local realism via Hardy's violation” Phys. Rev. Lett. 133, 060201 (2024) from the Pan group - cannot be used to achieve the Mbit-range randomness amplification reported here, especially for such a low value of the bias (\mu \leq 0.0075) as considered here.

Secondly, as a minor point, it is unclear how crucial a fully loophole-free implementation is for the task of DI quantum randomness generation. It has been argued in several places in the literature (see for example Liu et al. “Device-independent randomness expansion against quantum side information” Nature Physics 17, 448–451 (2021) and Pirandola et al. “Advances in Quantum Cryptography” Adv. Opt. Photon. 12, 1012-1236 (2020)) that in DI-QRNG experiments (as opposed to DI-QKD experiments), it may not be strictly necessary to close the locality loophole. DI-QRNG experiments are instead considered as performed by a single user Alice who has a secure laboratory and can prevent any devices from receiving or sending communication at will (given that the devices are assumed to be shielded and not leaking any output bits to the adversary, failing which any DI protocol is rendered insecure). The important thing is to ensure that, before giving its output, each device only learns its own input and not that of the other device which can be ensured by shielding the devices. This is sufficient because the aim of the present experiment is not to disprove local realism. In this context, other experiments that do not close the locality loophole such as those cited in Fig. 2 as well as others such as “Practical No-Signalling proof Randomness Amplification using Hardy paradoxes and its experimental implementation” in arXiv: 1810.11648 should also be taken note of.

2) It would be good to clarify already in the Title, Abstract and Introduction that the experimental implementation is of a Device-Independent protocol, since there are semi-device-independent randomness amplification protocols in the literature (see for example “Semi-device-independent full randomness amplification based on energy bounds” by Senno and Acin in arXiv:2108.09100).

It would also be good to clarify in the section on “Quantifying Randomness” that it is assumed that the eavesdropper’s side information ‘e’ about the bits from the SV-source is purely classical, i.e., no quantum side information about the source is presumed here.

3) The reference list on DI randomness amplification is far from complete, especially a number of papers showing security against no-signalling adversaries as well as papers on amplifying min-entropy sources are not cited.

It would also be interesting to check whether the generation rates can be increased with improved quantum-proof extractor constructions such as the one by Foreman et al. in “An efficient construction of Raz's two-source randomness extractor with improved parameters” arXiv: 2506.15547.

4) Minor typos: in the Methods section in page 6, shouldn’t it be S_{\mu, obs} > 0 allows to prove the independence condition and certifies randomness? In page 3, it is stated that the source has been extensively tested, and that the results are compatible with a bias below 0.75% - but given that the bias quantifies the predictive power of an adversary, it cannot be estimated by any statistical test, and as such the claim that it is below 0.75% may be confusing to the reader.

Referee #4

(Remarks to the Author)
Summary of the key results

This paper presents an experiment for randomness amplification, the task of transforming imperfect random sources into truly random outputs. The task is essential to many applications using randomness. For example, in modern cryptography, it

is known that if the security keys are generated using imperfect random bits, the public information becomes sufficient to recover the secrets.

Verifying whether a source produces unpredictable bits can not be done by observing (the statistics of) the samples since it is an inherent property of the device. More concretely, it is known that one cannot certify randomness generated by a purely classical device. In contrast one can present a protocol that certifies randomness via a test that accepts a quantum device and meanwhile rules out classical ones. Therefore known protocols for this task are tied to demonstrations of quantum advantage.

Previously there has been multiple experiments (e.g., Ref. [29]) that demonstrate violations of Bell's inequality, tests that demonstrates quantum entanglement sharing between two devices out of communication. These experiments, while closing all other loopholes, require an assumption that the inputs to these devices must be uniformly random bits. This work builds upon the experiment in Ref. [29] and the theory in Ref. [20], Ref. [53], and Ref. [58] to further remove this assumption. In particular, this paper shows that if the inputs are weakly correlated in the sense that they are sampled from Santha-Vazirani sources, one can still produce certifiable random bits experimentally.

Originality and significance

The theory part appears to be fully drawn from pervious results (Ref. [20], Ref. [53], and Ref. [58]), and the main contribution in this paper is the upgrade of the experiment in Ref. [29] (which only demonstrates a violation of Bell's inequality) for the first experiment for randomness amplification. As far as I can tell, the security proofs (Supplementary Material, Theorems 5-6) are only straightforward combinations of previous results, and the theory is no particularly tailored for the experiment.

As a theorist, I cannot tell how difficult these experimental improvements are to achieve, but I will summarize them anyway. First, this new experiment not only demonstrates a violation of Bell's inequality (as in Ref. [29]) but also demonstrates certifiable randomness amplification. The number of trials (rounds) are significantly increased from a million to 1.34 billion trials; therefore the completion of a single round is more efficient. The experiment also adds Santha-Vazirani sources as inputs to the devices, with the level of predictability $\mu \leq 0.75\%$. It also increase the S value from 2.07 to 2.271. A higher violations tightens the bound on generated entropy (the entropy per round is a monotonic function in the violation). This protocol uses a two-source extractor in Refs. [61-62] for efficiently concerns. The channel loss is decreased from 19% to about 12-14%. This experiment claims to produce ~20 million random bits in nine hours. Compared to the very recent experimental work using random circuit sampling [27], the generation and verification appears to be very efficient.

Data and methodology

I did not spot any error on the presentation at this moment. Since the approach are based on previously known theory, there is no obvious reason to believe there is a loophole on the theory part. The quality of the presentation is quite high, and it is very accessible—I can understand most of the details in a reasonable timeframe, without reading through previous results.

Compared to Ref. [29], this new experiment adds a Santha-Vazirani (SV) source for the generation of inputs. Since this SV source are implemented using QRNG, I am not sure if this opens a loophole by assuming that the QRNG is SV since the experiment to test the QRNG seems to only heuristically conclude it is SV. I was expecting that the use of quantum sources and their structure (e.g., two devices are out of communication) should be all certified experimentally for a results that claims to be loophole-free. Furthermore, it is said that the bias of the SV source satisfies $\mu \leq 0.75\%$, conditioned on the adversary's information. I am not very convinced of how this is obtained as explained. If it is from some empirical estimation I am not sure whether one can conclude the source is SV.

Suggested improvements

As explained in the Data & Methodology section, I believe that the authors can further explain how they can experimentally conclude that the inputs are SV sources.

References

The paper offers appropriate credit to previous work.

Clarity and context

The presentation is very clear, and the abstract, introduction, and conclusions well explains the contexts and the results.

Version 1:

Reviewer comments:

Referee #1

(Remarks to the Author)
The manuscript has been significantly improved through the authors' revisions, which provide additional support for the theoretical framework and further clarification of the experimental details. The presentation is also clearer. I am satisfied with

authors' response to my concerns regarding the soundness of the randomness amplification results. Given that the work demonstrates the generation of randomness beyond the reach of any classical device, it can be described as achieving quantum advantage. Therefore, I am happy to recommend publication.

Referee #2

(Remarks to the Author)
I would like to thank the authors for their detailed response and the revisions made to in the manuscript. The authors have addressed most of the points raised in my first report, and the manuscript becomes more accessible to general readers. Furthermore, the authors have upgraded their security analysis. By allowing for quantum side information about the random sources used, they have improved over prior security analyses of device-independent randomness amplification. This significantly enhances the impact of the work. I am therefore pleased to recommend this impressive work for publication in Nature.

At the same time, I list a few minor points below that I hope the authors can clarify before final acceptance:

1) The abbreviation "QRNG" is not defined before its first use in the paper.

2) Regarding the sentences "... However, in contrast to quantum random number generation, randomness amplification guarantees that the output is perfect provided that a minimal set of assumptions hold (see Supplementary Information V for a detailed list). In the language of Bell tests, this requires the implementation to be loophole-free (see Methods for a discussion of possible experimental loopholes)." on Page 2: This phrasing is a bit misleading. There are different types of quantum random number generators. Some of them are also based on loophole-free Bell tests and require only a minimal set of assumptions, although the minimum set required differs from the set used in this work.

3) Regarding the response to my previous comment 7): As far as I can tell, the measurement operators are only implicitly mentioned in the sentence "At each node, as in Refs. [35, 36], the random input bit controls the state of a microwave switch which conditionally passes a microwave pulse to rotate the qubit by π/2 around a controlled axis, implementing the measurement basis selection." on Page 5. Consequently, the specific operators performed are still not fully clear. I suggest the authors explicitly list these measurement operators in the main text.

4) Regarding Definition 2 on Page 17: The notation in Eq. (16) is unclear. Could the authors clarify what states are represented on the left-hand sides of these inequalities?

5) Regarding Definition 3 on Page 18, I have two questions: I) Are there typos in Eq. (19)? My understanding is that the instrument M transforms system A into systems X and S. However, the conditioner in the min-entropy term is listed as systems S and B. Should this not be systems S and A? A similar problem exists for instrument N and its associated min-entropy. II) This definition appears to consider only pure initial states. Could the authors comment on its applicability in practical scenarios where the purity of the initial state is not guaranteed?

Referee #3

(Remarks to the Author)
In the revised version of the paper on Experimental Randomness Amplification, the authors have addressed the concerns raised in the previous referee report. The authors acknowledge that the individual component — high-fidelity entanglement generation, high-rate and high-quality qubit control, closure of the locality loophole — have each appeared in previous papers, and the key point of the present work is to combine and integrate these elements into a single robust experimental demonstration of SV source amplification. The revised paper is very nicely written and the claim is clear. The references have been improved and are now more comprehensive. The loophole-free implementation of device-independent SV-source amplification achieving Mbit-range output bit strings is of definite high interest to the community. Especially, the combination of high-fidelity entanglement generation and the substantially high Bell violation (CHSH value of 2.271) in a loophole-free setup, and high repetition rates are strengths of the paper.

1) A main practical weakness of the demonstration is that the initial source is already assumed to be quite close to uniform (each of the 5.4 billion bits taken from the SV source is assumed to be at most 0.0075 away from perfectly random) to obtain 45 million bits that are 10^{-12} away from uniform. This renders figures such as Fig. 13 and Fig. 14 somewhat of an oversell, since evidently even applying a one-time pad with the initial SV source bits would render the photo of the sheep equivalent to noise.

2) In relation to question 1, one is lead to question the set of assumptions claimed in Section V. One reason for the small value of the initial bias tolerated (which was also pointed out in the earlier refereeing round) stems from the closing of the locality loophole. While closing the locality loophole is experimentally demanding, it is commonly accepted in the community (such as in Liu et al. "Device-independent randomness expansion against quantum side information" Nature Physics 17, 448–451 (2021) and Pirandola et al. "Advances in Quantum Cryptography" Adv. Opt. Photon. 12, 1012-1236 (2020)) that DI-QRNG experiments can and should be run under the assumption of shielding. Clearly, closing of the locality loophole has meant that the value S_{\mu} is quite small, so that the maximum tolerated initial bias \mu (calculated according to Eq.(60)) is also extremely small. It therefore feels as if the authors have attempted to adapt earlier loophole-free Bell experiments (where closing of the locality loophole is strictly necessary to rule out local realism) to DI randomness generation experiments where such a closing of the locality loophole is overkill.

The authors have attempted to justify this by claiming that they have removed the shielding assumption in comparison to previous experiments. They state in the Section V on Assumptions that the shielding assumption “is required if one wants the random bit string K to remain private after it was produced. However, there are numerous applications where a source of public randomness is sufficient (for instance, lotteries or randomness beacons) and therefore we do not impose this assumption”. This looks very unclear. Isn’t the Shielding assumption still required in the current paper to assume that the devices are not leaking any output bits from the MDL test to the adversary, before these are processed along with other SV source bits to produce the final bit string K? Keeping Alice’s devices spacelike separated is not on its own sufficient to prevent them from leaking output bits to the adversary, unless one assumes that they are shielded from said adversary. Therefore, irrespective of whether one thinks of an application such as a lottery or a randomness beacon where the final bit string K of the protocol is revealed to all, it seems to me that a shielding assumption on the devices is necessary in the DI protocol. The authors might wish to clarify this in the section on the claimed applications to lotteries and randomness beacons.

3) A slightly technical question concerns the “Single-round entropy bound” used in Section VI. Security Proof. The authors use the single-round von Neumann entropy bound from Ref. [66] derived for the CHSH inequality. They then bound in Eq. (54) H(A_i B_i |E_i \tilde{E}_{i-1}) by H(A_i | E_i \tilde{E}_{i-1}). This is a quite bad bound given that the joint outputs (A_i, B_i) can in principle contain up to twice as much entropy as the outputs A_i alone, and seems to be an artefact of the analysis used in Ref. [20] (where the objective was not a practical experiment but a proof-of-principle protocol). Some recent works (see for example arXiv: 2411.04858 or Quantum 7, 1114 or Quantum 8, 1445) have computed the single round min-entropy (and numerically the von Neumann entropy) for the joint outputs which should result in an increase in the overall number of output bits from 45 million by a potential factor of \alpha < 2.

4) Regarding a comment from the previous report about the lack of theoretical novelty in the work, the authors have now updated the paper to a claim that the updated security proof allows for quantum side information about the SV sources. This is a beautiful theoretical result which will definitely be appreciated in the community.

It would be great if the corresponding result could be included in the current paper to add weight to the theoretical contributions made here. Unfortunately, the result is only mentioned in passing with the section on “Two-process extractors” being quite brief. In particular, Definition 3 and Eq.(19) deserve more explanation regarding the assumptions on H_{\min}(X|SB) and H_{\min}(Y|TA), similar assumption should also be present in eq. (21)? The idea seems to be that processes in space like separated regions are described by product maps (which gives rise to an assumption of independent processes as opposed to independent sources), and one can extract uniform randomness from two such processes if they yield sufficient randomness to begin with.

Some motivation on considering quantum side information on the source may be included given that this is the central theoretical novelty (rather than a contribution since the details are missing here). Apart from a statement that the SV source is quantum so that the adversary can possess systems entangled with it, it is unclear why overcoming this assumption is of high importance for a practical experimental implementation.

Referee #4

(Remarks to the Author)
This version provides a new security analysis that improves over what was offered in the previous version. In particular, the authors consider the setting where the adversary may have access to quantum side information about the quantum Santha-Vazirani source. In this setting, a generalized entropy accumulation theorem (GEAT)--in particular, assuming that the adversary learns part of the output in each round--was used to reduce the entropy accumulation to proving a constant lower bound for a single-round von Neumann entropy. The single-round analysis looks not too different from that in [Kessler, Arnon-Friedman]. While the overall combination specifically for randomness amplification is new, the ingredient was offered in previous papers.

In comparison to the previous version, this one addresses my questions.

Other comments:

- Some notations are not properly defined or explained (and I have to guess what they really mean). For example, the state \rho_{Q\wedge xy} seems to mean the classical part is projected onto a particular outcome (i.e., onto a standard basis vector). There are more, and I would think it helps the reader to include all the relevant definitions, even when they look obvious to experts.
- There is no reference for GEAT. I think it's from [Metger, Fawzi, Sutter, Renner] but it would be great if the authors can clarify.
- The proofs of Lemma 1 and Lemma 2 use Remark 1, but there is no Remark 1, and the hyperlink goes to Definition 1.

Open Access This Peer Review File is licensed under a Creative Commons Attribution 4.0 International License, which permits use, sharing, adaptation, distribution and reproduction in any medium or format, as long as you give appropriate credit to the original author(s) and the source, provide a link to the Creative Commons license, and indicate if changes were made.
In cases where reviewers are anonymous, credit should be given to 'Anonymous Referee' and the source.
The images or other third party material in this Peer Review File are included in the article's Creative Commons license, unless indicated otherwise in a credit line to the material. If material is not included in the article's Creative Commons license and your intended use is not permitted by statutory regulation or exceeds the permitted use, you will need to obtain permission directly from the copyright holder.
To view a copy of this license, visit https://creativecommons.org/licenses/by/4.0/

Note from the authors: In the following document we show the referee's comments in black and display our responses in blue.

Referee #1 (Remarks to the Author):

The manuscript entitled "Experimental Randomness Amplification" by Kulikov et al. presents an experimental deamonstration of a randomness amplification protocol proposed in [M. Kessler and R. Arnon-Friedman, IEEE Journal on Selected Areas in Information Theory 1, 568 (2020)] (Ref. 20). Key contributions include 1) the establishment of theoretical guarantee for generating perfect randomness from imperfect random sources that are within an experimentally accessible regime and 2) the experimental realization of perfect randomness using loophole-free Bell test with improved stability, repetition rate, and data quality compared to previous work [S. Storz et al., Nature 617, 265 (2023)] (Ref. 29). The experimental demonstration of the utility of quantum nonlocality is of great interest to the quantum information community. However, I feel the manuscript requires substantial further work before being ready for publication.

My primary concern is the absence of direct experimental evidence supporting the manuscript's main conclusion. The randomness amplification procedure demonstrated in this work requires the coordinated operation of two Santha-Vazirani (SV) sources and a two-node quantum device, followed by classical post-processing using a randomness extractor. However, the authors only provide the experimental measurements of the \mu-MDL inequality, which verifies the soundness of the data generated by the quantum device. This means that, to conclude that randomness amplification was successful, one must assume the correctness of all other experimental steps and theoretical derivations. From an experimental standpoint, this work demonstrates a violation of the \mu-MDL inequality rather than directly proving randomness amplification.

We thank the referee for the careful reading of our manuscript and for the positive assessment of our experimental and theoretical work. We address below the main concern regarding the extent to which our results constitute a conclusive experimental demonstration of randomness amplification.

The referee is correct that, in our experiment, only the data produced by the untrusted quantum device are verified in a device-independent manner. To conclude that randomness amplification was successful, we must also ascertain the correctness of the theoretical framework linking the observed Bell-test violation to certified output randomness and assume the proper functioning of the trusted components of the setup. We would like to emphasize that the correctness of the theoretical framework is not an assumption: it is subject to proof and verification, which can be done by an external person or a user of the device/protocol. We provide such a proof in the Supplemental Material section VI. In the revised version, the proof is revised and extended to include quantum side-information.

The assumptions regarding the proper functioning of the trusted components of the setup are explicitly stated and discussed in the manuscript: the division into trusted and untrusted parts is shown in Fig. 6 of the revised manuscript (formerly Fig. 5), and the only experimental step which needs to be assumed correct – as per the interface of the protocol in Box I and its experimental implementation in Fig. 4 – is the randomness extraction. We assume that the input RNGs are SV sources. We provide a complete list of assumptions in Supplementary Information V. Apart from these explicitly defined trust elements, no further experimental steps are assumed.

To improve the clarity of the manuscript and address the reviewer's concerns, we have revised the *Quantifying Randomness* section of the main text (now titled *Amplifying Randomness*), introduced a new Fig. 3, and updated the *Randomness extraction* section of the Methods to make the logical connection between the observed data, the theoretical proof, and the claim of successful randomness amplification more transparent. We added the description of our input RNGs, and the justification for them being SV sources (based on the excess predictability specified by the manufacturer) to the end of Supplemental Material section I ('Experimental Devices').

Since the experiment consumes imperfect random bitstrings and outputs a perfect random bitstring, I believe the direct comparison of randomness between the input and output bitstrings is necessary to confirm that randomness amplification has indeed occurred.

A direct comparison of randomness between the input and output bitstrings as requested by the Referee is fundamentally impossible under the operational definition of randomness – as we would need to prove the absence of correlations between our random outputs and *everything* in its past light cone. This impossibility, in a broad sense, is precisely what makes the field of randomness generation difficult and fruitful – it is not possible to directly compare the randomness of strings, and hence we need to rely on theoretical proofs. This limitation is intrinsic to the problem and motivates the very existence of device-independent protocols. Within this framework, our experiment provides the strongest possible certification: the observed μ-MDL inequality violation, together with the theoretical security proof, guarantees that the outputs are unpredictable to any classical or quantum adversary.

Although unpredictability cannot be verified statistically, we nonetheless ran the standard NIST and Diehard test suites on the amplified string, confirming its expected statistical behavior (see *Results and Discussion*). These tests serve as an additional consistency check.

For example, the author claim that the SV source is tested to support an unpredictability bias \mu below 0.75%. What would be the corresponding value if similar tests were applied to the output bitstring? Additionally, the authors mention in the introduction that "correlations have been found in experimental tests of commercially available random number generators". Are these correlations less significant in the perfect random number generator produced in this work? Including such analyses would substantially strengthen the credibility of the conclusions.

Statistical tests support that the output is uniformly random (corresponding to μ=0) and uncorrelated: the amplified output string passes the NIST and Diehard test suites without anomalies. Thus, from the perspective of state-of-the-art statistical testing, the correlations observed in some commercially available RNGs are absent in our output. However, we emphasize that statistical tests – no matter how extensive – cannot verify unpredictability. Indeed, since predictability is defined relative to a potential adversary and their (to us inaccessible) side information, these tests (or any other experiment) cannot be used to quantify the bias μ of the output bitstring. Rather, it must be inferred using only the experimentally accessible information. This is exactly what is done by the security proof. In particular, the security proof guarantees that, under the assumptions outlined in Supplemental Section V, the output is uniformly random and uncorrelated with any information available outside the laboratory.

To better explain the meaning of the 'supporting an unpredictability bias μ below 0.75%' statement, we added a clarifying paragraph to the 'Amplifying Randomness' section of the main text:

"Because the bias μ quantifies the predictive power of a potential adversary, it is not an experimentally measurable quantity. Nonetheless, the assumption that a source is (μ, ε)-random would be falsified by a statistical test that yields an observed bias larger than~ μ. Therefore, if the observed bias is μ' < μ, we say that the test result is compatible with the (μ, ε)-randomness assumption."

We have furthermore included a Supplementary section IX "Additional dataset" discussing a dataset where one of the two QRNGs was not fully calibrated, leading to a non-negligible probability bias of ~0.2%. There, in agreement with the randomness amplification premise, we observe that the statistical bias disappears in the final output string. Nevertheless, the absence of any possible correlations can be only guaranteed via the security proof.

I find the manuscript somewhat lacking in quantitative detail. The authors claim that the output randomness is perfect for any adversary whose predictability \mu of the input randomness is below 0.75%, yet they do not explain how this specific threshold is determined. Relatedly, while Fig. 2 illustrates the relationship between the tolerated bias \mu, the CHSH value, and the number of trials required, the methods used to derive this relationship and perform the corresponding numerical calculations are not provided. In particular, the influence of the trial number on the results warrants a more explicit explanation.

We thank the referee for pointing out that additional quantitative detail would improve the clarity of the manuscript. In the revised version, we provide the details of the relationship between the protocol parameters – tolerated bias μ, observed CHSH value, number of trials, error parameter \varepsilon, and the final output length – in the Supplemental Material section VIII "Tradeoff between protocol parameters". Likewise, we highlight the influence of the trial number on the results, and the correspondence between the tolerated bias μ and the length of the output string.

Our choice of μ follows this relationship between the protocol parameters -- we choose a bias μ close to the maximum tolerable with our experimental parameters to generate a non-negligible output amplified bitstring.

In Fig. 4, error bars should be included. It is also unclear what value of \mu was used when calculating the \mu-MDL values presented.

In Fig. 4 (Fig. 5 in the revised manuscript), both quantities we plot are not properties of our experimental equipment which are inferred from the data (it would be the case, for example, for readout fidelity), but rather properties of the generated raw data string. As we have a single resulting data string and it is fully known, putting error bars on both quantities would not be appropriate – both S-value and MDL-value are calculated exactly with no associated statistical uncertainty for the output raw datastring. The bias μ is now specified in the caption of the figure.

In Results and Discussion section, the length of the input and final random bitstring is given. The authors should explicitly explain how this randomness extraction procedure is achieved. What are the values of n and d introduced in Box I? And why such a particular choice?

The extraction step follows directly the conceptual protocol of Fig.1 and Box I, and the experimental implementation in Fig. 4 of the revised manuscript. As explained in the Randomness Extraction section of the Methods, we apply a two-process extractor to the full raw data string and the outputs of the SV sources. Our choice of extractor uses additional $d=2n$ bits from the SV source. The output size m is chosen in a way to guarantee the final failure probability ε.

The number of protocol trials n was selected based on experimental practicality: it corresponds to a large but feasible number of trials that our setup can perform in a single continuous run while maintaining stability and calibration, and that already enables a non-trivial demonstration of randomness amplification. While the precise numerical value is somewhat flexible, its order of magnitude is bound from both sides: from below by Fig. 2, since reducing the data volume by an order of magnitude would place us near the boundary of the region where device-independent randomness amplification is possible; and from above by the long-term stability limits of room-temperature control hardware (see Table I in the Supplemental Material).

We added a clarification to the Results and Discussion section of the main text explaining the length m of the output amplified string. We have also added Table IV to the Supplemental Material, uniting all parameters' choices in a single place. Finally, we give extra clarification on the technical implementation of the extractor in the Supplemental Material section VII.

Minor points:

- I am not sure if it is appropriate to classify this experiment as demonstrating "quantum advantage." In some respects, the situation of quantum randomness amplification is similar to quantum error correction - a task that also cannot be achieved through

classical information processing. Yet, we do not typically label quantum error correction experiments as examples of quantum advantage. Therefore, I would recommend using the term "quantum utility" instead.

We thank the referee for this comment and for the suggestion. We agree that in the context of quantum error correction, labeling the achievement as a quantum advantage would be excessive. Our interpretation for the labelling choice is that quantum error correction experiments represent a type of task which is only relevant in quantum information processing, i.e. these experiments are dedicated to solving a problem which arises specifically inside the field.

In contrast, the task of generating high-quality randomness is a fundamentally classical one which even predates the field of quantum information: it can be formulated as a black-box task that takes classical weak randomness as input and outputs classical high-quality randomness. In other words, this task is defined without reference to quantum mechanics, and as the original work by Santha and Vazirani shows, it is impossible to accomplish it classically. From this perspective, randomness amplification represents a genuine instance where a quantum protocol accomplishes a task that no classical process can perform.

We therefore believe that using the term “quantum advantage” is appropriate here, as it fully captures the meaning of the term.

- What is the efficiency of the active all-microwave reset used in this work? Does the reset error impact the results, considering it could potentially introduce correlations in the output bitstream?

The efficiency of the active all-microwave reset is consistent with the Ref. 37 (Magnard *et al.*, 2018), with the qubit e-f manifold population reaching below 0.3% as verified in an independent measurement (the number is likely even lower, but is limited by the precision of the measurement we performed).

Even if the reset error were higher, it would not impact the presented results in any way. It would only potentially introduce correlated errors in the raw outputs of the quantum device (strings A and B), which is perfectly acceptable: we do not make any assumptions about the (absence of) correlations in AB, nor do we assume the trials to be independent and identically distributed. The outputs are fully characterized by their observed μ-MDL value; the subsequent application of the randomness extractor (together with the theoretical verification based on entropy accumulation) ensures that any correlations in the outputs of the quantum devices are gone in the final amplified output bitstring.

- Fig. 3 caption: The abbreviation "QRNGs" is used here without being defined upon its first mention.

- Page 3, line 58: The abbreviation "MDL" is used here without being defined upon its first mention.

Both abbreviations are now defined upon their first mentions.

- The symbol S_{\mu, obs} is frequently used in the manuscript, but its definition is provided quite late in the Methods section. To improve readability, I would recommend introducing its definition earlier in the main text.

We agree that providing the definition earlier would improve readability. Unfortunately, we are already at the length limit for Nature articles (more so for the revised version), so accommodating two additional paragraphs of rather technical definitions is difficult. As a compromise, we clarify the role of S_{μ, obs} in the revised “Amplifying Randomness” (formerly called “Quantifying Randomness”) section while referring to the Methods for a precise definition.

- Page 6, line 50: "an observation of S_{\mu, obs} < 0 certifies \varepsilon-perfect randomness", please check.

This is indeed a typo and should read “S_{μ,obs} > 0” instead. It is fixed in the revised version.

- The symbol \varepsilon is first introduced in the main text as the failure probability, while in the Methods section it appears to denote a metric quantifying the closeness between two distributions. Are these two usages referring to the same concept?

The condition $\frac{1}{2}\|\rho - \sigma\| \leq \varepsilon$ can be understood as saying that \rho and \sigma differ with probability at most epsilon. We added this remark together with relevant citations to the methods (subsection Definition of randomness).

- Eq. 1: What is the meaning of P_{X\Gamma_X}?

P_{X\Gamma_X} denotes the joint probability distribution over the random variables X and \Gamma_X. This is now clarified in the manuscript.

- Page 6, line 48: What is the definition of min-entropy?

This paragraph has been removed in the revised version. The operational significance of the min-entropy is now given as a footnote when the quantity is introduced later in the methods.

- Page 7, line 35: Further explanation on the conditional min-entropy is required. Is this the same as the min-entropy mentioned previously? What is the physical meaning of this quantity? What is the meaning of the symbol \varepsilon here?

A footnote was added to explain the meaning of the conditional min-entropy at the first appearance of the term. A table introducing the various quantities was also added to the Supplemental Information VI and is referenced in the relevant places.

- Fig. 5: "extraxctor".

The typo is fixed in the revised version of the manuscript.

Referee #2 (Remarks to the Author):

In this work, the authors present the first experimental demonstration of device-independent randomness amplification, building on a loophole-free Bell test platform developed by the same group a coupe of years ago. Operating at a trial rate of 50 kHz and achieving a CHSH Bell violation of 2.271 per trial, the protocol can tolerate a bias of up to 7.5e-3 in every input random bit. Over about 9 hours of data acquisition, the authors collected n=20*2^{26} Bell-test trials, from which they extracted 20,431,465 high-quality random bits using 5,368,709,120 (four times of the number of Bell-test trials) biased random bits. This marks a notable amplification of input randomness.

This achievement was made possible through several experimental improvements over their prior work [Nature 617, 265 (2023)] made this possible: (1) reduced photon loss between nodes, enhancing entanglement fidelity and Bell violation; (2) increased repetition rate; (3) shortened readout and data processing time; and (4) improved system stability, evidenced by the absence of timing jitters over multiple days of continuous operation. These technical advances, combined with recent theoretical development, enable the landmark demonstration reported in this work.

The manuscript clearly explains most aspects of the work. However, there are statements that I found unclear or in need of improvements. My comments and questions are as follows:

We thank Referee 2 for the positive assessment of our work and of the manuscript. We’re happy to address the individual comments and questions below.

1) About title: Would it be more informative to include the term "device-independent" in the title? Are there also semi-device-independent protocols for randomness amplification, and are all such protocols necessarily based on Bell tests?

Indeed, we were considering naming the manuscript "Device-Independent Randomness Amplification", but eventually decided to highlight the experimental nature of the work, which lead to the current title. The motivation for that is, strictly speaking, randomness amplification has to be device-independent, otherwise a similar argument to the original Santha and Vazirani paper can be given. However, as there are indeed works on semi-device-independent RA, and due to the similar suggestion by referee 3, we added a sentence highlighting the device-independent nature of the protocol and the term 'device-independent' to the abstract.

2) Fig. 2 in the main text shows that with a high CHSH Bell violation and a large number of trials, a bias larger than 4% can be tolerated. In the meantime, I recall that Ref. [55] (Phys. Rev. Lett. 113, 190402) showed that even very large biases (up to 0.5 in each bit) can be tolerated for a loophole-free Bell test. Can the authors comment on the

source of this apparent mismatch? Besides improvement on extractors, is there anything else required? Also, I noticed that several works mentioned in the plot of the figure are not included in the reference list.

We thank the referee for the insightful question. Indeed, Ref. [55] demonstrates that non-locality can be shown for any $\mu < 0.5$. Our randomness amplification protocol, which is based on the MDL inequality in Ref. [55], shares this feature. As written in the caption to Fig. 2, the main limitation is the extractor which is used to turn imperfect randomness into perfect randomness. If better extractors were available, our protocol could tolerate arbitrary bias $\mu < 0.5$. We demonstrate this in the new Section VIII of the revised manuscript.

In our experiment, the achievable Bell violation is the practical limiting factor rather than the performance of the extractor. Even if better extractors were available, to achieve amplification under large bias μ, one would need a very high CHSH violation (see Section VIII of the revised manuscript).

Furthermore, the revised caption of Fig. 2 now cites all the works shown in the figure.

3) Line 31 on Page 4: The abbreviation "FPGA" appears for the first time in the main text. Please define it on first use.

The abbreviation is now defined upon its first mention on Page 4.

4) Line 50 on Page 4: The sentence "Both quantities are stable ...." may be misleading, as Fig. 4 shows noticeable drifts of both quantities.

We agree that the word "stable" might be misleading here; In the revised manuscript, the sentence was clarified and now reads *"Both quantities show minor fluctuations around a constant mean throughout the duration of the experiment, remaining well above the classical threshold"*.

5) Regarding the first sentence in the caption of Fig. 4 "each point corresponds to ....": The plot in the figure doesn't show these points but only smooth curves, right or not?

Indeed, while the data analysis was done with separate points as per Figure caption, we only were showing curves on the plot. We now replotted Fig. 4 (Fig. 5 in the revised manuscript) to clearly show points in addition to smooth curves used as a guide to the eye.

6) Line 32 on Page 5: I would like to point out the recent work [Nature 642, 916-921 (2025)] for a public device-independent randomness beacon.

We thank the referee for pointing out this related work. We have added the reference to the discussion on randomness beacons in the manuscript. While the reference indeed presents a randomness beacon, it does not employ a randomness amplification protocol, meaning it requires perfect input randomness.

7) To achieve randomness amplification, the author employed the MDL inequality in Eq. (4) on Page 7. However, from the current presentation it is unclear whether the measurement operators were chosen to maximize the violation of the MDL inequality given a specific adversarial bias or to directly maximize tolerable adversarial bias. It would be helpful to include a section in the Supplementary Information detailing the prepared quantum state, the measurement operators used, and the criteria or method for choosing these operators.

We chose the measurement operators to maximize the CHSH value (corresponding to $\mu = 0$), rather than to target a specific adversarial bias. This was done for two reasons: first, the exact level of experimental noise is not known precisely before the data acquisition begins; hence, we do not have the information required to reliably optimize the states and measurements beforehand. And second, as the value of μ used in the protocol is relatively small (0.75%), optimizing the CHSH value instead of the MDL value will still yield almost optimal states and measurements. As we use standard measurement operators for a Bell test, the description of the protocol in the 'Experimental Setup' section of the main text (see also Fig. 4 of the revised manuscript) should be correct and complete.

As a side note, I would like to point out that using the MDL inequality is not the only way for addressing adversarial biases in measurement setting choices. Building on the convex-polytope structure of the set of MDL correlations characterized in Phys. Rev. Lett. 113, 190402 (Ref [55] of this work), alternative methods are available. For example, Section 3.4 of arXiv:1806.04553 provides a general treatment, and Phys. Rev. Lett. 124, 010505 presents a specific implementation.

We thank the referee for pointing out other approaches to randomness amplification. These offer different strengths and weaknesses. The paper on quantum probability estimation (QPE, arXiv:1806.04553) is an alternative theoretical framework to the entropy accumulation theorem (EAT). Similar to the original EAT, it requires a Markov-chain condition on the adversary's side information. In the revised security proof against fully quantum side information, we need the generalized EAT which has a weaker non-signaling condition. As a result, the QPE framework cannot be directly applied to our scenario. Furthermore, Phys. Rev. Lett. 124, 010505 assumes the availability of perfect randomness, and hence does not demonstrate randomness amplification. We discuss the relation between the QPE and EAT frameworks in Section VI of the Supplementary.

8) Line 35 on Page 7: Please explain the smoothness parameter \epsilon.

Broadly, the smoothing parameter can be seen as a fraction of the overall failure probability. Introducing all quantities in detail is not feasible in the main text due to length constraints. As a compromise, the different quantities are now formally defined in Table III of Supplemental Information VI. The table is referenced in the relevant places.

9) Lines 38-39 on Page 7, the sentence "This means that our protocol remains secure even if the adversary learns the outputs of the SV source.": I believe the security

requires that the adversary learns the outputs of the SV sources only after the protocol implementation. If the authors agree, please clarify this.

This was indeed a requirement using the original security proof based on the original entropy accumulation theorem. The revised security proof makes use of the generalized entropy accumulation theorem where no such assumption is required.

10) Please specify the base of all logarithmic expressions used in both the main text and Supplementary Information.

The base of the logarithms are now specified.

11) Line 73 on Page 9: Please define AWG at first occurrence.

The abbreviation is now defined upon its first mention on Page 9.

12) Lines 38-41 on Page 12, the sentence "we install an oscilloscope at each node (part of the trusted setup), to detect the arrival time of specific reference pulses output by electronic devices in the setup (part of \lambda)": Does the inclusion of reference pulses in \lambda imply that they are not trusted?

The device λ is indeed not trusted, as specified in the main text (see, for example, Fig.1 and 4 in the revised manuscript), and outlined in detail in Supplemental Information section I and Fig. 6. However, as a part of external verification, we still need to ensure the closure of the locality loophole (which is possible to do in a device-independent manner) – hence we monitor various timing reference pulses generated by room-temperature electronics. Note however, that the QRNGs in the setup are considered part of the trusted setup, and we nevertheless monitor their synchronization both between the data acquisition rounds and on-the-fly during the data acquisition.

13) The subtitle of the left panel in Figure 8 may need a bit of clarification.

We replotted the left panel of Figure 8 (Figure 9 in the revised manuscript) and replaced “70k stability” by the clearer “Local AWG output stability” title.

14) Line 29 and Table II on Page 13: Are there any uncertainties associated with the measured values? Are these measured values constant across trials?

The standard deviation of the timing budget is on the order of a few tens of picoseconds, and that of the protocol duration is on the order of a few hundred picoseconds – both well below the achieved critical timing margin. We have added a statement on that in the Supplementary Information.
The timing analysis follows the same methodology as described in *Storz et al., Nature 617 (2023)*, which includes more information in its Supplementary Information.
Throughout the randomness amplification experiment, we have continuously monitored the relevant metrics, as described in Figures 8 and 9 of the manuscript. As indicated there, the measured values show noise on the level below 100 ps, and no jumps or large drifts – an observation consistent with the above statement.

15) Assumption 5 in Section V: Please elaborate on the motivation or justification for this assumption.

This assumption was slightly weakened as a result of the new security analysis. The new version of Assumption 5 now requires a more natural non-signaling condition. This is justified since the QRNG is separated from the devices running the Bell test. Section V has been adjusted to reflect the new assumptions.

16) Theorem 1 in Section VI needs clarifications. The description of "step 1" is ambiguous. Please define the protocol clearly and introduce error parameters before using them. Also, please provide an explicit expression for the function \eta.

Theorem 1 has been removed in the revised security analysis.

17) Definition 2 in Section VI: Please clarify what is the Markov model. Under which assumption is this model considered sufficient for security?

This has been removed because, under the weaker assumptions of the revised manuscript, the Markov model is no longer sufficient for randomness extraction. Instead, we use two-process extractors which are introduced in Section VI of the revised manuscript.

18) Theorem 4 in Section VI: Are the error parameters \epsilon_1, \epsilon_2, and \epsilon independent? How to choose these error parameters and the smoothness parameters in order to prove Theorem 5?

Theorem 4 has been removed in the revised version of the manuscript. A table summarizing all parameter choices was added to Supplementary Section VI.

19) Theorem 5 in Section VI: I suggest the authors provide a proof sketch or more details. In addition, can the authors provide the values of each parameter used in their implementation of the randomness amplification protocol?

Theorem 5 has been removed in the revised security analysis. The corresponding Theorem 3 in the revised manuscript is more detailed than the previous Theorem 5. A subsection “Choice of protocol parameters” which summarizes our choice of parameters was added to Supplementary Section VI.

20) Remark after Theorem 5: Please clarify that the adversary can only learn the output of the source (XYZ) after their use in the protocol.

This assumption was indeed implicit in the original security proof. Since the revised security proof uses the generalized entropy accumulation theorem, the adversary is now allowed to learn the outputs XYZ as they are produced.

21) If the security proof is according to Theorem 6 in Section VI, please specify the values of all relevant parameters used in the protocol implementation. Also, please provide an expression for the parameter m_i in the proof of the theorem.

The choice of parameters are now given in table IV of Supplementary Section VI. The parameter m_i is defined in terms of the function m which is given in the theorem statement.

22) A comparison between Eq. (19) and Eq. (22) on Page 16 suggests that Theorems 4 and 5 employ different security definitions: Theorem 4 appears to rely on the trace distance conditioned on success, whereas Theorem 5 incorporates the product of the conditional trace distance and the associated success probability. Please clarify which security definition is used for the final result reported in this work.

Theorems 4 and 5 (of the first version) state the same security definition in slightly different terms. The revised version of Theorem 4 (which is labelled as Theorem 3 in the revised version) uses a more consistent formulation of the security condition. For clarity, we have also adapted to Definition of randomness in the methods section to match with the one used in the later theorem.

23) In Section VII, please provide details regarding the implementation of the chosen two-source extractor. For example, what software was used and what type of classical hardware was employed. Also, please report the time required to complete the extraction.

We now provide the requested information in Section VII of the Supplemental Material. The Julia code implementing the extractor will be available together with the manuscript's dataset after the publication of the paper at a permanent online storage.

Overall, I am impressed by the quality of the results and would be happy to recommend acceptance, provided that the points above are addressed or clarified.

We again thank the referee for the careful reading of our paper, positive evaluation of our work and suggesting numerous improvements. We believe that the revisions prompted by these comments have enhanced the clarity of the manuscript.

Referee #3 (Remarks to the Author):

Summary, conclusions and significance: In the paper titled "Experimental Randomness Amplification", the authors report on the experimental implementation of a Device-Independent Randomness Amplification protocol. Specifically, they consider randomness amplification of weakly random sources (assumed to possess a Santha-Vazirani structure with a bias of at most 0.75%) and report a loophole-free Bell test that allows to amplify this source to produce a string of fully random bits. Overall, the experimental produced a total of over 20 million random bits (random up to a small bias of 10^{-12}) from over 5 billion weakly random seed bits, using over 10^9 trial runs performed in a time of about 9 hours. The experimental implementation has impressive features over previous ones - a substantially high Bell violation (a very impressive CHSH value of about 2.271 in a fully loophole-free setup), high repetition rate to shorten

the data acquisition time, and high multi-day stability of room-temperature control electronics and calibration. In particular, fully loophole-free implementations achieving Mbit-range output bit strings have not been done before. The experiment is thus very significant and given the importance of the task of randomness amplification, will be of wide interest to a broad community of researchers in quantum information and cryptography.

We thank the referee for the nice and detailed summary of our results, and we appreciate their positive assessment of the significance and potential impact of this work.

Data & methodology, clarity and context: The methodology is appropriate, and all necessary data is stated to be available from the corresponding authors upon request. The paper is very well-written, the methodology and conclusions are stated clearly and lucidly.

Originality: On the other hand, it must be noted that the paper does not contain any theoretical novelty - the randomness amplification protocol and the assumptions (Section V in the Supplemental Information) are from Ref. [20] Kessler and Arnon-Friedman "Device-independent Randomness Amplification and Privatization" IEEE Journal on Selected Areas in Information Theory 1, 568 (2020), and the security proof against quantum adversaries (Section VI in the SI) is the one given in Ref. [20] using the extractors in Refs. [53, 58]. The manuscript as such mainly reports an improved loophole-free Bell test from some of the previous works such as Refs. [29, 39] for the task of DI randomness amplification. As such, the manuscript in my opinion does not pass the high bar of suitability for publication in the journal Nature.

We thank the referee for these comments. Indeed, the theoretical analysis in the originally submitted version of the manuscript follows Refs. [20, 53, 58], and the individual experimental components -- high-fidelity entanglement generation, closure of the locality loophole, and high-rate and high-quality qubit control -- have each appeared in previous contexts. However, we consider one of the key points of the present work to lie in the combination and integration of these elements into a single and robust experimental demonstration enabling a task that was previously unattainable: fully device-independent amplification of randomness originating from a weak Santha–Vazirani source.

We illustrate this step in Figure 2 of the main text of the manuscript, where it is apparent how the improvements to the experimental capabilities achieved in the present work, which are also highlighted by the referee in their previous comment, put us in an entirely new regime where this demonstration is possible.

In addition, in the revised manuscript, we have strengthened the theoretical foundations of our work. Specifically, we have significantly overhauled the security proof in Section VI of the Supplemental Material. The major improvement, compared to prior works, is that the updated security proof allows for quantum side information about the SV sources. This substantially strengthens our results since, given that the QRNGs are

quantum devices, it is impossible to justify that the adversary's information is purely classical. To achieve this improvement, we employ the recently developed notion of two-process extractors. In addition, the proof is now based on the generalized entropy accumulation theorem, which yields improved finite-size bounds and allows the outputs of the SV source to be revealed in each round (rather than only at the end of the protocol, as required by the earlier analysis). These improvements also allowed us to double the amount of produced certified randomness from 20 to 45 million bits.

The authors might wish to consider the following comments and suggestions for future.

1) Essentially, the main feature of the manuscript is the achievement of a high generation rate in randomness amplification in a loophole-free Bell experiment. It is unclear whether using the data from other loophole-free Bell tests not cited in the manuscript - such as "Loophole-free test of local realism via Hardy's violation" Phys. Rev. Lett. 133, 060201 (2024) from the Pan group - cannot be used to achieve the Mbit-range randomness amplification reported here, especially for such a low value of the bias (\mu \leq 0.0075) as considered here.

We agree with the referee that there are other loophole-free Bell-tests and Bell-test-like experiments which could be further included and cited. Unfortunately, due to the space constraints, and the need to limit the number of references, we focused on citing the most significant and advanced works relevant to device-independent randomness amplification. In the specific example mentioned by the referee, PRL 133, 060201 (2024), the authors demonstrate the experimental violation of Hardy's paradox. While the inequality used there is similar to the MDL inequality in our Eq. (4), the reported violation is very small (~4.6*10^-4). Such a small violation would not allow producing enough entropy to allow for randomness amplification of any meaningful bias μ, even with very large data volumes. Moreover, the PRL 133 experiment implements a Hardy-type nonlocality test rather than a Bell-CHSH test, and hence it is unclear where it would need to be placed in Fig. 2. For these reasons, incorporating that paper into our comparison would not be appropriate.

We also would like to note, following the previous answer, that the high generation rate is only one feature of our experiment. We consider the very ability to perform randomness amplification, without which it is impossible to speak about the generation rate, to be another key achievement of this work.

Secondly, as a minor point, it is unclear how crucial a fully loophole-free implementation is for the task of DI quantum randomness generation. It has been argued in several places in the literature (see for example Liu et al. "Device-independent randomness expansion against quantum side information" Nature Physics 17, 448-451 (2021) and Pirandola et al. "Advances in Quantum Cryptography" Adv. Opt. Photon. 12, 1012-1236 (2020)) that in DI-QRNG experiments (as opposed to DI-QKD experiments), it may not be strictly necessary to close the locality loophole. DI-QRNG experiments are instead considered as performed by a single user Alice who has a secure laboratory and can prevent any devices from receiving or sending communication at will (given that the devices are assumed to be shielded and not leaking any output bits to the adversary,

failing which any DI protocol is rendered insecure). The important thing is to ensure that, before giving its output, each device only learns its own input and not that of the other device which can be ensured by shielding the devices. This is sufficient because the aim of the present experiment is not to disprove local realism. In this context, other experiments that do not close the locality loophole such as those cited in Fig. 2 as well as others such as "Practical No-Signalling proof Randomness Amplification using Hardy paradoxes and its experimental implementation" in arXiv: 1810.11648 should also be taken note of.

The referee raises an important point concerning the relevance of locality and shielding assumptions in different device-independent tasks.

In device-independent quantum key distribution, it is essential that the generated key remains secret. This already requires introducing a shielding assumption, namely, that the devices do not leak any unwanted information to the outside world. Once this shielding assumption is made, it effectively provides everything that would otherwise be guaranteed by enforcing locality. In fact, locality can be seen as a “time-restricted” form of shielding; it is therefore a strictly weaker requirement, as the standard shielding assumption must hold for arbitrary durations. Consequently, there is no need to assume locality in addition to shielding.

By contrast, device-independent randomness amplification can also be meaningful in scenarios where the generated randomness does not need to remain secret (for instance, in a public lottery). In such cases, the shielding assumption is no longer necessary, and the weaker locality assumption becomes the relevant one. The appealing feature of the locality assumption is that, unlike shielding, it can actually be enforced in practice - it follows directly from relativity together with proper timing in the experiment (i.e., by ensuring space-like separation, as realized in the setup used in this work).

In our implementation, we only assume that the SV sources do not receive any input from the Bell test. However, even this assumption can, in principle, be replaced by non-signalling assumptions and appropriate placement of the SV sources. They would need to be quite far from Alice and Bob, but since only classical information needs to flow from the SV sources to the devices, there is no experimental challenge here. Therefore, the randomness amplification protocol does not fundamentally require any shielding assumptions, in contrast to QKD.

To make this important distinction clearer in the manuscript, we have added a discussion to the Methods section on loopholes.

2) It would be good to clarify already in the Title, Abstract and Introduction that the experimental implementation is of a Device-Independent protocol, since there are semi-device-independent randomness amplification protocols in the literature (see for example "Semi-device-independent full randomness amplification based on energy bounds" by Senno and Acin in arXiv:2108.09100).

Following this suggestion, and a similar one by Referee 2 (see “1)” by Ref. 2), we have added a clarifying sentence to the abstract explicitly mentioning the device-independent nature of the protocol there.

It would also be good to clarify in the section on "Quantifying Randomness" that it is assumed that the eavesdropper's side information ‘e’ about the bits from the SV-source is purely classical, i.e., no quantum side information about the source is presumed here.

One of the major improvements of the revised security analysis is that we can now allow for quantum side information about the source. The section “Quantifying Randomness” (now called “Amplifying Randomness”) was updated to reflect this more powerful adversary.

3) The reference list on DI randomness amplification is far from complete, especially a number of papers showing security against no-signalling adversaries as well as papers on amplifying min-entropy sources are not cited.

We have added a discussion about possible directions in which assumptions for device-independent randomness amplification can be weakened to Section V of the Supplementary. There, we discuss prior works on non-signaling adversaries and min-entropy sources.

It would also be interesting to check whether the generation rates can be increased with improved quantum-proof extractor constructions such as the one by Foreman et al. in "An efficient construction of Raz's two-source randomness extractor with improved parameters" arXiv: 2506.15547.

The new security analysis against quantum side information requires two-process extractors rather than the two-source extractors in the Markov model considered by Foreman et al. It is currently not known whether Raz’s construction also works as a more general two-process extractor. Hence, we stick to Dodis et al’s construction. However, we agree that the possibility of improved extractors (such as Raz’s construction) is an important open question. We discuss this explicitly in the revised Section VI of the Supplementary.

4) Minor typos: in the Methods section in page 6, shouldn't it be S_{\mu, obs} > 0 allows to prove the independence condition and certifies randomness? In page 3, it is stated that the source has been extensively tested, and that the results are compatible with a bias below 0.75% - but given that the bias quantifies the predictive power of an adversary, it cannot be estimated by any statistical test, and as such the claim that it is below 0.75% may be confusing to the reader.

$S_{\mu,obs} < 0$ was indeed a typo and has been fixed in the revised manuscript. The referee is correct that the bias represents the predictive power of an adversary, and can therefore not be experimentally verified. However, one can experimentally falsify the claim that the source is random (e.g. by observing a bias in the frequency of zeros and ones). This is why we only claim the tests to be “compatible with a bias μ below 0.75%”.

To clarify the meaning of the bias, we have revised the earlier section on “Quantifying Randomness” (now titled “Amplifying Randomness”).

Referee #4 (Remarks to the Author):

Summary of the key results

This paper presents an experiment for randomness amplification, the task of transforming imperfect random sources into truly random outputs. The task is essential to many applications using randomness. For example, in modern cryptography, it is known that if the security keys are generated using imperfect random bits, the public information becomes sufficient to recover the secrets.

Verifying whether a source produces unpredictable bits can not be done by observing (the statistics of) the samples since it is an inherent property of the device. More concretely, it is known that one cannot certify randomness generated by a purely classical device. In contrast one can present a protocol that certifies randomness via a test that accepts a quantum device and meanwhile rules out classical ones. Therefore known protocols for this task are tied to demonstrations of quantum advantage.

Previously there has been multiple experiments (e.g., Ref. [29]) that demonstrate violations of Bell's inequality, tests that demonstrates quantum entanglement sharing between two devices out of communication. These experiments, while closing all other loopholes, require an assumption that the inputs to these devices must be uniformly random bits. This work builds upon the experiment in Ref. [29] and the theory in Ref. [20], Ref. [53], and Ref. [58] to further remove this assumption. In particular, this paper shows that if the inputs are weakly correlated in the sense that they are sampled from Santha-Vazirani sources, one can still produce certifiable random bits experimentally.

Originality and significance

The theory part appears to be fully drawn from pervious results (Ref. [20], Ref. [53], and Ref. [58]), and the main contribution in this paper is the upgrade of the experiment in Ref. [29] (which only demonstrates a violation of Bell's inequality) for the first experiment for randomness amplification. As far as I can tell, the security proofs (Supplementary Material, Theorems 5-6) are only straightforward combinations of previous results, and the theory is no particularly tailored for the experiment.

As a theorist, I cannot tell how difficult these experimental improvements are to achieve, but I will summarize them anyway. First, this new experiment not only demonstrates a violation of Bell's inequality (as in Ref. [29]) but also demonstrates certifiable randomness amplification. The number of trials (rounds) are significantly increased from a million to 1.34 billion trials; therefore the completion of a single round is more efficient. The experiment also adds Santha-Vazirani sources as inputs to the devices, with the level of predictability \mu <= 0.75%. It also increase the S value from 2.07 to 2.271. A

higher violations tightens the bound on generated entropy (the entropy per round is a monotonic function in the violation). This protocol uses a two-source extractor in Refs. [61-62] for efficiently concerns. The channel loss is decreased from 19% to about 12-14%. This experiment claims to produce ~20 million random bits in nine hours. Compared to the very recent experimental work using random circuit sampling [27], the generation and verification appears to be very efficient.

We thank the referee for the thoughtful summary of our results and for highlighting the broader context of the work.

We would like to note the improvement to the theoretical framework made during the revision process. Indeed, in the originally submitted version, the security proof was mostly inherited from Ref. [20, 53, 58]. As a result, we also inherited the limitation that the adversary is only allowed to have classical side information about the SV source. This is particularly unsatisfying since the QRNG themselves are quantum devices, thus making it hard to justify that the adversary should only hold classical information about them. Partly motivated by the comments of the Referees and to overcome this limitation, we took the opportunity to overhaul the security analysis in Supplementary Section VI. The revised security proof uses a new notion of so-called *two-process extractors*, which were only recently introduced. The new proof, based on the generalized entropy-accumulation theorem, yields several benefits: it allows the outputs of the SV sources to be revealed as they are produced, accommodates for quantum side information, and provides improved finite-size bounds (see revised Fig. 2).

We appreciate the referee's perspective as a theorist and thank them for highlighting the strength of the experimental advances that underpin our results, enabling the high-efficiency generation and verification of amplified random bits. In the revised version of the work, the improvements to the theoretical analysis described above allowed us to certify an even larger amount of randomness from the same experimental dataset, increasing the final output to 45 million bits (see Results and Discussion).

Data and methodology

I did not spot any error on the presentation at this moment. Since the approach are based on previously known theory, there is no obvious reason to believe there is a loophole on the theory part. The quality of the presentation is quite high, and it is very accessible -- I can understand most of the details in a reasonable timeframe, without reading through previous results.

Compared to Ref. [29], this new experiment adds a Santha-Vazirani (SV) source for the generation of inputs. Since this SV source are implemented using QRNG, I am not sure if this opens a loophole by assuming that the QRNG is SV since the experiment to test the QRNG seems to only heuristically conclude it is SV. I was expecting that the use of quantum sources and their structure (e.g., two devices are out of communication) should be all certified experimentally for a results that claims to be loophole-free. Furthermore, it is said that the bias of the SV source satisfies \mu <= 0.75%, conditioned on the adversary's information. I am not very convinced of how this is

obtained as explained. If it is from some empirical estimation I am not sure whether one can conclude the source is SV.

Suggested improvements

As explained in the Data & Methodology section, I believe that the authors can further explain how they can experimentally conclude that the inputs are SV sources.

We agree with the referee that we do not, and in fact cannot, experimentally verify that the input QRNGs are Santha–Vazirani sources. This property necessarily enters as an assumption of the experiment. Conclusively establishing that a physical device behaves as an SV source would require placing an upper bound on all correlations between its outputs and everything in their past light cones — a task that is fundamentally impossible to perform experimentally (as also discussed in our response to Referee 1). Our assumption, however, is weaker than in all prior loophole-free experiments, which had to assume perfect sources.

We added the description of our input RNGs, and the justification for them being SV sources (based on the excess predictability specified by the manufacturer) to the end of Supplemental Material section I ('Experimental Devices'). Furthermore, to clarify the logic of the manuscript, we reworked the section Amplifying Randomness (formerly "Quantifying Randomness"). We highlight the treatment of the input QRNGs as SV sources explicitly; furthermore, the assumptions made for the experiment are listed in the Supplemental Information section V.

References

The paper offers appropriate credit to previous work.

Clarity and context

The presentation is very clear, and the abstract, introduction, and conclusions well explains the contexts and the results.

We thank Referee 4, and all the Referees, for careful and critical reading and assessment of our work. We trust that our addressing the comments and implementing the suggested changes has made our manuscript clearer and more accessible.

Note from the authors: In the following document we show the referee's comments in black and display our responses in blue.

Referee #1 (Remarks to the Author):

The manuscript has been significantly improved through the authors' revisions, which provide additional support for the theoretical framework and further clarification of the experimental details. The presentation is also clearer. I am satisfied with authors' response to my concerns regarding the soundness of the randomness amplification results. Given that the work demonstrates the generation of randomness beyond the reach of any classical device, it can be described as achieving quantum advantage. Therefore, I am happy to recommend publication.

We thank Referee #1 for their careful reading and feedback throughout the review process, and for their positive assessment of the revised manuscript.

Referee #2 (Remarks to the Author):

I would like to thank the authors for their detailed response and the revisions made to in the manuscript. The authors have addressed most of the points raised in my first report, and the manuscript becomes more accessible to general readers. Furthermore, the authors have upgraded their security analysis. By allowing for quantum side information about the random sources used, they have improved over prior security analyses of device-independent randomness amplification. This significantly enhances the impact of the work. I am therefore pleased to recommend this impressive work for publication in Nature.

We thank Referee #2 for their evaluation of the manuscript, and for their positive assessment of the improvements made in the revised version.

At the same time, I list a few minor points below that I hope the authors can clarify before final acceptance:

1) The abbreviation "QRNG" is not defined before its first use in the paper.

We have added a definition of this abbreviation at the beginning of the Supplemental Information. In the main part, the abbreviation is used in figure captions, where we have also clarified its meaning.

2) Regarding the sentences "... However, in contrast to quantum random number generation, randomness amplification guarantees that the output is perfect provided that a minimal set of assumptions hold (see Supplementary Information V for a detailed list). In the language of Bell tests, this requires the implementation to be loophole-free (see Methods for a discussion of possible experimental loopholes)." on Page 2: This

phrasing is a bit misleading. There are different types of quantum random number generators. Some of them are also based on loophole-free Bell tests and require only a minimal set of assumptions, although the minimum set required differs from the set used in this work.

We have adjusted this sentence to no longer refer to other types of quantum random number generators. In addition, we have added a more detailed discussion on other randomness generation protocols, notably randomness expansion, and their underlying different assumptions to Supplementary Section V.

3) Regarding the response to my previous comment 7): As far as I can tell, the measurement operators are only implicitly mentioned in the sentence "At each node, as in Refs. [35, 36], the random input bit controls the state of a microwave switch which conditionally passes a microwave pulse to rotate the qubit by π/2 around a controlled axis, implementing the measurement basis selection." on Page 5. Consequently, the specific operators performed are still not fully clear. I suggest the authors explicitly list these measurement operators in the main text.

We agree that that the manuscript focuses on the experimental procedure and does not explicitly list the measurement operators. In our experiment, the effective measurement operators arise from a calibration procedure and depend on an angle \theta, which reflects phase references and delays in the setup. As a result, providing a concise and general expression for these operators is not straightforward.

A full description of the calibration procedure, on the other hand, would exceed the scope of the main text. Hence, we refer to Refs. [35, 36], where the calibration and resulting measurement operators are described in detail.

We also note that as our analysis is device-independent, our claims do not depend on the form of the measurement operators, i.e. the description of these operators does not enter the security analysis.

4) Regarding Definition 2 on Page 17: The notation in Eq. (16) is unclear. Could the authors clarify what states are represented on the left-hand sides of these inequalities?

We have added an explanation to clarify the meaning of these states as well as a reference to the table where this notation is introduced.

5) Regarding Definition 3 on Page 18, I have two questions: I) Are there typos in Eq. (19)? My understanding is that the instrument M transforms system A into systems X and S. However, the conditioner in the min-entropy term is listed as systems S and B. Should this not be systems S and A? A similar problem exists for instrument N and its associated min-entropy. II) This definition appears to consider only pure initial states. Could the authors comment on its applicability in practical scenarios where the purity of the initial state is not guaranteed?

Regarding question I), the conditioning cannot be on system A, because this system is provided as input to the channel and therefore no longer available at the channel output where the min-entropy is evaluated. Because rho_{AB} is pure, one can, however, think of B as a coherent copy of A. Regarding question II), we have now added a discussion after Definition 3 on how to apply the definition to non-pure states.

Referee #3 (Remarks to the Author):

In the revised version of the paper on Experimental Randomness Amplification, the authors have addressed the concerns raised in the previous referee report. The authors acknowledge that the individual component — high-fidelity entanglement generation, high-rate and high-quality qubit control, closure of the locality loophole — have each appeared in previous papers, and the key point of the present work is to combine and integrate these elements into a single robust experimental demonstration of SV source amplification. The revised paper is very nicely written and the claim is clear. The references have been improved and are now more comprehensive. The loophole-free implementation of device-independent SV-source amplification achieving Mbit-range output bit strings is of definite high interest to the community. Especially, the combination of high-fidelity entanglement generation and the substantially high Bell violation (CHSH value of 2.271) in a loophole-free setup, and high repetition rates are strengths of the paper.

We thank the Referee for the thoughful summary and positive assessment of our revised manuscript.

1) A main practical weakness of the demonstration is that the initial source is already assumed to be quite close to uniform (each of the 5.4 billion bits taken from the SV source is assumed to be at most 0.0075 away from perfectly random) to obtain 45 million bits that are 10^{-12} away from uniform. This renders figures such as Fig. 13 and Fig. 14 somewhat of an oversell, since evidently even applying a one-time pad with the initial SV source bits would render the photo of the sheep equivalent to noise.

We thank the referee for this comment. While we understand how one might arrive at this conclusion, we disagree with it. The bias (\mu) and the distance to uniform (\epsilon) of a source have drastically different impacts on their suitability for cryptographic applications. We demonstrate this now in an updated Fig. 14, which shows that even a seemingly small bias of \mu=0.75% (corresponding to the threshold on our weak input randomness) makes the random bits unsuitable for use in a one-time pad encryption scheme. We have also expanded the figure caption to clarify the difference between the parameters \mu and \epsilon.

2) In relation to question 1, one is lead to question the set of assumptions claimed in Section V. One reason for the small value of the initial bias tolerated (which was also

pointed out in the earlier refereeing round) stems from the closing of the locality loophole. While closing the locality loophole is experimentally demanding, it is commonly accepted in the community (such as in Liu et al. “Device-independent randomness expansion against quantum side information” Nature Physics 17, 448–451 (2021) and Pirandola et al. “Advances in Quantum Cryptography” Adv. Opt. Photon. 12, 1012-1236 (2020)) that DI-QRNG experiments can and should be run under the assumption of shielding. Clearly, closing of the locality loophole has meant that the value S_{μ} is quite small, so that the maximum tolerated initial bias μ (calculated according to Eq.(60)) is also extremely small. It therefore feels as if the authors have attempted to adapt earlier loophole-free Bell experiments (where closing of the locality loophole is strictly necessary to rule out local realism) to DI randomness generation experiments where such a closing of the locality loophole is overkill.

The authors have attempted to justify this by claiming that they have removed the shielding assumption in comparison to previous experiments. They state in the Section V on Assumptions that the shielding assumption “is required if one wants the random bit string K to remain private after it was produced. However, there are numerous applications where a source of public randomness is sufficient (for instance, lotteries or randomness beacons) and therefore we do not impose this assumption”. This looks very unclear. Isn’t the Shielding assumption still required in the current paper to assume that the devices are not leaking any output bits from the MDL test to the adversary, before these are processed along with other SV source bits to produce the final bit string K? Keeping Alice’s devices spacelike separated is not on its own sufficient to prevent them from leaking output bits to the adversary, unless one assumes that they are shielded from said adversary. Therefore, irrespective of whether one thinks of an application such as a lottery or a randomness beacon where the final bit string K of the protocol is revealed to all, it seems to me that a shielding assumption on the devices is necessary in the DI protocol. The authors might wish to clarify this in the section on the claimed applications to lotteries and randomness beacons.

The updated security proof can tolerate that the random bits from the SV source are leaked as they are being produced. The only shielding assumption that we require (Assumption 4) demands that the Bell device cannot signal to the SV sources. While we need this assumption for practical reasons, it can, at least in principle, be enforced by spacelike separation. We now specifically emphasize this point in Supplemental Section V.

3) A slightly technical question concerns the “Single-round entropy bound” used in Section VI. Security Proof. The authors use the single-round von Neumann entropy bound from Ref. [66] derived for the CHSH inequality. They then bound in Eq. (54) $H(A_i B_i |E_i \tilde{E}_{i-1})$ by $H(A_i | E_i \tilde{E}_{i-1})$. This is a quite bad bound given that the joint outputs (A_i, B_i) can in principle contain up to twice as much entropy as the outputs A_i alone, and seems to be an artefact of the analysis used in Ref. [20] (where the objective was not a practical experiment but a proof-of-principle protocol). Some recent works (see for example arXiv: 2411.04858 or Quantum 7, 1114 or Quantum 8, 1445) have computed the single round min-entropy (and numerically the

von Neumann entropy) for the joint outputs which should result in an increase in the overall number of output bits from 45 million by a potential factor of $\alpha < 2$.

We share the intuition of the referee that directly bounding $H(A_i B_i|E_i \tilde{E}_{i-1})$ can lead to improved performance bounds on the protocol. We have added a short subsection at the end of Supplementary Section VI where we discuss such possible improvements. However, since this is a proof-of-principle experiment, we leave this avenue open for future research.

4) Regarding a comment from the previous report about the lack of theoretical novelty in the work, the authors have now updated the paper to a claim that the updated security proof allows for quantum side information about the SV sources. This is a beautiful theoretical result which will definitely be appreciated in the community.

It would be great if the corresponding result could be included in the current paper to add weight to the theoretical contributions made here. Unfortunately, the result is only mentioned in passing with the section on “Two-process extractors” being quite brief. In particular, Definition 3 and Eq.(19) deserve more explanation regarding the assumptions on $H_{\min}(X|SB)$ and $H_{\min}(Y|TA)$, similar assumption should also be present in eq. (21)? The idea seems to be that processes in space like separated regions are described by product maps (which gives rise to an assumption of independent processes as opposed to independent sources), and one can extract uniform randomness from two such processes if they yield sufficient randomness to begin with.

Some motivation on considering quantum side information on the source may be included given that this is the central theoretical novelty (rather than a contribution since the details are missing here). Apart from a statement that the SV source is quantum so that the adversary can possess systems entangled with it, it is unclear why overcoming this assumption is of high importance for a practical experimental implementation.

Regarding the assumptions on the min entropy, we have now added an explanation for why the entropy conditions in Definition 3 are as stated (see also our reply to point 5 of Referee #2).

Regarding the motivation of considering quantum side information, we note that characterizing adversarial capabilities is notoriously difficult, as it requires forecasting technological progress over the decades-long lifespan typical of cryptographic protocols. Since we cannot rule out a future where adversaries possess the hardware to entangle systems with other parties' devices, assuming adversaries with quantum side information is well-motivated. Indeed, designing protocols that remain secure against such long-term technological shifts is a standard goal in modern cryptography.

We have added a corresponding remark at the end of Supplementary Section V.

Referee #4 (Remarks to the Author):

This version provides a new security analysis that improves over what was offered in the previous version. In particular, the authors consider the setting where the adversary may have access to quantum side information about the quantum Santha-Vazirani source. In this setting, a generalized entropy accumulation theorem (GEAT)--in particular, assuming that the adversary learns part of the output in each round--was used to reduce the entropy accumulation to proving a constant lower bound for a single-round von Neumann entropy. The single-round analysis looks not too different from that in [Kessler, Arnon-Friedman]. While the overall combination specifically for randomness amplification is new, the ingredient was offered in previous papers.

In comparison to the previous version, this one addresses my questions.

We thank Referee #4 for reviewing our revised manuscript, and we are glad they appreciate our improved security analysis.

Other comments:

- Some notations are not properly defined or explained (and I have to guess what they really mean). For example, the state \rho_{Q\wedge xy} seems to mean the classical part is projected onto a particular outcome (i.e., onto a standard basis vector). There are more, and I would think it helps the reader to include all the relevant definitions, even when they look obvious to experts.

We now reference the table of notations explicitly when we first use the notation \rho_{Q\wedge xy}.

- There is no reference for GEAT. I think it's from [Metger, Fawzi, Sutter, Renner] but it would be great if the authors can clarify.

The reference to the GEAT indeed appeared quite late (only when the GEAT was stated in Theorem 2). We now reference the GEAT where it is first mentioned.

- The proofs of Lemma 1 and Lemma 2 use Remark 1, but there is no Remark 1, and the hyperlink goes to Definition 1.

While there is (and was) a Remark 1 with a correct link, the formatting made it rather easy to miss. In the revised version “Remark 1” is now written in bold to make it easier to spot.
